# A Systematic Review of Neuromodulation Treatment Effects on Suicidality

**DOI:** 10.3389/fnhum.2021.660926

**Published:** 2021-06-25

**Authors:** Mehmet Utku Kucuker, Ammar G. Almorsy, Ayse Irem Sonmez, Anna N. Ligezka, Deniz Doruk Camsari, Charles P. Lewis, Paul E. Croarkin

**Affiliations:** ^1^Department of Psychiatry and Psychology, Mayo Clinic, Rochester, MN, United States; ^2^Department of Psychiatry and Behavioral Sciences, University of Minnesota, Minneapolis, MN, United States; ^3^Department of Clinical Genomics, Mayo Clinic, Rochester, MN, United States

**Keywords:** suicide, electroconvulsive therapy, repetitive transcranial magnetic simulation, transcranial direct current stimulation, vagal nerve stimulation, deep brain stimulation

## Abstract

**Introduction:** Neuromodulation is an important group of therapeutic modalities for neuropsychiatric disorders. Prior studies have focused on efficacy and adverse events associated with neuromodulation. Less is known regarding the influence of neuromodulation treatments on suicidality. This systematic review sought to examine the effects of various neuromodulation techniques on suicidality.

**Methods:** A systematic review of the literature from 1940 to 2020 following the Preferred Reporting Items for Systematic Reviews and Meta-Analyses guideline was conducted. Any reported suicide-related outcome, including suicidal ideation, suicide intent, suicide attempt, completed suicide in reports were considered as a putative measure of treatment effect on suicidality.

**Results:** The review identified 129 relevant studies. An exploratory analysis of a randomized controlled trial comparing the effects of sertraline and transcranial direct-current stimulation (tDCS) for treating depression reported a decrease in suicidal ideation favoring tDCS vs. placebo and tDCS combined with sertraline vs. placebo. Several studies reported an association between repetitive transcranial magnetic stimulation and improvements in suicidal ideation. In 12 of the studies, suicidality was the primary outcome, ten of which showed a significant improvement in suicidal ideation. Electroconvulsive therapy (ECT) and magnetic seizure therapy was also shown to be associated with lower suicidal ideation and completed suicide rates. There were 11 studies which suicidality was the primary outcome and seven of these showed an improvement in suicidal ideation or suicide intent and fewer suicide attempts or completed suicides in patients treated with ECT. There was limited literature focused on the potential protective effect of vagal nerve stimulation with respect to suicidal ideation. Data were mixed regarding the potential effects of deep brain stimulation on suicidality.

**Conclusions:** Future prospective studies of neuromodulation that focus on the primary outcome of suicidality are urgently needed.

**Systematic Review Registration:** https://www.crd.york.ac.uk/prospero/display_record.php?RecordID=125599, identifier: CRD42019125599.

## Introduction

The idea of treating neuropsychiatric illness through modulating brain activity has a long-standing history. During the Roman Empire, electric current produced by torpedo fish was used to treat migraine headaches (Francis and Dingley, 2015). In the eighteenth century, electrotherapy was used for treating epilepsy by Erasmus Darwin, the grandfather of Charles Darwin (Gilman, 2008). Electroconvulsive therapy (ECT) was the first non-invasive neuromodulation technique, which proved to be successful for treating depression (Staudt et al., 2019). After the success of ECT, several other neuromodulation techniques were used for various neuropsychiatric conditions. Deep brain stimulation (DBS) was approved by the US Food and Drug Administration (FDA) for patients with Parkinson disease, essential tremor, and dystonia. Initial work suggested that DBS was also beneficial for some patients with depression and obsessive-compulsive disorder (OCD) (Gardner, 2013). Vagal nerve stimulation (VNS) was approved for the treatment of intractable epilepsy and treatment-resistant depression (TRD) (Ogbonnaya and Kaliaperumal, 2013). Transcranial magnetic stimulation (TMS) was approved for TRD and the treatment of OCD (Brunoni et al., 2017).

Suicidality is common in neuropsychiatric disease. However, the effects of neuromodulation techniques on suicidality are not well-studied. Some early studies suggested that DBS was associated with higher completed suicide rates in patients with Parkinson disease (Voon et al., 2008), although this direct association was subsequently challenged (Weintraub et al., 2013). Both TMS and ECT have shown efficacy in TRD, but their effects on suicidality are not clear. To our knowledge, no systematic review has examined the impact of neuromodulation treatments on suicidality in neuropsychiatric disease. Therefore, in this review, we sought to comprehensively survey the literature regarding the effects of neuromodulation on suicidality.

## Methods

### Research Design

**Figure 1:**
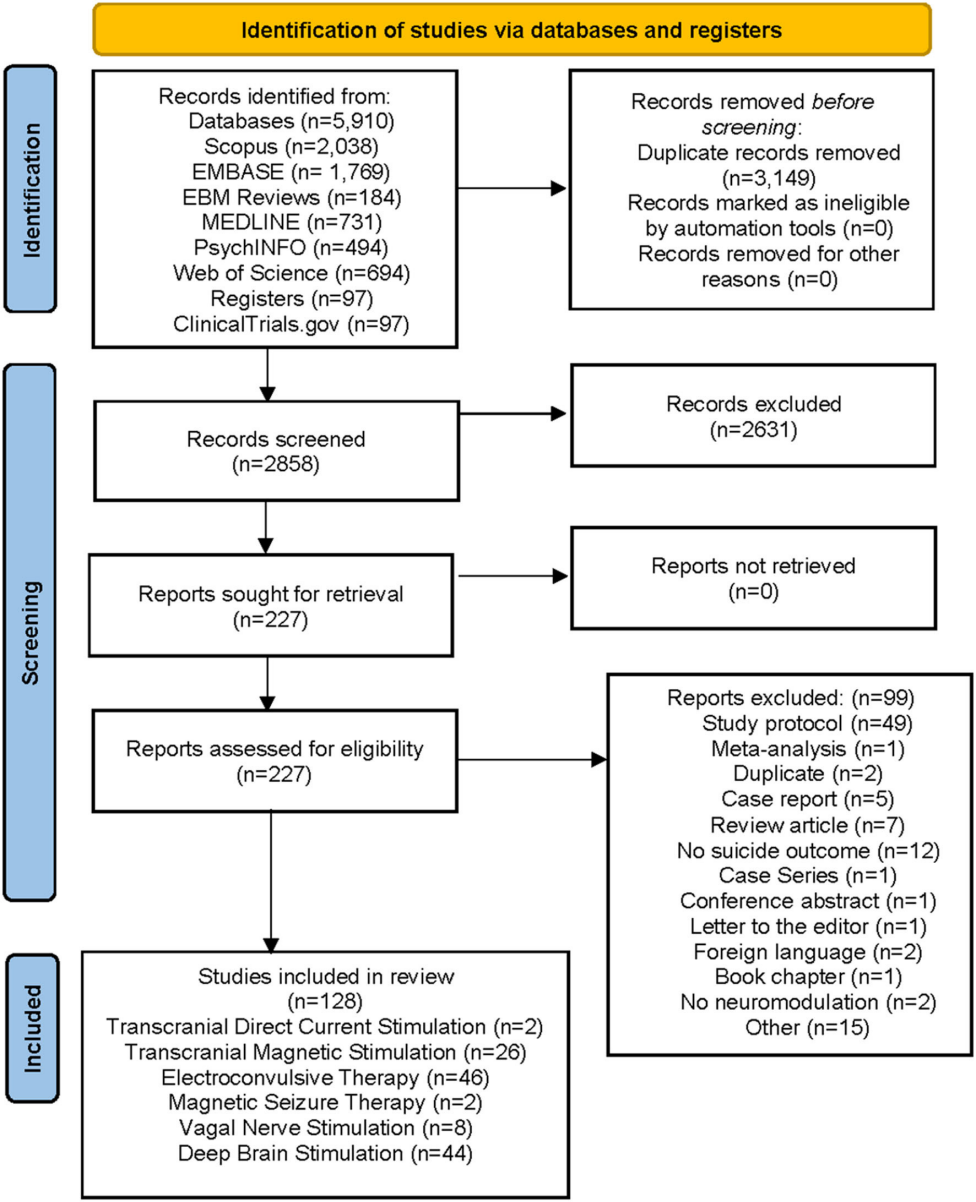
PRISMA Flow Chart.

A review protocol was developed based on the Preferred Reporting Items for Systematic Reviews and Meta-Analysis: the PRISMA Statement (Figure 1) (Page et al., 2021). The protocol was registered through PROSPERO.

### Literature Search

A research librarian conducted an electronic search to identify studies for possible inclusion. The comprehensive search included the following databases: ClinicalTrials.gov, Evidence-Based Medicine Reviews, Scopus, EMBASE, MEDLINE, PsycINFO, and Web of Science. We used the following search terms: DBS, deep brain stimulation; ECT, electroconvulsive; electroshock; LFMS, low-field magnetic stimulation; MST, magnetic seizure therapy; rTMS, repetitive TMS; tACS, transcranial alternating current stimulation; TBS, theta-burst stimulation; tCS, transcranial current stimulation; tDCS, transcranial direct-current stimulation; tES, transcranial electrical stimulation; TMS, transcranial magnetic stimulation; tPEF, T-PEMF, transcranial pulsed electromagnetic fields; tRNS, transcranial random noise stimulation; and VNS, vagal nerve stimulation. We used the following search terms for suicidality: non-suicidal self-injury, NSSI, self-harm, self-inflicted, self-injurious behavior, self-injury, self-mutilation, suicidal, suicidality, and suicide.

### Study Selection and Data Extraction

Rayyan software (http://rayyan.qcri.org) was used to remove irrelevant or duplicate papers. Then, two authors (A.G.A. and M.U.K.) independently reviewed titles, keywords, and abstracts to screen for relevant studies. Each of these authors independently conducted a full-text review of the remaining papers to determine whether the papers met inclusion/exclusion criteria. Any suicide-related outcome measure reported in the papers qualified as a primary outcome. Any change in a relevant rating-scale score from baseline to the last available follow-up was included. Any disagreement between the two reviewing authors regarding inclusion/exclusion of a study was resolved by consensus with a third reviewing author (C.P.L.). Papers meeting inclusion criteria were searched for additional relevant references. Retrospective studies, open-label trials, and randomized controlled studies were considered for inclusion. Case reports, case studies, reviews, meta-analyses, and conference abstracts were not included. Any study that reported a suicide-related outcome (e.g., suicidal event or ideation incidence, rating scales of suicidal ideation or behavior, dichotomous categorization of presence or absence of suicidal ideation/behavior) was included in the review.

### Risk of Bias Assessment

Risk of bias assessment was done by following the guidelines from the Cochrane Handbook for Systematic Reviews by two authors (M.U.K. and A.I.S.), and conflicts were resolved through discussion with the senior author (P.E.C.) (Review Manager 5.1[Cochrane], 2014 and Higgins et al., 2019).

## Results

### Searches

A total of 2,858 potentially relevant publications were identified. After abstracts were screened, 2,631 articles were excluded because they did not meet inclusion criteria. Of the remaining 227 original articles, 104 met the inclusion criteria. Six additional articles were identified after searching the references of the relevant articles. We later refined our search to include more recent articles. Therefore, a total of 128 studies were obtained for data synthesis and became the study articles for data extraction (Figure 1). All extracted data are summarized in Supplementary Tables 1–4.

### Scales

Various scales were used in the studies to assess suicidal ideation and suicide attempt, most commonly those described in this section. A frequently used scale is the Beck Scale for Suicidal Ideation (BSS), a 19-item tool for assessing how often patients have suicidal ideation and the intensity of the ideation in the week before the evaluation (Beck et al., 1979). The Beck Suicidal Intent Scale (BSI) also evaluates suicidal thoughts, intent, and plans (Stefansson et al., 2012). In the Beck Depression Inventory (BDI), a self-reporting evaluation tool, item nine asks about suicidal wishes and suicidal ideation in the past week (Hamilton, 1960). In the clinician-administered Hamilton Depression Rating Scale (HDRS), item three asks about suicidal ideation, wishes, gestures, and attempts in the past week. These 1-item tools have been shown to be valid in a study comparing 1-item scales to Scale for Suicidal Ideation (SSI) (Desseilles et al., 2012). Item 10 of the Montgomery-Asberg Depression Rating Scale (MADRS) has also been used to assess suicidal thoughts, suicide intent, and suicide plans since a patient's previous clinical visit (Montgomery and Asberg, 1979). The Columbia Suicide Severity Rating Scale (C-SSRS) is a clinician-rated semi structured interview. The C-SSRS is used to question the occurrence and severity of lifetime suicidal ideation and suicide attempts in a population aged 11 years or older (Posner et al., 2011). In groups comprising children and adolescents, the Children's Depression Rating Scale–Revised (CDRS-R) has been commonly used. The CDRS-R is a 17-item, clinician-rated scale, and item 13 questions suicidal ideation and suicide attempts (Poznanski et al., 1984). The Quick Inventory of Depressive Symptomatology–Self-Report-16 (QIDS-SR-16) is another scale that is used to assess suicidal ideation. Item 12 evaluates the presence and frequency of suicidal ideation and the presence of suicide plans and suicide attempts during the previous week (Rush et al., 2003). The Mini International Neuropsychiatric Interview (MINI) has also been used to evaluate suicidal ideation, intent, and attempt (Sheehan et al., 1998).

### Transcranial Direct-Current Stimulation

Two studies described the role of transcranial direct-current stimulation (tDCS) in suicidality, and both included only adult patients (Supplementary Table 1). In a randomized, double-blind, controlled trial 120 patients with an acute major depressive episode were randomized into one of four groups: active sham tDCS/sertraline placebo (*tDCS only*) (*n* = 30), active tDCS/sertraline pill (*n* = 30), sham tDCS/sertraline placebo (*placebo group, n* = 30), and sertraline pill/sham tDCS (*sertraline only*) (*n* = 30) (Brunoni et al., 2013, 2014). Results showed that treatment with active tDCS alone was significantly more effective in decreasing suicidal thoughts than treatment with placebo (*p* < 0.01). Combined treatment with active tDCS and sertraline was also significantly better in improving suicidal thoughts than placebo treatment (*p* < 0.01) (Brunoni et al., 2013, 2014). Phases two and three of this controlled trial consisted of a crossover in which patients who had not responded to sham tDCS treatment received active tDCS treatment and a follow-up period during which patients in all four groups received a maximum of nine active tDCS treatments. There was no increased suicidal ideation or attempts throughout these phases (Valiengo et al., 2013).

### Transcranial Magnetic Stimulation

There were 26 studies examining the effect of TMS on suicidality: randomized controlled trials, 17; open-label trials, 7; and retrospective cohort studies, 2. Of the 26 studies, 24 were prospective, and two were retrospective. Most papers (22) described adult populations; four papers described adolescent populations (Supplementary Table 1).

#### Prospective Studies

Of 26 studies focused on TMS, 22 studies enrolled adult patients, and four enrolled adolescent patients. Several studies reported statistically significant improvements in suicidality in active TMS-treated groups compared with baseline of the cohort studied or compared with the control group.

A randomized sham-controlled study by Baeken et al. (2017) with 50 enrolled patients with TRD showed that responders had significantly lower suicidal ideation than non-responders (*p* < 0.05), as measured by SSI. Correlation between improvement in depressive symptoms and improvement in suicidal ideation was also significant (*p* < 0.05) (Baeken et al., 2017). Several other randomized controlled trials have shown the benefit of TMS treatment compared either with a sham control group or with baseline. In a randomized controlled trial with 73 patients, 40 patients were assigned to an ECT group, and 33 patients were assigned to an rTMS group. ECT was significantly more effective in decreasing suicidal ideation than rTMS as measured by the BDI subscale, although both treatments resulted in substantial improvement in suicidal ideation (Keshtkar et al., 2011). Another sham-controlled randomized trial included 108 patients receiving sequential, bilateral (*n* = 52), or unilateral (*n* = 56) rTMS for TRD and 48 control patients matched for age, sex, and diagnosis receiving sham treatment (Weissman et al., 2018). The study showed a greater decrease in suicidality with bilateral rTMS than sham (*p* = 0.02). Suicidality was evaluated using the suicide item of HDRS. Of note, the sham-stimulation group had significantly higher HDRS scores at baseline than the bilateral-stimulation group. A randomized crossover study showed a significant decrease in suicidal ideation (*p* < 0.01) as measured by BSI scores, independent of the order of active and sham treatment. Improvement in suicidality was independent of response to depression, as measured by a 50% decrease in HDRS scores (Desmyter et al., 2016). Another study with the same crossover design confirmed these results (Desmyter et al., 2014). Although suicidal ideation improved (*p* < 0.01) as measured by BSI, the improvement was not related to the order of active and sham treatment or to the change in HDRS score.

Supporting the results above, a sham-controlled trial by O'Reardon et al. (2007) showed that suicidality was more common in a sham group, as measured by HDRS (*n* = 10, 1.9% in the sham vs. *n* = 1, 0.6% in the active-treatment group). Exacerbation of depression was also more common in the sham group (O'Reardon et al., 2007). A randomized controlled trial of 161 patients with TRD, which compared unilateral or bilateral effects of rTMS with a sham control, showed that suicidal ideation was more common in the sham group than the unilateral and bilateral-stimulation groups (2.4% vs. 0%, respectively) (Blumberger et al., 2016). Remission rates were higher in the bilateral-stimulation group than the sham group, but remission was not significantly different between unilateral stimulation and sham treatment.

Two studies comparing the efficacy of accelerated TMS to standard TMS also showed the significant improvement in suicidal ideation from baseline even though groups did not differ in terms of improvement. Fitzgerald et al. (2020) compared the accelerated rTMS treatment and standard rTMS treatment in terms of clinical efficacy in patients with TRD. In a 2-group, single-blind, randomized, controlled trial, they enrolled 36 patients in an accelerated intermittent TBS group and 38 patients in a standard rTMS treatment group. Suicidal ideation was not significantly different between the accelerated and standard rTMS groups, as measured by C-SSRS and MADRS item two scores. However, both groups significantly improved in intensity of suicidal ideation (*p* < 0.001) (Fitzgerald et al., 2020). Response and remission rates were not significantly different between the two treatment groups, as measured by MADRS, either at 4 or 8 weeks after treatment. In a similarly designed study, Fitzgerald et al. (2018) compared accelerated rTMS to standard rTMS treatment for 119 patients with major depressive disorder (MDD) to evaluate the clinical efficacy of different treatments. Improvement in suicidal ideation was not significantly different between the treatment groups, as measured by SSI (*p* = 0*.6*8), although both groups showed significant improvement in suicidal ideation (*p* = 0.02). Response and remission rates were not significantly different between any time points, as measured by HDRS and MADRS, but both groups improved significantly compared with baseline when HDRS was considered (Fitzgerald et al., 2018).

In contrast to results above, Rao et al. (2019) evaluated the antidepressant effect of active rTMS in a randomized sham-controlled study that included 34 depressed patients after traumatic brain injury. They found no significant difference between active and sham-treatment groups regarding change in suicidal ideation (*p* = 0*.4*0), as measured by SSI. At baseline, groups also did not differ in number of suicide attempts (*p* = 0*.4*1) (Rao et al., 2019). Similarly, Baeken et al. (2019) enrolled 45 patients with TRD in a randomized, double-blind, sham-controlled, crossover trial to active intermittent theta-burst stimulation (iTBS) and sham iTBS. Results did not show any significant difference between the active and sham groups for decrease in suicidal ideation, as measured by BSI, either in the first or second week of the study (*p* = 0*.5*3 and *p* = 0*.1*7, respectively) (Baeken et al., 2019). Another study compared active and sham repetitive TMS (rTMS) groups for 42 patients with suicidal ideation and showed a greater decrease in BSS (Beck Scale for Suicidal Ideation) scores for active rTMS than for sham TMS, but this result did not reach statistical significance (*p* = 0.054) (George et al., 2014). However, the active-treatment group had a significantly lower rating for “being bothered by suicidal thoughts.” Nevertheless, at 6 month follow-up, the active and sham TMS groups were not significantly different regarding number of patients with suicidal ideation and attempts.

Two studies presented results in terms of the number of patients with suicidal ideation, gestures, attempts, or completion. In a randomized controlled trial of 212 patients with TRD, two patients in the sham group (of 102 patients) attempted suicide (Levkovitz et al., 2015). Another randomized sham-controlled trial by Yesavage et al. (2018) showed that three patients in the active-treatment group and four patients in the sham-treatment group had suicidal ideation (Yesavage et al., 2018). In another randomized sham-controlled study that included 92 patients with TRD, 47 patients had TMS treatment over 4 to 6 weeks, and 45 patients had the sham treatment over the same period (Carpenter et al., 2017). Patients were followed up for 1 month after the last treatment. Notably, one patient in the sham-treated group completed suicide. In a randomized, double-blind crossover trial of 47 patients with TRD, one suicide attempt was reported in the sham group 1 week after sham stimulation (Desmyter et al., 2016), and in another randomized sham-controlled trial of 180 patients with TRD, no suicides were reported in either treatment group (George et al., 2010).

One study looked at the clinical efficacy of iTBS in posttraumatic stress disorder (PTSD). This randomized sham-controlled study and a subsequent follow-up study of 46 patients with PTSD evaluated the long-term efficacy of active iTBS in improving symptoms of PTSD. No cases of completed suicide occurred either in the active-treatment or sham-treatment group. During 1 year follow-up, overall clinical outcomes were better in the active-treatment group, the relapse rate was lower and the duration between treatment and relapse was longer in the active-treatment group (Petrosino et al., 2020).

Various other cohort and open-label studies described interventions for patients with MDD or a major depressive episode. A prospective cohort study by Hadley et al. compared suicidal ideation, as measured by SSI, between before treatment and after treatment for 19 patients with current major depressive episode. Results showed that 5 weeks of rTMS treatment resulted in a significant decrease in suicidal ideation within the first month (*p* = 0.03) and second month (*p* = 0.004) of treatment, as well as throughout the study (*p* = 0.001) (Hadley et al., 2011). Similarly, a cohort study by Berlim et al. (2014) in which they compared before and after TMS suicidal ideation ratings for 17 outpatients with TRD, showed that 4 weeks of daily deep TMS resulted in a significant decrease in suicidal ideation ratings (*p* = 0.02), as measured by SSI. They also found a significant decrease in depression symptoms as measured by the 21-question HDRS (*p* < 0.001) (Berlim et al., 2014). An open-label trial by Holtzheimer et al. (2010) described one patient during an accelerated rTMS intervention with increased suicidal ideation, of 14 patients with a current major depressive episode. At baseline, four patients had been hospitalized for a psychiatric reason, and five patients had previously attempted suicide (Holtzheimer et al., 2010).

Of four studies involving adolescents, results were mixed. Suicidal ideation did not improve significantly, as measured by Suicide Ideation Questionnaire (SIQ), before and after treatment for nine adolescent patients with TRD (Bloch et al., 2008). However, another study, using data from three prior open-label protocols, showed a significant decrease in suicidal ideation in a group of 19 adolescent patients with TRD who received adjunctive TMS treatment, but when the results were adjusted for the change in depression severity, they were no longer significant. Suicidal ideation was measured by using item 13 of the CDRS-R and the C-SSRS Intensity of Ideation subscale (Croarkin et al., 2018). The other two adolescent studies measured suicide as a safety parameter. In one of these, which included 10 adolescents with TRD, patients were treated with 30 treatments of high-frequency rTMS treatment over 6 to 8 weeks (Wall et al., 2016). Two of the 10 participants had worse suicidal ideation. Another study with the same treatment protocol, which included eight adolescent patients with TRD, showed improvement in suicidal ideation during the treatment period in three patients who expressed suicidal ideation at baseline (Wall et al., 2011).

A retrospective study analyzed change in suicidal ideation for 332 inpatients and outpatients with depressive and affective disorders who were treated with rTMS by using item three of HDRS (Abdelnaim et al., 2019). Of the patients, 47% had an improvement in suicidal ideation, but 41% had no improvement in suicidality. There was a significant positive correlation between improvement in suicidal ideation and increase in drive, as measured by item seven of HDRS (*p* < 0.001).

### ECT and Magnetic Seizure Therapy

We reviewed 46 ECT studies and two studies of magnetic seizure therapy (MST). Four papers included both adolescent and adult patients. The other 42 papers included only adult patients. Both MST papers were open-label trials. The ECT studies were distributed according to type of article: retrospective cohort studies, 19; case-control studies, 8; case series, 5; randomized controlled trials, 6; prospective cohort studies, 5; and open-label trials, 3.

#### Retrospective Studies

We reviewed 32 retrospective studies (8 case control, five case series, and 19 retrospective cohort studies) in this review (Supplementary Table 2).

A study by Black et al. (1987) showed significant improvement in suicidal ideation and suicide attempts for a group of patients with MDD and schizoaffective disorder who were treated with ECT vs. groups treated with adequate and inadequate antidepressant therapy or no treatment (*p* = 0.001, *p* = 0.01, and *p* = 0.01 for suicidal ideation and *p* = 0.001 between ECT and all three groups for suicide attempts) (Black et al., 1987). Parallel to the study results above, comparison of ECT and antidepressant treatment in 519 patients with depression showed a significantly lower rate of suicide attempts in the group treated with ECT (0.8%) vs. the groups treated with inadequate antidepressant treatment (4.2%) or adequate antidepressant treatment (7.0%, *p* < 0.01) (Avery and Winokur, 1978). A total of 17 suicide attempts occurred during the study period.

In contrast, another study using the same population in the last study mentioned above, failed to show any significant difference between treatment groups. Five groups of hospitalized depressed patients were followed up for 3 years after discharge: ECT, adequate antidepressant treatment, inadequate antidepressant treatment, treated with ECT and an antidepressant, or none of these treatments (Avery and Winokur, 1976). Interestingly, when all of the adequate treatment groups (ECT, adequate antidepressant treatment, and ECT plus adequate antidepressant treatment) were compared with the inadequate plus no-treatment groups, no significant difference was found for number of suicide completions at 1 or 3 year follow-up. During the study, there were eight suicide completions. Similarly, another study used follow-up data from 1,076 hospitalized patients with MDD (Black et al., 1989). There were four treatment groups, which were determined by the treatment at index admission: ECT, adequate antidepressant treatment, inadequate antidepressant treatment, and no treatment. In this study, 33 persons completed suicide; 25 (69.4%) occurred in the first 2 years of follow-up. No significant difference was found between the treatment groups with respect to completed suicide rates (*K*^2^: 0.944, degrees of freedom [*df* ]: 3).

Two other studies showed higher rates of completed suicide associated with ECT. Jorgensen et al. (2020) using the Danish National Patient Registry, examined data of 92,895 patients with single or recurrent depression. They found that 5,004 patients were treated with ECT and that ECT treatment was associated with subsequent risk of completed suicide. Adjustment for the possible confounders decreased the risk, but it remained significant (Jorgensen et al., 2020) (Supplementary Table 2). The increased risk in completed suicide was less with increased severity of depression. Similarly, follow up of patients evaluated 25 years after ECT had a higher but not significant completed suicide rate (relative risk [RR]: 1.20 [95% CI, 0.99–1.47]) (Munk-Olsen et al., 2007). However, when the analysis was limited to the first 7 days after the last ECT treatment, the difference became significant (RR: 4.82 [95% CI, 2.12-10.95]). The risk of completed suicide decreased to the levels in the non-ECT group 4 weeks after the last ECT.

One retrospective study examined data of children and adolescents treated with ECT (Mitchell et al., 2018). The patients were evaluated after 6 months of ECT treatment. Results showed that 78.3% of patients reported mild or no suicidal ideation.

#### Case-Control Studies

There were eight ECT case-control papers that have suicide-related data. In a nested, matched, case-control study including 3,845 patients with MDD and PTSD, the rate of completed suicide was significantly lower in a group treated with ECT than in a group treated with other modalities (2.2% vs. 5.9%, *p* < 0.01) (Ahmadi et al., 2016). The rate of completed suicide was also significantly lower when only the MDD group was considered (RR: 0.28 [95% CI, 0.14-0.54]; *p* = 0.001). Similarly, Bradvik and Berglund (2006) compared 96 patients who had severe depression at their index episode and completed suicide during follow-up with age-, sex-, and diagnosis-matched controls who did not complete suicide. They tried to find the relationship among different treatments and occurrences as well as the seriousness of suicide attempts by examining the patients' treatment histories. When the total sample (suicide completers and suicide attempters) was evaluated, patients treated with ECT had fewer suicide attempts than patients treated with antidepressants (χ^2^: 7.49, *p* < 0.01). Nevertheless, suicide attempts were milder in patients treated with antidepressants than patients treated with ECT (0.25 vs. 0.8, *p* < 0.02). Patients who attempted suicide were not significantly different from those who did not attempt suicide regarding the number of previous inadequately and adequately treated episodes (Bradvik and Berglund, 2006).

A comparison of 89 patients with severe depression who completed suicide and a group of matched controls who did not were not significantly different regarding history of ECT treatment (Bradvik and Berglund, 2000). The number of ECT treatments did not affect the number of suicide attempts substantially (10 of 23 in the suicide group vs. eight of 26 in the no-suicide group). Similarly, a case-control study of inpatients showed no significant difference in the use of ECT for patients who completed suicide and their age-, sex-, and diagnosis-matched controls who did not complete suicide (Sharma, 1999). However, in the control group, there was a longer time between ECT and suicide/discharge (*p* = 0.08), as well as more ECT treatments at the index admission (*p* = 0.09).

Several other case-control studies presented suicide data as numbers of suicide attempts or completed suicides. Some of these studies showed lower suicide rates than expected in the studied population. For example, data collected for an 8 year sample of completed suicide cases showed that 71 patients were being treated with ECT at the time of suicide, corresponding to <1% of the cases. Nine suicides, the mean number of completed suicides per year for patients treated with ECT, corresponded to a rate of 10.8 suicides per 10,000 patients treated. Patients who completed suicide after ECT were older, had higher rates of affective disorder, and had higher rates of previous self-harm than the group of patients who did not receive ECT. Patients in the ECT group were also more likely to be inpatients (Hunt et al., 2011).

In contrast, some of these studies showed a higher standardized mortality ratio from completed suicide. For example, a case-control study of 30 patients who completed suicide showed that 19 patients were receiving ECT before suicide (Youseff, 1990). A 5 to 7 year longitudinal cohort study showed that patients who received ECT at their first hospitalization or who had prior ECT treatments had 21% completed suicide (16 of 76 deaths) (Milstein et al., 1986). A history of ECT treatment did not differ significantly among patients who completed suicide and patients who died of other causes.

There was one study looking at the efficacy of ECT for postpartum depression. Ronnqvist et al. (2019) compared the efficacy of ECT on relapse for 180 patients with postpartum depression who were treated with ECT to a control group of 180 patients without postpartum depression but treated with ECT. In the control group, there were 102 relapses and one case of completed suicide, whereas in the treatment group, there were 73 cases of relapse with no completed suicides (Ronnqvist et al., 2019).

Overall, the case series showed either decreased suicidal ideation or a lower standardized mortality ratio from completed suicide in patients treated with ECT. Four prior case series assessed the effect of ECT on patients with depression and bipolar disorder. Suicide completion was the secondary outcome in these studies. A study by Ahmadi et al. (2018) included patients with MDD and MDD plus comorbid PTSD. Patients were followed up for 1 year after 14 ± 1 sessions of ECT. There was significant improvement in both groups in PTSD and MDD symptoms. There were no completed suicides (Ahmadi et al., 2018). Another study from the National Suicide Prevention Project of Finland reviewed 1,397 completed suicides, and only two of these patients had received ECT treatment (Isometsa et al., 1996). This could suggest that patients treated with ECT have a low rate of suicide completion. One of the patients had recurrent psychotic major depression, and the other patient had severe recurrent major depression. Both patients completed suicide ~3 months after their last ECT treatment. A case series of eight pregnant patients diagnosed with mood disorders and treated with ECT reported resolution of suicidal ideation for five patients (Ray-Griffith et al., 2016). This group of patients had various mood disorder diagnoses including unipolar depression, mixed phase of bipolar illness, and depressed phase of bipolar disorder. A larger case series of 264 patients examined changes in suicidal ideation before and after ECT treatments (Benzoni et al., 2015). Suicidal ideation decreased with ECT treatment among patients in all diagnostic categories.

There was one case series that included adolescent patients. In a case series of 30 female adolescent patients with anorexia nervosa who were hospitalized and treated with ECT due to severe suicidal risk (severe suicidal ideation or suicide attempt), only one patient attempted suicide during ECT treatment, and no patients completed suicide following ECT treatment (Shilton et al., 2020). Depression severity also significantly decreased after ECT treatment (vs. before ECT). Follow-up of 13 patients for 5 to 14 years showed that none of the patients had persistent suicidal symptoms. Of seven with follow-up of three to 13 years, none reported any suicidal, depressive, or eating disorder–related symptoms. However, three patients in the follow-up period reported that they had suicidal symptoms at their last assessment.

#### Prospective Studies

We reviewed 15 prospective studies: six randomized controlled trials, one matched-control study, three open-label trials, and five prospective cohort studies (Supplementary Table 2).

Several randomized controlled trials assessed the effect of ECT on depression and studied suicidality as a secondary outcome and presented mixed results. Keshtkar et al. (2011) compared ECT (*n* = 40) to rTMS (*n* = 33) as a treatment for depression. The authors then evaluated suicidality by analyzing the mean change in suicide subscales of the BDI and HDRS. They showed that both ECT and rTMS significantly decreased the suicidal subscale score of the BDI (*p* < 0.001). However, in the HDRS, patients treated with ECT had a significantly greater decrease in the suicide score than those treated by rTMS (*p* < 0.001) (Keshtkar et al., 2011). Supporting the results above, Kellner et al. (2005) examined data from an ongoing randomized controlled study that compared the efficacy of continuation ECT with continuation pharmacotherapy for 444 patients with unipolar depression. They showed rapid relief of suicidal intent, as measured by HDRS item 3, for patients with MDD treated with continuation ECT compared with patients treated with continuation pharmacotherapy. Of 131 patients receiving continuation ECT to prevent relapse, suicidal intent ratings for 106 patients (80.9%) decreased to 0. This decrease was 2.5 times more likely for patients older than 50 years. A strong correlation from baseline was shown between the improvement in depressive symptoms and improvement in suicide rate (*r* = 0.45, *p* < 0.001). Two patients completed suicide during the study (Kellner et al., 2005). Parallel to the results above, in a study that included 30 patients with severe depressive symptoms treated with ECT, Patel et al. (2006) showed a significant improvement in depressive symptoms [*t*_(29)_ = 8.94, *p* < 0.001] and suicidal intent [*t*_(28)_ = 9.79, *p* < 0.001] vs. 30 matched-control patients treated with psychotropic medications and evaluated with the Brief Psychiatric Rating Scale-24 (BPRS-24) (Patel et al., 2006).

The above results are supported by other studies, but the supporting studies present only numbers of cases. For example, Nordenskjold et al. (2013) compared a group of depressed patients with various diagnoses who were receiving ECT and pharmacotherapy (*n* = 28) with a control group receiving pharmacotherapy only (*n* = 28) and reported the number of suicide attempts (Nordenskjold et al., 2013). In the pharmacotherapy group, three patients attempted suicide and one patient had suicidal ideation. In the combined ECT and pharmacotherapy group, only one incident was a possible suicide attempt. A patient had a motor vehicle accident while intoxicated, but it was not clear whether this accident was a suicide attempt. In contrast, Phase II of the Population Research in Identities and Disparities for Equality (*Population Research in Identity and Disparities for Equality Population Research in Identity and Disparities for Equality* PRIDE) study compared the treatment efficacy of ECT plus medication to a medication-only group of patients with unipolar major depression and reported three instances of suicidal ideation, all in the ECT plus medication–treated group (Kellner et al., 2016). None of these suicidal events were thought to be related to ECT.

We reviewed several open-label trials, most of which showed a benefit of ECT. An open-label ECT trial conducted by Rich et al. (1986) evaluated the change in suicidality and either energy or decreased work/activity of 37 patients with depression and suicidal ideation after they received ECT. The authors concluded that the suicidal ideation score (using suicide items of HDRS) significantly decreased after ECT compared with the initial pre-ECT score (*p* < 0.05 for the suicide plus decreased energy subgroup, *p* < 0.05 for the suicide plus decreased work/activity subgroup) (Rich et al., 1986). Ambade et al. (2009) showed a significant decrease in the suicidal ideation scale (SIS) of 50 depressed patients after ECT treatment (*p* < 0.01). SIS scores decreased to normal levels for all 50 patients after ECT was completed. In a 1 year follow-up study of 50 patients with mood disorders who were treated with ECT and who achieved remission, 17 attempted suicide (Cakir and Caglar, 2017). Eleven in the cohort had a history of suicide attempts. In another open-label study, patients with mixed mania were shown to have a greater reduction in suicidal ideation than patients with bipolar depression after ECT (*p* < 0.001), although significant improvement occurred in both groups (*p* < 0.001) (Ciapparelli et al., 2001). Suicidality was measured by MADRS, which includes items related to suicidal thoughts and intent but not to attempts. The number of ECT sessions did not differ significantly between the two groups. Parallel to the results above, in another longitudinal study that compared suicidality before and after ECT using SIS, ECT treatment resulted in significant improvement in suicidality for five patients with unipolar depression and suicidal ideation (*p* < 0.05) (Kawoos et al., 2018). Suicidality in patients with OCD also decreased significantly (*p* < 0.05) (Supplementary Table 2).

Two studies examined the efficacy of MST in suicidal ideation. In their open-label trial that enrolled 27 patients with TRD, Sun et al. (2016) found a significant decrease in suicidal ideation as measured by SSI scores. TMS-electroencephalographic measurements showed a significant correlation of N100 values of the frontal electrodes with improvement in suicidal ideation (Sun et al., 2016). In another open-label trial, Sun et al. (2018) enrolled 23 patients with TRD and showed that MST treatment resulted in significant improvement of suicidal ideation (*p* < 0.001). Of 18 patients with suicidal ideation at baseline, the ideation completely resolved for eight patients. The investigators also examined neurophysiologic correlates of clinical improvement for depression and suicidality. Results showed a significant increase in cortical-evoked activity (CEA) in frontal central electrodes after MST compared with pretreatment (*p* = 0.04) (Sun et al., 2018). No significant correlation existed between improvement in SSI scores and change in CEA. However, a significant correlation existed between the decrease in long-interval intracortical inhibition (LICI) over the central and frontal electrodes and the decrease in SSI scores (*p* = 0.04). Relevant to this, there was a significant decrease in LICI over the right frontal electrodes when the analysis was limited only to patients with resolved suicidal ideation after MST (*p* = 0.048).

## Vagal Nerve Stimulation

### Prospective Studies

We reviewed eight studies of adults with vagal nerve stimulation (5 prospective cohort studies, one open-label trial, and two randomized controlled trials) (Supplementary Table 3).

In a randomized controlled study, Aaronson et al. (2013) compared patients with TRD who were treated with low, medium, and high VNS dosing parameters. The number of suicide attempts after treatment was higher in the low group than the combined medium and high group (*p* = 0.065). The number of prior suicide attempts was not significantly different between groups (Aaronson et al., 2013). Groups also did not differ in remission and response rates in depressive symptom severity at the end of the study. In a randomized controlled trial by Rush et al. (2005a,b) active VNS was compared with sham stimulation in 235 patients with TRD, and there was one suicide completion within the first 5 weeks of treatment. Response rates were significantly higher in the group receiving active stimulation, but no difference existed in remission rates among groups receiving active stimulation or sham (Rush et al., 2005a).

Prospective cohort studies presented mixed results. In a 5 year observation of a cohort of patients with TRD, patients receiving VNS had a greater reduction in suicidality than patients treated with standard therapies (odds ratio [OR], 2.11 [95% CI, 1.28–3.48]; *p* = 0.04), as assessed with the QIDS-SR-16 item 12 and an investigator-completed suicidality assessment (OR, 2.04 [95% CI, 1.08–3.86]; *p* = 0.03) (Aaronson et al., 2017). The group treated with VNS also had a lower completed suicide rate (1.01 per 1,000 person-years [95% CI, 0.11–3.64] vs. 2.20 per 1,000 person-years [95% CI, 0.24–7.79]). Another follow-up study of patients with TRD reported results for the positive effect of VNS on mortality and suicide rates in the sample (mean for adjunctive VNS was 3.2 years vs. mean for treatment-as-usual at 2.1 years) (Olin et al., 2012). The study showed that although adjunctive VNS had lower completed suicide rates (0.88/1,000) than treatment-as-usual (1.61/1,000), this difference did not reach statistical significance. Nevertheless, reduction in suicidal ideation by adjunctive VNS reached statistical significance (OR, 0.80 [95% CI, 0.68-0.95]) compared with usual treatment. There were 2.1 ± 4.4 lifetime suicide attempts in the adjunctive VNS group and 1.2 ± 2.4 suicide attempts in the usual treatment group.

Some of the prospective cohort studies presented results as numbers of people with completed suicide, suicidal ideation, and suicidal behavior. In a cohort study of 74 patients with TRD who were treated with VNS, two patients had completed suicide by the end of the 2 year follow-up (Bajbouj et al., 2010). One of these patients had no prior suicide attempts, whereas the other had 18 prior suicide attempts. Rush et al. (2005a,b) reported 11 suicide attempts over a 1 year follow-up in a randomized sham-controlled trial of 205 outpatients with TRD treated with VNS (Rush et al., 2005b). Schlaepfer et al. (2008) conducted an open multicenter study of 74 patients with TRD in which they looked for the effect of VNS when used as an adjunct to pharmacotherapy. They continued the study for 1 year and reported two cases of completed suicide during their study (Schlaepfer et al., 2008).

In an open-label pilot study of VNS in 59 adult outpatients who experienced major TRD, three suicide attempts were reported during the 2 year study duration (Nahas et al., 2005).

## Deep Brain Stimulation

The literature search yielded 44 relevant DBS studies (Supplementary Table 4). All studies focused on adults. There were 14 retrospective and 30 prospective DBS studies.

### Retrospective Studies

There were 11 retrospective cohort studies, one case-control study, and two case series. The 11 retrospective cohort studies focused on patients with Parkinson disease and essential tremor (1 study) who were treated with DBS (Kenney et al., 2007). The study by Porat et al. (2009) was the only study to apply inferential statistics; the others presented only descriptive data.

A number of studies showed relatively lower suicidality in patients treated with DBS. A retrospective survey of 5,311 patients with Parkinson disease treated with DBS showed a 0.90% rate of suicide attempts and a 0.45% rate of suicide completions (Voon et al., 2008). Postoperative depression, single status (*p* = 0.001), and history of impulse-control disorders (*p* = 0.002) significantly predict attempted suicide risk. Umemura et al. (2011) reported two suicide attempts of 180 patients with Parkinson disease treated with DBS (Umemura et al., 2011). Soulas et al. (2008) followed up 200 patients with Parkinson disease who were treated with DBS and showed that two patients completed suicide and four attempted suicide. No significant differences existed between the suicidal and non-suicidal patient groups for age, sex, disease duration, or preoperative depressive or cognitive status. No significant relationship was shown between change in stimulator settings and completed suicide. The mean duration to suicide attempt was 10.7 months, and the mean duration between surgery and completed suicide was 19.5 ± 7.2 months (Soulas et al., 2008).

In contrast, several other retrospective cohort studies presented relatively higher rates of suicide attempt or suicide completion. Strutt et al. (2012) compared 22 patients with Parkinson disease who were treated with bilateral DBS with a control group who received non-surgical treatment-as-usual. In the DBS treatment group, one patient completed suicide 3 months after surgery, whereas no patients in the control group completed suicide. The mood of the patient who completed suicide substantially worsened during follow-up (Strutt et al., 2012). In a retrospective cohort study involving 22 patients with Parkinson disease, suicidal ideation appeared to increase after DBS surgery (Porat et al., 2009). Three patients had suicidal ideation before surgery, and 10 patients had suicidal ideation after surgery, resulting in a significant difference in the number of patients with suicidal ideation from before to after surgery (*p* = 0.046). One of these patients did not describe suicidal ideation before surgery and attempted suicide 1 month after surgery. Two months after this unsuccessful attempt, he completed suicide (Porat et al., 2009). Funkiewiez et al. (2004) studied a cohort of 77 Parkinson disease patients treated with DBS and reported that one patient completed suicide 36 months after surgery, and four patients attempted suicide at 2, 3, 5, and 6 months (Funkiewiez et al., 2004). Houeto et al. (2002) documented four patients who had increased suicidal risk as measured by MINI in a cohort of 24 patients with Parkinson disease. This risk was present in three of four patients preoperatively (Houeto et al., 2002). Two retrospective cohort studies included patients with other movement disorders. In a cohort study of 46 patients with essential tremor who were treated with bilateral DBS, one patient completed suicide 7 months after surgery (Borretzen et al., 2014). Kenney et al. (2007) reported one completed suicide in a cohort of 319 patients with movement disorders who were treated with subthalamic nucleus stimulation (Kenney et al., 2007). Finally, the retrospective cohort study by van der Wal et al. (2020) reported results for 25 patients with TRD who were treated with ventral anterior limb of internal capsule DBS and followed up for 1 year during the maintenance phase. One patient in the non-responder group attempted suicide during follow-up (van der Wal et al., 2020).

A case-control study by Giannini et al. (2019) described data for 534 patients with Parkinson disease treated with subthalamic nucleus (STN) DBS. They documented 26 patients who attempted (22 patients) or completed (4 patients) suicide (Giannini et al., 2019).

In a case series of 24 patients with Parkinson disease treated with DBS and followed up for 6 months, three of the six patients who experienced worsening of mood symptoms became suicidal. Unfortunately, they did not specify whether it was suicidal ideation, intent, or attempt. None of these patients had a history of suicide attempts (Berney et al., 2002) (Supplementary Table 4).

### Prospective Studies

There were 30 prospective studies (14 cohort studies, five open-label single-group trials, six randomized controlled trials, and five randomized double-blind crossover trials) that included patients with Parkinson disease, movement disorders, and TRD. Of the studies, only two applied inferential statistics; the others presented only descriptive data.

Several randomized controlled trials reported results for the effects of DBS in TRD. In a randomized controlled trial that enrolled 90 patients with TRD, 60 patients were randomized to active DBS treatment and 30 patients to sham treatment (Carpenter et al., 2017). The sham-treated group included patients who had the DBS device implanted but began active treatment 6 months later than the active-treatment group, after the double-blind phase and at the beginning of the open-label phase of this study. During the double-blind phase, two suicidal events occurred. One patient had increased suicidal thoughts, and the other patient attempted suicide. During the open-label phase, two patients completed suicide in the sham group. In the active DBS group, one patient had increased suicidal thoughts, and another patient had a suicide attempt. A randomized, double-blind, sham-controlled trial of patients with TRD (*n* = 29) by Dougherty et al. (2015) reported that suicidal ideation was more common in patients treated with active DBS than sham. Two of 15 patients in the active-treatment group had suicidal ideation, and none of the 14 patients treated with sham DBS had suicidal ideation.

There are also crossover randomized trials that studied the effects of DBS in patients with TRD. In a randomized, double-blind, sham-controlled crossover trial involving 25 patients with TRD, four patients who were non-responders also had suicide attempts during the optimization phase, which lasted 51.8 weeks (Bergfeld et al., 2016). Two patients had increased suicidal ideation during this phase. One of the two patients subsequently responded to DBS treatment; the other did not. During the crossover phase of the study, two patients (1 in the active DBS group, one in the sham DBS group) had suicidal ideation. Two patients did not respond and completed suicide after exiting the study (1, suicide; 1, euthanasia). Of note, before surgery, seven patients had prior suicide attempts (3.3 ± 1.7 attempts) (Supplementary Table 2).

DBS was also evaluated for patients with movement disorders, mostly for Parkinson disease and a study of dystonia. A 2-phase, randomized, controlled trial by Weintraub et al. (2013) compared the suicidal outcomes (suicidal ideation and suicidal behaviors) in a group of patients with Parkinson disease who were either treated with DBS (*n* = 134) or best medical therapy (*n* = 121). No patients had attempted or committed suicide at 6 months, and no significant difference was found for suicidal ideation (Weintraub et al., 2013). After randomizing into STN (*n* = 147) and globus pallidus interna (GPi) (*n* = 152) in the second phase of the study, one patient in the GPi group completed suicide, whereas only one patient attempted suicide in the STN group. The groups did not differ significantly in the rate of suicidal ideation (1.5% vs. 0.7%, *p* = 0*.6*1). However, proxy symptoms for suicidal ideation evaluated with the Parkinson Disease Questionnaire (39 item) (PDQ-39) (Jenkinson et al., 1997) and the Short Form (36) Health Survey (SF-36) (Hays and Morales, 2001) were significantly higher (Supplementary Table 4) in the best medical therapy and STN group compared with the DBS and GPi groups, respectively. Qualitatively, patients in the DBS group were reported to have more energy and be less tired, calmer, and peaceful (overall more full of life) than patients in the best medical therapy group (Weintraub et al., 2013). Similarly, patients in the GPi group had improved mood, less irritability, and better energy levels than patients in the STN group. In a study by Follett et al. (2010), 299 patients with Parkinson disease were treated with pallidal stimulation or subthalamic stimulation, and there was no significant difference regarding suicidal depression between groups (*p* = 0*.9*9), although depression worsened in the STN group and improved in the GPi group. A randomized controlled trial reported results for 20 patients with generalized segmental dystonia treated with active or sham stimulation in the first 3 months, followed by 6 months of active treatment and follow-up (Volkmann et al., 2012). In this study, one patient attempted suicide 6 months after surgery.

A sham-controlled crossover study of 10 patients with treatment-resistant OCD reported that one patient had suicidal ideation after 6 months of treatment (Huff et al., 2010). This patient had a history of suicidal ideation.

Suicide was studied in several prospective cohort studies of patients treated with DBS for TRD. A cohort study of 11 patients with TRD treated with DBS reported one suicide completion and one suicide attempt during a 12 month follow-up period. Both of these patients were in the non-responder group (Bewernick et al., 2012). For 21 patients with TRD, bilateral subcallosal cingulate cortex stimulation was used for treatment (Lozano et al., 2012). One patient had deactivations of DBS two times. The first time the patient attempted suicide, and the second time the patient had a relapse of depression with increased suicidal ideation. A similar study involving 20 patients with TRD treated with subcallosal cingulate gyrus DBS resulted in the hospitalization of three patients because of suicidal ideation (Kennedy et al., 2011).

The effects of DBS on suicide for movement disorders, mostly Parkinson disease, was also studied in several prospective cohorts. A cohort study involving 72 patients with Parkinson disease treated with STN-DBS showed no significant change in suicidal ideation after surgery (*p* = 0*.8*3), as measured by BDI item nine (Castelli et al., 2006). A cohort study of 63 patients with Parkinson disease treated with STN-DBS reported that two patients attempted suicide at the end of 1 year follow-up (Lhommee et al., 2012). One of the attempts occurred after a device infection required removal of the device electrodes. The other attempt occurred 2 months after surgery. In another prospective cohort study, 150 patients with Parkinson disease were treated with DBS, four of whom attempted suicide (Antonini, 2007). In a 6 month follow-up study of 54 patients with Parkinson disease treated with DBS, no attempted or completed suicides occurred. In a study with a median of 2 years of follow-up (range, 12–52 months), one of 25 patients with Parkinson disease treated with STN-DBS attempted suicide (Kleiner-Fisman et al., 2003). This patient, interestingly, did not endorse symptoms of depression around the time of suicide attempt. Therefore, this suicide completion was considered an impulsive act against a specific situation. Of 49 patients with advanced Parkinson disease treated with STN-DBS, one patient with three preoperative suicide attempts completed suicide postoperatively (Krack et al., 2003). Only one suicide occurred in an STN-DBS group of 99 patients with Parkinson disease during 6 months of follow-up (Smeding et al., 2006).

The association of movement disorders and suicide was also studied in prospective cohort studies. In a report by Burkhard et al. (2004) six of 140 patients with movement disorders who were treated with DBS completed suicide during the follow-up period. The mean time between DBS surgery and death was 3.1 years (range, 4 months−7 years) (Burkhard et al., 2004). Another cohort study of 16 patients with movement disorders treated with bilateral GPi-DBS reported that two patients completed suicide at 3 weeks and 14 months after surgery (Foncke et al., 2006).

Several open-label trials studied DBS-related suicidality outcomes, mostly in patients with TRD. During an 8 week, open-label trial of 21 patients with TRD, there was one case of suicide completion and one case of suicidal ideation (Lozano et al., 2012). The completed suicide occurred immediately after the end of the open-label phase, whereas the suicide attempt occurred during the fourth to fifth week of the open-label phase. Holtzheimer et al. (2012) reported results for 17 patients with TRD who were treated with subcallosal cingulate DBS. In the discontinuation phase of the study, full depressive episodes recurred in three patients (Holtzheimer et al., 2012). Reinitiation of DBS resulted in a substantial increase in suicidal ideation for these three patients, and one suicide attempt occurred within the first week of the active stimulation. However, suicidality for this patient resolved without further adjustment of the parameters. Crowell et al. (2019) examined the efficacy of subcallosal cingulate DBS for 28 patients with depression (20 with MDD and eight with bipolar disorder) in an open-label trial with a long-term follow-up design. They documented eight cases of suicidal ideation and five suicide attempts during 6 years of follow-up. In the group with bipolar disorder, there were 17 suicide attempts and 15 previous suicide attempts (Crowell et al., 2019).

During an open-label trial that included 366 patients with Parkinson disease treated with STN-DBS, only one patient in the DBS group completed suicide in the postoperative period (Williams et al., 2010). This patient had a previous suicide attempt.

## Risk of Bias Assessment

**Figure 2:**
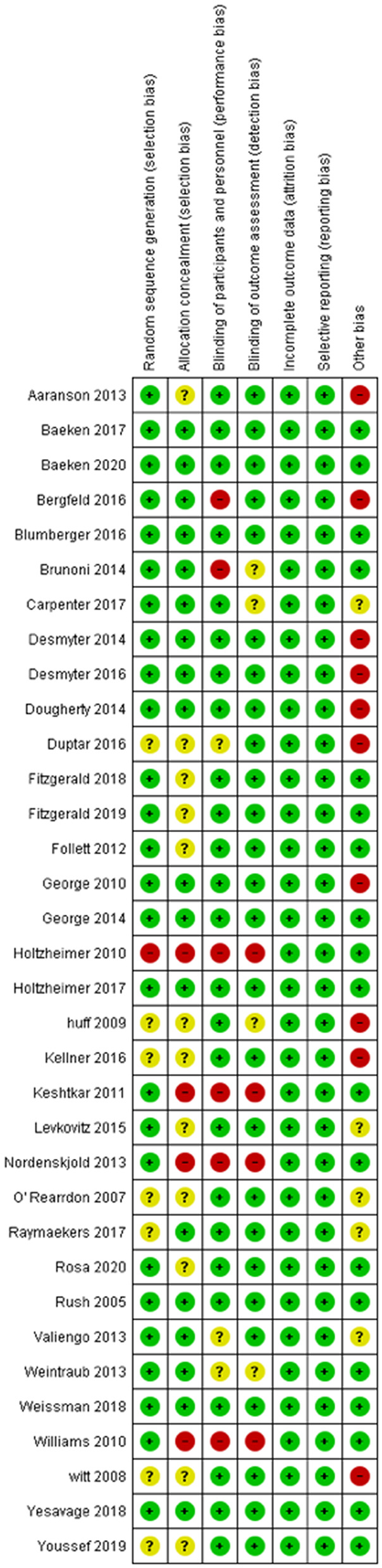
Summary Table for the Risk of Bias in included studies. Randomized controlled studies that are included in our study were assessed by the risk of bias tool by Cochrane Collaboration. This tool allows readers to assess possible risks in five domains including selection, performance, attrition, reporting, and other. Green color denotes low risk of bias, while red color signifies an important risk of bias. Yellow indicates that the risk of bias is unclear, due to insufficient information.

**Figure 3:**
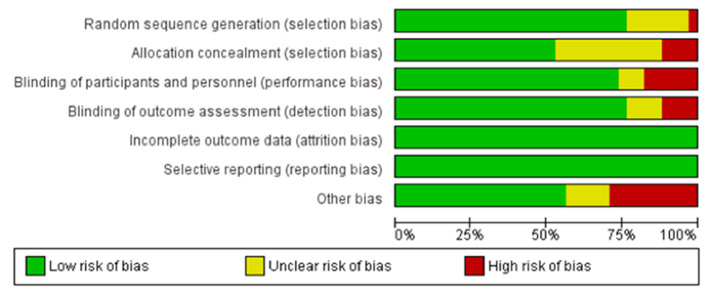
Summary Graph for the risk of bias in included studies. By using the data that is used to create the summary table, this graph gives a broad view of the risk of bias in the included randomized controlled trials in the review. Green color denotes low risk of bias, while red color signifies an important risk of bias. Yellow indicates that the risk of bias is unclear, due to insufficient information.

Risk of bias assessment was done following the Cochrane Handbook for Systematic Reviews guidelines (Higgins et al., 2019). A risk of bias summary and graph were generated via Review Manager 5.1 (Cochrane) (2014) (Figures 2, 3).

## Discussion

To our knowledge, this is the first systematic review to examine the effects of invasive and non-invasive brain stimulation on suicidality. Few studies have been designed to address the prospective impact of brain stimulation modalities on suicidal ideation, suicide attempts, and suicide completion. However, we believe the synthesis of information presented herein will serve as a starting point for hypothesis generation and for crafting well-designed and adequately powered studies to assess the efficacy of neuromodulatory tools on suicidality.

### tDCS Studies

Two studies evaluated tDCS, which were different phases of the Sertraline vs. Electrical Current Therapy for Treating Depression Clinical Study (SELECT TDCS) trial. Phase I of this trial showed the protective effect of tDCS on suicidality compared with placebo. tDCS decreased suicidal ideation significantly (*p* < 0.01) (Brunoni et al., 2014). No suicidal ideation was observed during the crossover (phase II) and follow-up (phase III) phases (Valiengo et al., 2013). The mechanism of action of tDCS remains theoretical, and a more definitive understanding requires further research.

### TMS Studies

Most rTMS studies showed a significant decrease of suicidal ideation. In 12 of these studies, suicidality was a main outcome; 10 (Hadley et al., 2011; Keshtkar et al., 2011; Berlim et al., 2014; Desmyter et al., 2014, 2016; Baeken et al., 2017; Croarkin et al., 2018; Fitzgerald et al., 2018, 2020; Weissman et al., 2018) of which showed a significant improvement in suicidal ideation while two RCTs (Baeken et al., 2019; Rao et al., 2019) could not show any significant difference. In four of the all studies, a positive correlation was shown between improvement in depressive symptoms and suicidal ideation (Bloch et al., 2008; Berlim et al., 2014; Baeken et al., 2017; Croarkin et al., 2018). Notably, in one study, the decrease in suicidal ideation was no longer significant after improvement in depressive symptoms was considered (Croarkin et al., 2018). Thus, it is hard to reach a conclusion if improvement in suicidal ideation is solely caused by improvement in depressive symptoms. Suggested mechanisms for the improvement in suicidality after TMS treatment is discussed below and includes indirect links via improvement in depression and direct links via various neurobiological mechanisms. Almost all studies applied 10 Hz high-frequency stimulation. Initial studies that used TBS showed similar efficacy to rTMS in decreasing suicidal ideation. A study that evaluated deep TMS also showed significant improvement in suicidality ratings (Berlim et al., 2014). Dumas et al. (2012) noted that the TMS frequency was not a factor for predicting treatment response for patients with major depression (Dumas et al., 2012). In addition, no studies have examined the effects of 1-Hz stimulation on suicidality, making it difficult to speculate on the role of TMS dosing in improving depressive symptoms and in improving suicidality.

The inhibitory role of low-frequency TMS and the excitatory role of high-frequency TMS also produced counterintuitive conclusions because suicidality has been shown to be associated with γ-aminobutyric acid (GABA)ergic deficits at many levels (Zhao et al., 2018; Lewis et al., 2019), and 10 Hz or any high-frequency treatment should have worsened this situation from the TMS excitatory effect on the cortex. However, the results instead showed that high-frequency TMS decreased suicidality. Considerable individual variability most likely exists regarding the effects on the brain of various TMS doses. Clearly, the relationship between TMS frequency or dosing in general and the ensuing impact on suicide warrants further careful study. TMS may exert its protective effect by allowing the patient to reassume emotional or cognitive control. As discussed in the paper by George and Post (2011), TMS improves corticolimbic regulatory control over emotional drive (George and Post, 2011). This might help the regulation of the emotional state, a deficiency of which is thought to be responsible for the suicidal state (Jollant et al., 2011).

Nevertheless, studies in the present review included data focused on suicidal ideation, which does not reliably predict suicidal behavior (Franklin et al., 2017; Glenn et al., 2017) and also has very different neural mechanisms (Just et al., 2017). The underlying neurobiological mechanisms of suicidal ideation and suicidal behavior are most likely very different. A study by Just et al. (2017) provides support for the theory regarding the opportunities for TMS enhancement of emotional control. The authors used machine learning to study the changes in neural representations of concepts related to life and death. They found that the degree of evoked emotion, related to the concepts presented, was the major difference between persons with suicidal ideation and control patients (Just et al., 2017). For example, neural correlations of the concept *death* evoked more shame in the suicidal ideation group, emphasizing the role of emotional regulation in suicidal ideation.

Another explanation for decreased suicidality in TMS studies might be the decrease in depressive symptomatology. All studies in our review that examined depressive symptoms showed that improvement in suicidality correlated with improvement in depression. A study by Croarkin et al. (2018) had a similar conclusion, but the data were no longer significant after adjustments were made for depression severity (Croarkin et al., 2018). Psychiatric disorders, especially depression, are important risk factors for suicidal behavior, supporting the possibility that TMS exerts its effect through improving depression.

A comprehensive review of neuroimaging studies suggested that impairments in the medial and lateral ventral prefrontal cortex and connecting structures have a role in negative emotional states and are potential drivers of suicidal behaviors (Schmaal et al., 2020). Dysregulations in the dorsolateral prefrontal cortex and the inferior frontal gyrus are probably linked to suicidal behaviors. The anterior cingulate cortex also likely has a role in regulating suicidal thoughts and behaviors (Schmaal et al., 2020). This work is important as it generates potential hypotheses and discrete targets for interventional studies with brain stimulations.

Multiple other putative mechanisms may be responsible for the effects of TMS on depressive symptoms and suicidality: neurotrophic factors and synaptic plasticity, abnormalities in the hypothalamic-pituitary-adrenal axis, abnormalities in serotonin, and dysregulation in GABAergic-inhibitory mechanisms and glutamatergic-excitatory mechanisms. TMS has been consistently shown to increase brain-derived neurotrophic factor in stimulated and distant brain regions, thereby inducing plasticity (Chervyakov et al., 2015). Plasticity and increased regeneration might be a way TMS affects suicide, when the effect of aging-impaired neuroregeneration is considered in depression (Jacobs et al., 2000). Hypothalamic-pituitary-adrenal axis dysregulation has also been shown to be a target of TMS treatment. Rats that were exposed to chronic, unpredictable stress and subsequently developed depression also had high adrenocorticotropic hormone and cortisol levels (Zhao et al., 2018). TMS treatment reversed the abnormality in the hypothalamic-pituitary-adrenal axis and improved depression in the rats.

#### Neurotransmitter Studies

Regarding GABA-related mechanisms, abnormalities in GABAergic function affect suicide and depression. Lewis et al. (2019) showed a significant positive correlation between change in GABAergic-inhibitory measures of TMS and change in suicidality, even after controlling for the effect of change in depression. In a genotype and transcriptome study by Yin et al. (2016) the investigators showed that GABA-A receptor coding transcript had a lower expression in postmortem analysis of brains of patients who committed suicide (Yin et al., 2016). A significant increase in the expression of genes responsible for GABAergic synaptic transmission in postmortem analysis of the dorsolateral prefrontal cortex and anterior cingulate cortex of patients with MDD who died of suicide vs. MDD patients who died of other causes also supported the substantial role of GABAergic activity in patients with suicidal ideation (Zhao et al., 2018). TMS might interfere with this abnormality by causing an alteration in the GABA/glutamine ratio in corticolimbic circuits (Iwabuchi et al., 2017). Another spectroscopy study in humans showed an increased concentration of GABAergic activity after rTMS (Grohn et al., 2019). Serotonin has also been consistently proven to have a role in both depression and suicidality, and there is substantial evidence to support the role of rTMS in regulating abnormal serotonergic activity. A study by Baeken et al. (2011) showed that serotonergic dysregulation was restored after high-frequency rTMS in patients with unipolar depression (Baeken et al., 2011). A study by Kim et al. (2014) in a chronic stress model of depression in rats also supported the facilitation of GABAergic activity by rTMS (Kim et al., 2014).

### ECT Studies

Recent ECT studies consistently showed significant clinical effects of ECT on suicidality (Kellner et al., 2005; Bradvik and Berglund, 2006; Patel et al., 2006; Ambade et al., 2009; Keshtkar et al., 2011; Ahmadi et al., 2016; Kawoos et al., 2018; Liang et al., 2018), whereas earlier studies did not show any difference between ECT-treated groups and control patients (Avery and Winokur, 1976, 1978; Tsuang et al., 1979; Black et al., 1989; Sharma, 1999). This difference may be the result of standardization of ECT techniques, which allowed for retained clinical efficacy and fewer adverse effects (Tirmizi et al., 2012). Another explanation might be the increase in numbers of prospective and controlled studies. These higher quality studies may have provided more accurate results. Similarly studies in which suicidality was a primary outcome often showed improvement in suicidality. There were 11 studies which suicidality was the primary outcome. Seven of these showed an improvement in suicidal ideation or suicide intent and fewer suicide attempts or completed suicides in patients treated with ECT (Avery and Winokur, 1978; Rich et al., 1986; Black et al., 1987; Ciapparelli et al., 2001; Kellner et al., 2005; Bradvik and Berglund, 2006; Patel et al., 2006; Ahmadi et al., 2016; Kawoos et al., 2018). Two of them could not show any significant difference (Avery and Winokur, 1976; Black et al., 1989), while two of them showed a higher rates of completed suicide in the patients treated with ECT (Munk-Olsen et al., 2007; Jorgensen et al., 2020). Two of all the studies mentioned an association of improvement in suicidal ideation and intent with the improvement in depression (Kellner et al., 2005; Patel et al., 2006). Yet it is hard to reach a conclusion regarding if improvements in suicidal ideation or intent was barely a result of improvement in depression.

Although the mechanism of action is still unclear, multiple theories may explain how ECT contributes to improvement in suicidality. ECT may exert its effect on suicide indirectly by improving symptoms of depression and directly by improving suicidality. Treatment with ECT increases neurotrophic factors, such as brain-derived neurotrophic factor in hippocampal neurons, although data are not clear regarding whether depression improves with increasing levels of brain-derived neurotrophic factor (Gedge et al., 2012; Rapinesi et al., 2015). Normalizing the hypothalamic-pituitary-adrenal axis hyperfunction after ECT treatment in patients with depression and ensuing decreased cortisol levels in response to ECT could also improve suicidality (Singh and Kar, 2017). Treatment with ECT may also normalize GABA/glutamate levels, which have been reported as higher in the prefrontal cortex and hippocampus in rodent models of depression (Sartorius et al., 2007; Dong et al., 2010). Correcting altered glutamatergic activity in various areas of the brain in patients with depression (Michael et al., 2003) underscores the potential role of the glutamatergic system in efficacy of ECT. Improved plasticity and alterations in the functional connectivity of various networks are other possible mechanisms through which ECT, which exerts a positive effect on depression, might contribute to improvement in suicidality (Singh and Kar, 2017).

ECT may also directly address the mechanistic neurobiology of suicide. An increase in noradrenergic activity could explain its anti-suicidal activity. Postmortem analyses of brains of patients who committed suicide showed an increase in α_2_-noradrenergic binding, and ECT decreased this binding, implying an increase in noradrenergic hormone levels (Lillethorup et al., 2015). Another suggested mechanism of action is recovery of neuropeptide Y levels, which are lower in suicidal patients with depression (Ozsoy et al., 2016).

The ECT studies had limitations. Information regarding the difference in illness severity between patients who underwent ECT and control patients was not available for most studies we reviewed. Control patients were also poorly characterized in most studies, and the definition of control groups was not clear, making the comparisons vague. For studies with defined control groups, patients were treated with psychopharmacotherapy. One of the studies compared the effect of ECT with that of rTMS in patients with MDD (Keshtkar et al., 2011), which is a difficult comparison given the use of anesthesia with ECT and other related confounding issues. The number of ECT courses and the ECT parameters also varied across studies, making it challenging to synthesize information and draw definitive conclusions.

### MST Studies

The effect of MST on suicidality was described in two papers. In a study by Sun et al. (2018) which included 23 patients with TRD treated with MST, a decrease in long-interval cortical inhibition correlated with improved suicidal ideation, as measured by the SSI, although the mechanism of action was unclear. A decrease in long-interval cortical inhibition is thought to be linked to increased plasticity, and the study authors concluded that MST might exert its effects on suicidality through increased plasticity.

### VNS Studies

The effects of VNS were examined in eight studies. A study by Aaronson et al. (2017) showed that active VNS was associated with a significant decrease in suicidal ideation as measured by the QIDS-SR item 10 and also in suicidal behavior (gesture and attempt) as measured by an investigator-completed suicidality assessment (Aaronson et al., 2017). Another study by Aaronson et al. (2013) showed that suicide attempts were significantly higher among patients who were treated with low VNS dosing parameters vs. high dosing parameters, namely VNS current amplitude and pulse width (Aaronson et al., 2013). Bajbouj et al. (2010) showed a 3% suicide rate during 24 months of follow-up, which was a higher rate than expected in this patient population (Bajbouj et al., 2010). One of the studies did not show any significant difference between a treatment-as-usual plus VNS group and a treatment-as-usual group regarding suicidality, although it showed significant improvement in the treatment-as-usual plus VNS group in the marginal structural model (Olin et al., 2012). This same study also showed that an adjunctive VNS treatment group had a suicide rate that was nearly half that of the treatment-as-usual group, although the difference was not significant. Other studies reported attempted suicide rates lower than the usual rates in TRD (Nahas et al., 2005; Rush et al., 2005b; Schlaepfer et al., 2008). The attempted suicide rate was ~5% in studies by Rush et al. (2005a,b) and Nahas et al. (2005). Although studies are limited and not conclusive, they suggest that VNS may be effective in improving suicidality.

We can speculate about the possible mechanisms involved in how VNS affects suicidality. VNS has been shown to increase the levels of noradrenalin and serotonin in several parts of the rat brain (Roosevelt et al., 2006; Follesa et al., 2007; Conway and Xiong, 2018). The positive effect of VNS on the dopaminergic system has also been shown by increased ventral tegmental area activity in VNS responders (Aaronson et al., 2013), which is consistent with increased posttreatment dopamine metabolite homovanillic acid (Carpenter et al., 2004). Preclinical studies also showed that VNS has a positive effect on neural plasticity (Revesz et al., 2008; Biggio et al., 2009; Gebhardt et al., 2013); however, the relationship between this positive effect and the antidepressant effect of VNS is unclear. An indirect link might exist between these two hypotheses, namely increased neurotransmitters and plasticity, such that increased plasticity in the hippocampus may be the result of increased monoamines as monoaminergic projections to the hippocampus from the above-mentioned areas (Conway and Xiong, 2018).

### DBS Studies

Existing literature about DBS focuses on patients with Parkinson disease and patients with TRD, and both of these populations are important to consider in synthesizing and interpreting the DBS literature. Suicidality was not a primary outcome in any of these studies. Reaching definitive conclusions regarding the effect of DBS in suicidal ideation, suicide attempts, and completed suicide of patients with Parkinson disease is difficult because most studies did not evaluate suicidal outcomes preoperatively and postoperatively. Of the two studies that assessed before and after measures of suicidality (Castelli et al., 2006; Porat et al., 2009), one did not show a significant change in suicidal ideation (Castelli et al., 2006), and the other reported an increase (Porat et al., 2009) in the number of patients with suicidal ideation after surgery. The controlled study by Weintraub et al. (2013) was the only study that did not show any significant difference between DBS treatment and best medical therapy regarding suicidal ideation after surgery. Proxy symptoms of suicidality also improved in the DBS group compared with the best medical therapy group (Weintraub et al., 2013). Therefore, to determine if DBS has any association with suicidal ideation, it is important to compare the base rates of suicidal ideation in patients with Parkinson disease with the suicidal ideation rate in patients who received DBS.

Unfortunately, data regarding suicide rates in patients with Parkinson disease is not definitive. A recent systematic review by Berardelli et al. (2019) reported that some but not all studies showed increased suicide rates and suicidal ideation among patients than in the general population; however, it was difficult for the authors to reach a definitive conclusion regarding the prevalence of suicidality in patients with Parkinson disease (Berardelli et al., 2019). A retrospective study of ~150,000 patients with Parkinson disease showed a lower rate of suicide (0.08%) than the rate in the general population (0.8%) (Myslobodsky et al., 2001). However, other studies showed a higher risk of suicide in patients with Parkinson disease than in the general population (Shepard et al., 2019), which made estimating the real rates of suicide difficult. Therefore, it is hard to speculate regarding the effect of DBS treatment on suicidality for patients with Parkinson disease.

Previous data regarding the effect of DBS on suicidality for patients with Parkinson disease is not definitive. The largest cohort study showed a significant increase in suicidality (Voon et al., 2008), and other retrospective and prospective cohort studies (Berney et al., 2002; Lhommee et al., 2012; Chopra et al., 2014) reported higher suicide rates for patients with Parkinson disease who were treated with DBS than that reported in the cohort study mentioned above (Myslobodsky et al., 2001) with ~150,000 patients. Additionally, suicidal ideation, suicide attempt, and suicide completion after DBS were not specific for Parkinson disease. A meta-analysis by Appleby et al. (2007) showed that patients with other types of movement disorders including essential tremor and dystonia also had higher suicide rates. This may indicate a possible role for DBS in suicidality, regardless of the treated disorders. Several other studies also described mood and cognitive effects of DBS and the subsequent tendency for suicide (Rodriguez et al., 1998; Kumar et al., 1999; Moro et al., 1999).

#### Mechanisms Underlying Suicide Risk

Preliminary speculation about the underlying mechanism of suicide risk could be useful for future studies. There are four explanations for a higher suicide risk for patients who underwent DBS treatment. First, medical and neurologic complications after DBS surgery may have a role in suicidality. In a randomized controlled trial by Weintraub et al. (2013) suicide occurred in cases of patients with protracted medical complications after surgery. Second, pre-surgical psychiatric diagnoses and symptoms may be contributing factors. For example, in the randomized controlled trial by Weintraub et al. (2013) patients who completed suicide after surgery already had depression, based on BDI scores, before surgery. A recent report showed that 20% of Parkinson disease patients had apathy (Rektorova, 2019). A study by Pinsker et al. also showed significant preoperative psychiatric comorbidity (Pinsker et al., 2013). A history of psychiatric disorder and suicide are important to the appearance of suicidal ideation postoperatively, and nearly all studies that investigated this relationship reported a history of suicide attempts or a significant relationship between pre-surgical depression and postsurgical suicidality (Albanese et al., 2005). This association between pre-surgical psychiatric disorder and postoperative suicidal ideation is supported by other studies described in our review, including a study that reported pre-surgical suicide attempts (Bergfeld et al., 2016) or a history of severe psychiatric disorders (Albanese et al., 2005) for patients who committed suicide after surgery.

The effects of DBS are the third explanation for a possible underlying mechanism of postoperative psychiatric symptoms. A review by Temel et al. reported that 41% of patients had cognitive problems during follow-up, 8% experienced depression, and 4% had hypomania (Temel et al., 2009). The review also described apathy and depression after surgery, lasting for years (Cyron, 2016). A preclinical study by Temel et al. showed that subthalamic nucleus stimulation resulted in region and neuron-specific inhibition of serotonergic neurons of dorsal raphe nucleus in midbrain. Stimulation-induced depressive behavior in rodent models, reversed by selective serotonin reuptake inhibitors, also supports the serotonergic hypothesis (Temel et al., 2007). A possible mechanism of this inhibition may be related to connections between the subthalamic nucleus, the substantia nigra pars reticulate, and the lateral habenular nucleus, which receives input from the subthalamic nucleus and has substantial output to dorsal raphe (Smith et al., 1997). As discussed in the paper by Hershey et al. that described the effect of subthalamic nucleus on cognitive effects, connections between the subthalamic nucleus and frontal cortex might also explain the psychiatric adverse effects. They showed that stimulation of the subthalamic nucleus resulted in decreased working memory performance and decreased inhibitory control (Hershey et al., 2004) both of which have been shown to affect suicidal behavior (Burton et al., 2011; Richard-Devantoy et al., 2015), especially when the memory load and need for inhibitory control was higher. Both direct-afferent connections from the prefrontal cortex to the subthalamic nucleus and indirect-efferent connections to the dorsolateral prefrontal cortex and the anterior cingulate cortex through the substantia nigra pars reticulate of subthalamic nucleus to the frontal areas are thought to be responsible (Middleton and Strick, 1994). By contrast, Weintraub et al. showed in their randomized controlled trial that stimulation of the GPi might not increase and may even decrease suicidality (Weintraub et al., 2013).

Fourth, the postoperative appearance of psychiatric symptoms may result from discontinuation of medications for Parkinson disease. As Weintraub discussed, discontinuation of Parkinson medications is more common with STN surgery, which might explain the worse behavioral outcomes after surgery, especially when significant motor improvement allows medications to be reduced (Krack et al., 1998; Czernecki et al., 2008; Thobois et al., 2010). In the large retrospective cohort study by Voon et al. (2008) the suicide rate was 0.9% for patients who had STN surgery, whereas in the prospective cohort study by Lhommee et al. in which Parkinson disease medications were suddenly discontinued, the suicide rate was 3% (Lhommee et al., 2012). In the study by Voon et al., decreasing the levodopa equivalent daily dose score was possibly associated (*p* < 0.05) with suicide attempts (Voon et al., 2008). The dopamine agonist withdrawal syndrome is the recollection of physical and psychological symptoms that occurs when the dose of drugs to treat Parkinson disease is discontinued or decreased, especially dopaminergic agonists like levodopa (Yu and Fernandez, 2017). Psychological symptoms might include anxiety, panic attacks, depression, irritability, and agitation—all of which might be risk factors for suicidality, and DBS has been shown to significantly increase the risk of this syndrome (Yu and Fernandez, 2017). On the milder part of the spectrum, dysphoria and depression are common symptoms during dopaminergic agent withdrawal, which disappears after even a small dose of levodopa (Lilleeng and Dietrichs, 2008).

#### DBS Parameters and Suicidality

As mentioned in the report by Lhommee et al., DBS might improve well-being, euphoria, and apathy, whereas chronic DBS stimulation may result in increased apathy (Lhommee et al., 2012). Thus, a temporal relationship between DBS surgery and suicidality was also described in this study. However, we could not reach a significant conclusion from our review regarding temporal relationship because the time between DBS and suicidal ideation and behavior varied substantially. Because DBS parameters were not available in the reviewed studies, it was difficult to extrapolate any conclusions regarding the effect of various parameters on suicidality, including the location of the DBS implant.

In summary, many studies in the present review showed an increased risk of suicidality after DBS treatment in patients with Parkinson disease compared with patients' baseline risk of suicidality. A meta-analysis by Appleby et al. also supported these results (Appleby et al., 2007). However, the randomized control trial by Weintraub et al. did not report similar findings, therefore it was difficult to make conclusions (Weintraub et al., 2013).

DBS has also been used therapeutically for other movement disorders, including essential tremor and dystonia. The meta-analysis by Appleby et al. showed a suicidality rate of 0.3 to 0.7% in this patient population, whereas the suicide rate was 0.16 to 0.32% in the group that included patients with Parkinson disease, essential tremor, and dystonia (Appleby et al., 2007). Because of the mixed patient population, interpreting these results is difficult.

### DBS for TRD

DBS has also been used therapeutically for TRD. In the reports we reviewed, the highest completed suicide rate for this patient population was 29% (2/7), whereas the lowest rate was nearly 5% (1/21) (Lozano et al., 2012; Raymaekers et al., 2017). The rest of the studies reviewed presented data regarding suicide attempts and suicidal ideation. For completed suicide, a review by Rihmer showed a 14% completed suicide rate for patients who had DBS for TRD (Rihmer, 2007). Even though, most studies we reviewed reported lower completed suicide rates, this should be interpreted cautiously given the small sample sizes and lack of control groups in some of these studies. Several mechanisms were suggested to explain the improvement in suicidality after DBS. These include decrease in the hyperactivity in orbitofrontal cortex and medial thalamic regions might explain the improvement in suicidality via improving depression. In addition regaining the imbalance of noradrenergic and serotonergic activity in the prefrontal cortex might directly explain the improvement in suicidality as increased noradrenergic receptor density was shown in patients with depression who completed suicide (Velasco et al., 2005).

### Summary

We have presented an extensive summary of the literature regarding the effects of neuromodulation studies on suicidality. We believe this review is especially important for increasing understanding of the role of DBS, as there have been ongoing concerns regarding its behavioral effects. We followed PRISMA guidelines and performed a thorough search. There were no deviations from the previously registered protocol in PROSPERO.

### Limitations

This systematic review has substantial limitations. Few of the prior studies of brain stimulation were designed or adequately powered to assess outcomes related to suicide. Most of the studies did not present data regarding change in suicidality before and after surgery and presented data only as numbers of patients, which made it difficult to make any definite conclusions. In addition, most studies were retrospective and were subject to the inherent biases of that study design, including recall and misclassification bias. Heterogeneity in patient populations, especially in earlier studies, treatment parameters, and study duration also limited this review. Because of these weaknesses, future prospective studies are needed that evaluate suicidality as a main outcome. Possible markers of suicidality must be determined to help prevent the high prevalence of suicide, which is a major public health problem. Further study of the distinct neurobiological aspects of suicidal behaviors, suicidal ideation, suicide attempts, and completed suicide is critical for understanding and refining the broad clinical concept of suicidality.

## Conclusion

To our knowledge, this is the first systematic review of neuromodulation interventions for suicidal ideation. On the basis of available information, TMS, ECT, and MST are promising therapeutic tools to directly address suicidal ideation in the context of mood disorders. Initial naturalistic studies with VNS are also promising. Most DBS studies focused on Parkinson disease, and results were mixed. Currently, it is unknown whether neuromodulation interventions would directly address suicidality regardless of diagnosis or affective state (e.g., patients who do not meet diagnostic criteria for a mood disorder). Biomarkers to index the severity of suicidality, assist in treatment planning, and monitor the effects of neurostimulation would help in development of neuromodulation interventions for suicidality. Further, prospective studies could help to develop new therapeutic interventions to address this global challenge and major public health issue.

## Data Availability Statement

The original contributions presented in the study are included in the article/Supplementary Material, further inquiries can be directed to the corresponding author/s.

## Author Contributions

PC, CL, and MK conceptualized the systematic review. MK, AA, AS, AL, DD, CL, and PC developed and consulted on the search strategy and methodology. MK, AA, AS, AL, CL, and PC assisted with screening articles. MK and AA abstracted data from the articles. MK, AA, and PC drafted the manuscript. All authors reviewed, edited, assisted with writing subsequent drafts of the manuscript, and approved the final version of the manuscript.

## Conflict of Interest

CL receives grant support from the Brain & Behavior Research Foundation as the Alan G. Ross Memorial Investigator. He has been a site investigator for multicenter trials funded by Neuronetics, Inc. and NeoSync, Inc. PC has received research grant support from the National Institute of Mental Health, Brain & Behavior Research Foundation, Neuronetics, Inc., and Pfizer, Inc. He has received equipment support from Neuronetics Inc. and MagVenture, Inc. He has received supplies and genotyping services from Assurex Health, Inc. for investigator-initiated studies. He was the primary investigator for a multicenter study funded by Neuronetics, Inc. and a site primary investigator for a study funded by NeoSync, Inc. He has served as a paid consultant for Engrail Therapeutics, Myriad Neuroscience, Procter & Gamble Company, and Sunovion. The remaining authors declare that the research was conducted in the absence of any commercial or financial relationships that could be construed as a potential conflict of interest.

## Abbreviations

BDI: Beck Depression Inventory
BPRS-24: Brief Psychiatric Rating Scale-24
BSI: Beck Suicidal Intent Scale
BSS: Beck Scale for Suicidal Ideation
CDRS-R: Children's Depression Rating Scale–Revised
CEA: cortical-evoked activity
C-SSRS: Columbia Suicide Severity Rating Scale
DBS: deep brain stimulation
*df*: degrees of freedom
ECT: electroconvulsive therapy
FDA: US Food and Drug Administration
GABA: γ-aminobutyric acid
GPi: globus pallidus interna
HDRS: Hamilton Depression Rating Scale
iTBS: intermittent theta-burst stimulation
LICI: long-interval intracortical inhibition
MADRS: Montgomery-Asberg Depression Rating Scale
MDD: major depressive disorder
MINI: Mini International Neuropsychiatric Interview
MST: magnetic seizure therapy
OCD: obsessive-compulsive disorder
OR: odds ratio
PDQ-39: Parkinson Disease Questionnaire, 39 item
PRIDE: Population Research in Identities and Disparities for Equality
PRISMA: Preferred Reporting Items for Systematic Reviews and Meta-Analyses
PTSD: posttraumatic stress disorder
QIDS-SR-16: Quick Inventory of Depressive Symptomatology–Self-Report 16
RR: relative risk
rTMS: repetitive TMS
SELECT TDCS: Sertraline vs. Electrical Current Therapy for Treating Depression Clinical Study
SF-36: Short Form (36) Health Survey
SIQ: Suicidal Ideation Questionnaire
SIS: suicide ideation scale
SSI: Scale for Suicidal Ideation
STN: subthalamic nucleus
TBS: theta-burst stimulation
tDCS: transcranial direct-current stimulation
TMS: transcranial magnetic stimulation
TRD: treatment-resistant depression
VNS: vagal nerve stimulation.

## References

[B1] Aaronson S. T. Carpenter L. L. Conway C. R. Reimherr F. W. Lisanby S. H. Schwartz T. L.. (2013). Vagus nerve stimulation therapy randomized to different amounts of electrical charge for treatment-resistant depression: acute and chronic effects. Brain Stimul. 6, 631–640. 10.1016/j.brs.2012.09.013 23122916

[B2] Aaronson S. T. Sears P. Ruvuna F. Bunker M. Conway C. R. Dougherty D. D.. (2017). A 5-year observational study of patients with treatment-resistant depression treated with vagus nerve stimulation or treatment as usual: comparison of response, remission, and suicidality. Am. J. Psychiatry 174, 640–648. 10.1176/appi.ajp.2017.16010034 28359201

[B3] Abdelnaim M. A. Langguth B. Deppe M. Mohonko A. Kreuzer P. M. Poeppl T. B.. (2019). Anti-suicidal efficacy of repetitive transcranial magnetic stimulation in depressive patients: a retrospective analysis of a large sample. Front. Psychiatry 10:929. 10.3389/fpsyt.2019.00929 31969842 PMC6960193

[B4] Ahmadi N. Moss L. Hauser P. Nemeroff C. Atre-Vaidya N. (2018). Clinical outcome of maintenance electroconvulsive therapy in comorbid posttraumatic stress disorder and major depressive disorder. J. Psychiatr. Res. 105, 132–136. 10.1016/j.jpsychires.2018.08.023 30219562

[B5] Ahmadi N. Moss L. Simon E. Nemeroff C. B. Atre-Vaidya N. (2016). Efficacy and long-term clinical outcome of comorbid posttraumatic stress disorder and major depressive disorder after electroconvulsive therapy. Depress. Anxiety 33, 640–647. 10.1002/da.22451 26555786

[B6] Albanese A. Piacentini S. Romito L. M. Leone M. Franzini A. Broggi G.. (2005). Suicide after successful deep brain stimulation for movement disorders. Neurology 65, 499–500. 10.1212/WNL.65.3.499 16087934

[B7] Ambade V. Arora M. M. Singh P. Somani B. L. Basannar D. (2009). Adrenaline, noradrenaline and dopamine level estimation in depression: does it help? Med. J. Armed Forces India 65, 216–220. 10.1016/S0377-1237(09)80006-3 27408249 PMC4921403

[B8] Antonini A. (2007). Continuous dopaminergic stimulation: from theory to clinical practice. Parkinsonism Relat. Disord. 13, S24–S28. 10.1016/j.parkreldis.2007.06.002 17702632

[B9] Appleby B. S. Duggan P. S. Regenberg A. Rabins P. V. (2007). Psychiatric and neuropsychiatric adverse events associated with deep brain stimulation: a meta-analysis of ten years' experience. Mov. Disord. 22, 1722–1728. 10.1002/mds.21551 17721929

[B10] Avery D. Winokur G. (1976). Mortality in depressed patients treated with electroconvulsive therapy and antidepressants. Arch. Gen. Psychiatry 33, 1029–1037. 10.1001/archpsyc.1976.01770090019001 962487

[B11] Avery D. Winokur G. (1978). Suicide, attempted suicide, and relapse rates in depression. Arch. Gen. Psychiatry 35, 749–753. 10.1001/archpsyc.1978.01770300091010 655772

[B12] Baeken C. De Raedt R. Bossuyt A. Van Hove C. Mertens J. Dobbeleir A.. (2011). The impact of HF-rTMS treatment on serotonin(2A) receptors in unipolar melancholic depression. Brain Stimul. 4, 104–111. 10.1016/j.brs.2010.09.002 21511211

[B13] Baeken C. Duprat R. Wu G. R. De Raedt R. van Heeringen K. (2017). Subgenual anterior cingulate-medialo orbitofrontal functional Connectivity in medication-resistant major depression: a neurobiological marker for accelerated intermittent theta burst stimulation treatment? Biol. Psychiatry Cogn. Neurosci. Neuroimaging 2, 556–565. 10.1016/j.bpsc.2017.01.001 29560909

[B14] Baeken C. Wu G. R. van Heeringen K. (2019). Placebo aiTBS attenuates suicidal ideation and frontopolar cortical perfusion in major depression. Transl. Psychiatry 9:38. 10.1038/s41398-019-0377-x 30696807 PMC6351528

[B15] Bajbouj M. Merkl A. Schlaepfer T. E. Frick C. Zobel A. Maier W.. (2010). Two-year outcome of vagus nerve stimulation in treatment-resistant depression. J. Clin. Psychopharmacol. 30, 273–281. 10.1097/JCP.0b013e3181db8831 20473062

[B16] Beck A. T. Kovacs M. Weissman A. (1979). Assessment of suicidal intention: the Scale for Suicide Ideation. J. Consult. Clin. Psychol. 47, 343–352. 10.1037/0022-006X.47.2.343 469082

[B17] Benzoni O. Fazzari G. Marangoni C. Placentino A. Rossi A. (2015). Treatment of resistant mood and schizoaffective disorders with electroconvulsive therapy: a case series of 264 patients. J. Psychopathol. 21, 266–268. Available online at: https://www.jpsychopathol.it/article/treatment-of-resistant-mood-and-schizoaffective-disorders-with-electroconvulsive-therapy-a-case-series-of-264-patients/

[B18] Berardelli I. Belvisi D. Nardella A. Falcone G. Lamis D. A. Fabbrini G.. (2019). Suicide in Parkinson's disease: a systematic review. CNS Neurol. Disord. Drug Targets 18, 466–477. 10.2174/1871527318666190703093345 31269887

[B19] Bergfeld I. O. Mantione M. Hoogendoorn M. L. Ruhe H. G. Notten P. van Laarhoven J.. (2016). Deep brain stimulation of the ventral anterior limb of the internal capsule for treatment-resistant depression: a randomized clinical trial. JAMA Psychiatry 73, 456–464. 10.1001/jamapsychiatry.2016.0152 27049915

[B20] Berlim M. T. Van den Eynde F. Tovar-Perdomo S. Chachamovich E. Zangen A. Turecki G. (2014). Augmenting antidepressants with deep transcranial magnetic stimulation (DTMS) in treatment-resistant major depression. World J. Biol. Psychiatry 15, 570–578. 10.3109/15622975.2014.925141 25050453

[B21] Berney A. Vingerhoets F. Perrin A. Guex P. Villemure J. G. Burkhard P. R.. (2002). Effect on mood of subthalamic DBS for Parkinson's disease: a consecutive series of 24 patients. Neurology 59, 1427–1429. 10.1212/01.WNL.0000032756.14298.18 12427897

[B22] Bewernick B. H. Kayser S. Sturm V. Schlaepfer T. E. (2012). Long-term effects of nucleus accumbens deep brain stimulation in treatment-resistant depression: evidence for sustained efficacy. Neuropsychopharmacology 37, 1975–1985. 10.1038/npp.2012.44 22473055 PMC3398749

[B23] Biggio F. Gorini G. Utzeri C. Olla P. Marrosu F. Mocchetti I.. (2009). Chronic vagus nerve stimulation induces neuronal plasticity in the rat hippocampus. Int. J. Neuropsychopharmacol. 12, 1209–1221. 10.1017/S1461145709000200 19309534 PMC2879889

[B24] Black D. W. Winokur G. Mohandoss E. Woolson R. F. Nasrallah A. (1989). Does treatment influence mortality in depressives? A follow-up of 1076 patients with major affective disorders. Ann. Clin. Psychiatry 1, 165–173. 10.3109/10401238909149975

[B25] Black D. W. Winokur G. Nasrallah A. (1987). The treatment of depression: electroconvulsive therapy v antidepressants: a naturalistic evaluation of 1,495 patients. Compr. Psychiatry 28, 169–182. 10.1016/0010-440X(87)90082-4 3829660

[B26] Bloch Y. Grisaru N. Harel E. V. Beitler G. Faivel N. Ratzoni G.. (2008). Repetitive transcranial magnetic stimulation in the treatment of depression in adolescents: an open-label study. J. ECT 24, 156–159. 10.1097/YCT.0b013e318156aa49 18580562

[B27] Blumberger D. M. Maller J. J. Thomson L. Mulsant B. H. Rajji T. K. Maher M.. (2016). Unilateral and bilateral MRI-targeted repetitive transcranial magnetic stimulation for treatment-resistant depression: a randomized controlled study. J. Psychiatry Neurosci. 41, E58–E66. 10.1503/jpn.150265 27269205 PMC4915938

[B28] Borretzen M. N. Bjerknes S. Saehle T. Skjelland M. Skogseid I. M. Toft M.. (2014). Long-term follow-up of thalamic deep brain stimulation for essential tremor: patient satisfaction and mortality. BMC Neurol. 14:120. 10.1186/1471-2377-14-120 24903550 PMC4052337

[B29] Bradvik L. Berglund M. (2000). Treatment and suicide in severe depression: a case-control study of antidepressant therapy at last contact before suicide. J. ECT 16, 399–408. 10.1097/00124509-200012000-00010 11314878

[B30] Bradvik L. Berglund M. (2006). Long-term treatment and suicidal behavior in severe depression: ECT and antidepressant pharmacotherapy may have different effects on the occurrence and seriousness of suicide attempts. Depress. Anxiety 23, 34–41. 10.1002/da.20134 16315268

[B31] Brunoni A. R. Chaimani A. Moffa A. H. Razza L. B. Gattaz W. F. Daskalakis Z. J.. (2017). Repetitive transcranial magnetic stimulation for the acute treatment of major depressive episodes: a systematic review with network meta-analysis. JAMA Psychiatry 74, 143–152. 10.1001/jamapsychiatry.2016.3644 28030740

[B32] Brunoni A. R. Junior R. F. Kemp A. H. Lotufo P. A. Bensenor I. M. Fregni F. (2014). Differential improvement in depressive symptoms for tDCS alone and combined with pharmacotherapy: an exploratory analysis from the Sertraline vs. electrical current therapy for treating depression clinical study. Int. J. Neuropsychopharmacol. 17, 53–61. 10.1017/S1461145713001065 24060107

[B33] Brunoni A. R. Valiengo L. Baccaro A. Zanão T. A. de Oliveira J. F Goulart A.. (2013). The sertraline vs. electrical current therapy for treating depression clinical study from a factorial, randomized, controlled trial. JAMA Psychiatry 70, 383–391. 10.1001/2013.jamapsychiatry.32 23389323

[B34] Burkhard P. R. Vingerhoets F. J. Berney A. Bogousslavsky J. Villemure J. G. Ghika J. (2004). Suicide after successful deep brain stimulation for movement disorders. Neurology 63, 2170–2172. 10.1212/01.WNL.0000145603.48221.B5 15596774

[B35] Burton C. Z. Vella L. Weller J. A. Twamley E. W. (2011). Differential effects of executive functioning on suicide attempts. J. Neuropsychiatry Clin. Neurosci. 23, 173–179. 10.1176/jnp.23.2.jnp173 21677246 PMC3626287

[B36] Cakir S. Caglar N. (2017). Electroconvulsive therapy in the treatment of mood disorders: one-year follow-up. Noro Psikiyatr. Ars. 54, 196–201. 10.5152/npa.2016.14845 29033629 PMC5630095

[B37] Carpenter L. L. Aaronson S. T. Clarke G. N. Holtzheimer P. E. Johnson C. W. McDonald W. M.. (2017). rTMS with a two-coil array: safety and efficacy for treatment resistant major depressive disorder. Brain Stimul. 10, 926–933. 10.1016/j.brs.2017.06.003 28642024

[B38] Carpenter L. L. Moreno F. A. Kling M. A. Anderson G. M. Regenold W. T. Labiner D. M.. (2004). Effect of vagus nerve stimulation on cerebrospinal fluid monoamine metabolites, norepinephrine, and gamma-aminobutyric acid concentrations in depressed patients. Biol. Psychiatry 56, 418–426. 10.1016/j.biopsych.2004.06.025 15364040

[B39] Castelli L. Perozzo P. Zibetti M. Crivelli B. Morabito U. Lanotte M.. (2006). Chronic deep brain stimulation of the subthalamic nucleus for Parkinson's disease: effects on cognition, mood, anxiety and personality traits. Eur. Neurol. 55, 136–144. 10.1159/000093213 16682797

[B40] Chervyakov A. V. Chernyavsky A. Y. Sinitsyn D. O. Piradov M. A. (2015). Possible mechanisms underlying the therapeutic effects of transcranial magnetic stimulation. Front. Hum. Neurosci. 9:303. 10.3389/fnhum.2015.00303 26136672 PMC4468834

[B41] Chopra A. Abulseoud O. A. Sampson S. Lee K. H. Klassen B. T. Fields J. A.. (2014). Mood stability in Parkinson disease following deep brain stimulation: a 6-month prospective follow-up study. Psychosomatics 55, 478–484. 10.1016/j.psym.2013.09.003 24360528 PMC4063889

[B42] Ciapparelli A. Dell'Osso L. Tundo A. Pini S. Chiavacci M. C. Di Sacco I.. (2001). Electroconvulsive therapy in medication-nonresponsive patients with mixed mania and bipolar depression. J. Clin. Psychiatry 62, 552–555. 10.4088/JCP.v62n07a09 11488367

[B43] Conway C. R. Xiong W. (2018). The mechanism of action of vagus nerve stimulation in treatment-resistant depression: current conceptualizations. Psychiatr. Clin. North. Am. 41, 395–407. 10.1016/j.psc.2018.04.005 30098653

[B44] Croarkin P. E. Nakonezny P. A. Deng Z. D. Romanowicz M. Voort J. L. V. Camsari D. D.. (2018). High-frequency repetitive TMS for suicidal ideation in adolescents with depression. J. Affect. Disord. 239, 282–290. 10.1016/j.jad.2018.06.048 30031247 PMC6431788

[B45] Crowell A. L. Riva-Posse P. Holtzheimer P. E. Garlow S. J. Kelley M. E. Gross R. E.. (2019). Long-term outcomes of subcallosal cingulate deep brain stimulation for treatment-resistant depression. Am. J. Psychiatry 176, 949–956. 10.1176/appi.ajp.2019.18121427 31581800

[B46] Cyron D. (2016). Mental side effects of deep brain stimulation (DBS) for movement disorders: the futility of denial. Front. Integr. Neurosci. 10:17. 10.3389/fnint.2016.00017 27147988 PMC4837159

[B47] Czernecki V. Schupbach M. Yaici S. Levy R. Bardinet E. Yelnik J.. (2008). Apathy following subthalamic stimulation in Parkinson disease: a dopamine responsive symptom. Mov. Disord. 23, 964–969. 10.1002/mds.21949 18398913

[B48] Desmyter S. Duprat R. Baeken C. Bijttebier S. van Heeringen K. (2014). The acute effects of accelerated repetitive transcranial magnetic stimulation on suicide risk in unipolar depression: preliminary results. Psychiatr. Danub. 26(Suppl. 1), 48–52. Available online at: https://europepmc.org/article/med/25413512 25413512

[B49] Desmyter S. Duprat R. Baeken C. Van Autreve S. Audenaert K. van Heeringen K. (2016). Accelerated intermittent theta burst stimulation for suicide risk in therapy-resistant depressed patients: a randomized, sham-controlled trial. Front. Hum. Neurosci. 10:480. 10.3389/fnhum.2016.00480 27729854 PMC5037167

[B50] Desseilles M. Perroud N. Guillaume S. Jaussent I. Genty C. Malafosse A.. (2012). Is it valid to measure suicidal ideation by depression rating scales? J. Affect. Disord. 136, 398–404. 10.1016/j.jad.2011.11.013 22154567

[B51] Dong J. Min S. Wei K. Li P. Cao J. Li Y. (2010). Effects of electroconvulsive therapy and propofol on spatial memory and glutamatergic system in hippocampus of depressed rats. J. ECT 26, 126–130. 10.1097/YCT.0b013e3181a9947a 20514696

[B52] Dougherty D. A. Rezai A. R. Carpenter L. L. Howland R. H. Bhati M. T. O?Reardon J. P.. (2015). A randomized sham-controlled trial of deep brain stimulation of the ventral capsule/ventral striatum for chronic treatment-resistant depression. Biol. Psychiatry 78, 240–248. 10.1016/j.biopsych.2014.11.023 25726497

[B53] Dumas R. Padovani R. Richieri R. Lancon C. (2012). [Repetitive transcranial magnetic stimulation in major depression: response factor]. Encephale 38, 360–368. 10.1016/j.encep.2011.08.004 22980479

[B54] Fitzgerald P. B. Chen L. Richardson K. Daskalakis Z. J. Hoy K. E. (2020). A pilot investigation of an intensive theta burst stimulation protocol for patients with treatment resistant depression. Brain Stimul. 13, 137–144. 10.1016/j.brs.2019.08.013 31477542

[B55] Fitzgerald P. B. Hoy K. E. Elliot D. Susan McQueen R. N. Wambeek L. E. Daskalakis Z. J. (2018). Accelerated repetitive transcranial magnetic stimulation in the treatment of depression. Neuropsychopharmacology 43, 1565–1572. 10.1038/s41386-018-0009-9 29467437 PMC5983543

[B56] Follesa P. Biggio F. Gorini G. Caria S. Talani G. Dazzi L.. (2007). Vagus nerve stimulation increases norepinephrine concentration and the gene expression of BDNF and bFGF in the rat brain. Brain Res. 1179, 28–34. 10.1016/j.brainres.2007.08.045 17920573

[B57] Follett K. A. Weaver F. M. Stern M. Hur K. Harris C. L. Luo P.. (2010). Pallidal versus subthalamic deep-brain stimulation for Parkinson?s disease. N. Engl. J. Med. 362, 2077–2091. 10.1056/NEJMoa0907083 20519680

[B58] Foncke E. M. Schuurman P. R. Speelman J. D. (2006). Suicide after deep brain stimulation of the internal globus pallidus for dystonia. Neurology 66, 142–143. 10.1212/01.wnl.0000191328.05752.e2 16401868

[B59] Francis J. Dingley J. (2015). Electroanaesthesia: from torpedo fish to TENS. Anaesthesia 70, 93–103. 10.1111/anae.12887 25348076

[B60] Franklin J. C. Ribeiro J. D. Fox K. R. Bentley K. H. Kleiman E. M. Huang X.. (2017). Risk factors for suicidal thoughts and behaviors: a meta-analysis of 50 years of research. Psychol. Bull. 143, 187–232. 10.1037/bul0000084 27841450

[B61] Funkiewiez A. Ardouin C. Caputo E. Krack P. Fraix V. Klinger H.. (2004). Long term effects of bilateral subthalamic nucleus stimulation on cognitive function, mood, and behaviour in Parkinson's disease. J. Neurol. Neurosurg. Psychiatry 75, 834–839. 10.1136/jnnp.2002.009803 15145995 PMC1739075

[B62] Gardner J. (2013). A history of deep brain stimulation: technological innovation and the role of clinical assessment tools. Soc. Stud. Sci. 43, 707–728. 10.1177/0306312713483678

[B63] Gebhardt N. Bar K. J. Boettger M. K. Grecksch G. Keilhoff G. Reichart R.. (2013). Vagus nerve stimulation ameliorated deficits in one-way active avoidance learning and stimulated hippocampal neurogenesis in bulbectomized rats. Brain Stimul. 6, 78–83. 10.1016/j.brs.2012.01.009 22405742

[B64] Gedge L. Beaudoin A. Lazowski L. du Toit R. Jokic R. Milev R. (2012). Effects of electroconvulsive therapy and repetitive transcranial magnetic stimulation on serum brain-derived neurotrophic factor levels in patients with depression. Front. Psychiatry 3:12. 10.3389/fpsyt.2012.00012 22375129 PMC3285902

[B65] George M. S. Lisanby S. H. Avery D. McDonald W. M. Durkalski V. Pavlicova M.. (2010). Daily left prefrontal transcranial magnetic stimulation therapy for major depressive disorder: a sham-controlled randomized trial. Arch. Gen. Psychiatry 67, 507–516. 10.1001/archgenpsychiatry.2010.46 20439832

[B66] George M. S. Post R. M. (2011). Daily left prefrontal repetitive transcranial magnetic stimulation for acute treatment of medication-resistant depression. Am. J. Psychiatry 168, 356–364. 10.1176/appi.ajp.2010.10060864 21474597

[B67] George M. S. Raman R. Benedek D. M. Pelic C. G. Grammer G. G. Stokes K. T.. (2014). A two-site pilot randomized 3 day trial of high dose left prefrontal repetitive transcranial magnetic stimulation (rTMS) for suicidal inpatients. Brain. Stimul. 7, 421–431. 10.1016/j.brs.2014.03.006 24731434

[B68] Giannini G. Francois M. Lhommee E. Polosan M. Schmitt E. Fraix V.. (2019). Suicide and suicide attempts after subthalamic nucleus stimulation in Parkinson disease. Neurology 93, e97–e105. 10.1212/WNL.0000000000007665 31101738

[B69] Gilman S. L. (2008). Electrotherapy and mental illness: then and now. Hist. Psychiatry 19(Pt. 3), 339–357. 10.1177/0957154X07082566 20617636

[B70] Glenn C. R. Cha C. B. Kleiman E. M. Nock M. K. (2017). Understanding suicide risk within the Research Domain Criteria (RDoC) framework: insights, challenges, and future research considerations. Clin. Psychol. Sci. 5, 568–592. 10.1177/2167702616686854 28670505 PMC5487002

[B71] Grohn H. Gillick B. T. Tkac I. Bednarik P. Mascali D. Deelchand D. K.. (2019). Influence of repetitive transcranial magnetic stimulation on human neurochemistry and functional connectivity: a pilot MRI/MRS atudy at 7 T. Front. Neurosci. 13:1260. 10.3389/fnins.2019.01260 31827419 PMC6890551

[B72] Hadley D. Anderson B. S. Borckardt J. J. Arana A. Li X. Nahas Z.. (2011). Safety, tolerability, and effectiveness of high doses of adjunctive daily left prefrontal repetitive transcranial magnetic stimulation for treatment-resistant depression in a clinical setting. J. ECT 27, 18–25. 10.1097/YCT.0b013e3181ce1a8c 21343710

[B73] Hamilton M. (1960). A rating scale for depression. J. Neurol. Neurosurg. Psychiatry 23, 56–62. 10.1136/jnnp.23.1.56 14399272 PMC495331

[B74] Hays R. D. Morales L. S. (2001). The RAND-36 measure of health-related quality of life. Ann. Med. 33, 350–357. 10.3109/07853890109002089 11491194

[B75] Hershey T. Revilla F. J. Wernle A. Gibson P. S. Dowling J. L. Perlmutter J. S. (2004). Stimulation of STN impairs aspects of cognitive control in PD. Neurology 62, 1110–1114. 10.1212/01.WNL.0000118202.19098.10 15079009

[B76] Higgins J. P. T. Savoic J. Page M. J. Elbers R. G. Sterne J. A. C. (2019). “Chapter 8: Assessing risk of bias in a randomized tiral,” in Cochrane Handbook for Systematic Reviews of Interventions. Version 6.0, eds J. P. T. Higgins, J. Thomas, J. Chandler, M. Cumpston, T. Li, M. J. Page, et al. (London: Cochrane St. Albans), 205–228. 10.1002/9781119536604.ch8

[B77] Holtzheimer P. E. Kelley M. E. Gross R. E. Filkowski M. M. Garlow S. J. Barrocas A.. (2012). Subcallosal cingulate deep brain stimulation for treatment-resistant unipolar and bipolar depression. Arch. Gen. Psychiatry 69, 150–158. 10.1001/archgenpsychiatry.2011.1456 22213770 PMC4423545

[B78] Holtzheimer P. E. 3rd McDonald W. M. Mufti M. Kelley M. E. Quinn S. Corso G. Epstein C. M. (2010). Accelerated repetitive transcranial magnetic stimulation for treatment-resistant depression. Depress. Anxiety 27, 960–963. 10.1002/da.20731 20734360 PMC3020591

[B79] Houeto J. L. Mesnage V. Mallet L. Pillon B. Gargiulo M. du Moncel S. T.. (2002). Behavioural disorders, Parkinson's disease and subthalamic stimulation. J. Neurol. Neurosurg. Psychiatry 72, 701–707. 10.1136/jnnp.72.6.701 12023409 PMC1737905

[B80] Huff W. Lenartz D. Schormann M. Lee S. H. Kuhn J. Koulousakis A.. (2010). Unilateral deep brain stimulation of the nucleus accumbens in patients with treatment-resistant obsessive-compulsive disorder: Outcomes after one year. Clin. Neurol. Neurosurg. 112, 137–143. 10.1016/j.clineuro.2009.11.006 20006424

[B81] Hunt I. M. Windfuhr K. Swinson N. Shaw J. Appleby L. Kapur N. (2011). Electroconvulsive therapy and suicide among the mentally ill in England: a national clinical survey. Psychiatry Res. 187, 145–149. 10.1016/j.psychres.2010.12.014 21208662

[B82] Isometsa E. T. Henriksson M. M. Heikkinen M. E. Lonnqvist J. K. (1996). Completed suicide and recent electroconvulsive therapy in Finland. Convuls. Ther. 12, 152–155. 8872403

[B83] Iwabuchi S. J. Raschke F. Auer D. P. Liddle P. F. Lankappa S. T. Palaniyappan L. (2017). Targeted transcranial theta-burst stimulation alters fronto-insular network and prefrontal GABA. Neuroimage 146, 395–403. 10.1016/j.neuroimage.2016.09.043 27651067

[B84] Jacobs B. L. van Praag H. Gage F. H. (2000). Adult brain neurogenesis and psychiatry: a novel theory of depression. Mol. Psychiatry 5, 262–269. 10.1038/sj.mp.4000712 10889528

[B85] Jenkinson C. Fitzpatrick R. Peto V. Greenhall R. Hyman N. (1997). The Parkinson's Disease Questionnaire (PDQ-39): development and validation of a Parkinson's disease summary index score. Age. Ageing 26, 353–357. 10.1093/ageing/26.5.353 9351479

[B86] Jollant F. Lawrence N. L. Olie E. Guillaume S. Courtet P. (2011). The suicidal mind and brain: a review of neuropsychological and neuroimaging studies. World J. Biol. Psychiatry 12, 319–339. 10.3109/15622975.2011.556200 21385016

[B87] Jorgensen M. B. Rozing M. P. Kellner C. H. Osler M. (2020). Electroconvulsive therapy, depression severity and mortality: data from the Danish National Patient Registry. J. Psychopharmacol. 34, 273–279. 10.1177/0269881119895518 31909674

[B88] Just M. A. Pan L. Cherkassky V. L. McMakin D. L. Cha C. Nock M. K.. (2017). Machine learning of neural representations of suicide and emotion concepts identifies suicidal youth. Nat. Hum. Behav. 1, 911–919. 10.1038/s41562-017-0234-y 29367952 PMC5777614

[B89] Kawoos Y. Shah I. A. Rather Y. H. Wani Z. A. Zarger W. A. (2018). Efficacy of electroconvulsive therapy in various psychiatric disorders: a hospital based longitudinal follow-up study. J. Clin. Diagn. Res. 12, VC10–VC14. 10.7860/JCDR/2018/31410.11446

[B90] Kellner C. H. Fink M. Knapp R. Petrides G. Husain M. Rummans T.. (2005). Relief of expressed suicidal intent by ECT: a consortium for research in ECT study. Am. J. Psychiatry 162, 977–982. 10.1176/appi.ajp.162.5.977 15863801 PMC3684568

[B91] Kellner C. H. Husain M. M. Knapp R. G. McCall W. V. Petrides G. Rudorfer M. V.. (2016). A novel strategy for continuation ECT in geriatric depression:pPhase 2 of the PRIDE study. Am. J. Psychiatry 173, 1110–1118. 10.1176/appi.ajp.2016.16010118 27418381 PMC7130448

[B92] Kennedy S. H. Giacobbe P. Rizvi S. J. Placenza F. M. Nishikawa Y. Mayberg H. S.. (2011). Deep brain stimulation for treatment-resistant depression: follow-up after 3 to 6 years. Am. J. Psychiatry 168, 502–510. 10.1176/appi.ajp.2010.10081187 21285143

[B93] Kenney C. Simpson R. Hunter C. Ondo W. Almaguer M. Davidson A.. (2007). Short-term and long-term safety of deep brain stimulation in the treatment of movement disorders. J. Neurosurg. 106, 621–625. 10.3171/jns.2007.106.4.621 17432713

[B94] Keshtkar M. Ghanizadeh A. Firoozabadi A. (2011). Repetitive transcranial magnetic stimulation versus electroconvulsive therapy for the treatment of major depressive disorder, a randomized controlled clinical trial. J. ECT 27, 310–314. 10.1097/YCT.0b013e318221b31c 22080240

[B95] Kim S. Y. Lee D. W. Kim H. Bang E. Chae J. H. Choe B. Y. (2014). Chronic repetitive transcranial magnetic stimulation enhances GABAergic and cholinergic metabolism in chronic unpredictable mild stress rat model:H-NMR spectroscopy study at 11.7T. Neurosci. Lett. 572, 32–37. 10.1016/j.neulet.2014.04.033 24796814

[B96] Kleiner-Fisman G. Fisman D. N. Sime E. Saint-Cyr J. A. Lozano A. M. Lang A. E. (2003). Long-term follow up of bilateral deep brain stimulation of the subthalamic nucleus in patients with advanced Parkinson disease. J. Neurosurg. 99, 489–495. 10.3171/jns.2003.99.3.0489 12959435

[B97] Krack P. Batir A. Van Blercom N. Chabardes S. Fraix V. Ardouin C.. (2003). Five-year follow-up of bilateral stimulation of the subthalamic nucleus in advanced Parkinson's disease. N. Engl. J. Med. 349, 1925–1934. 10.1056/NEJMoa035275 14614167

[B98] Krack P. Pollak P. Limousin P. Hoffmann D. Xie J. Benazzouz A.. (1998). Subthalamic nucleus or internal pallidal stimulation in young onset Parkinson's disease. Brain 121(Pt. 3), 451–457. 10.1093/brain/121.3.451 9549521

[B99] Kumar R. Lozano A. M. Sime E. Halket E. Lang A. E. (1999). Comparative effects of unilateral and bilateral subthalamic nucleus deep brain stimulation. Neurology 53, 561–566. 10.1212/WNL.53.3.561 10449121

[B100] Levkovitz Y. Isserles M. Padberg F. Lisanby S. H. Bystritsky A. Xia G.. (2015). Efficacy and safety of deep transcranial magnetic stimulation for major depression: a prospective multicenter randomized controlled trial. World Psychiatry 14, 64–73. 10.1002/wps.20199 25655160 PMC4329899

[B101] Lewis C. P. Camsari D. D. Sonmez A. I. Nandakumar A. L. Gresbrink M. A. Daskalakis Z. J.. (2019). Preliminary evidence of an association between increased cortical inhibition and reduced suicidal ideation in adolescents treated for major depression. J. Affect. Disord. 244, 21–24. 10.1016/j.jad.2018.09.079 30292987 PMC6231405

[B102] Lhommee E. Klinger H. Thobois S. Schmitt E. Ardouin C. Bichon A.. (2012). Subthalamic stimulation in Parkinson's disease: restoring the balance of motivated behaviours. Brain 135(Pt. 5), 1463–1477. 10.1093/brain/aws078 22508959

[B103] Liang C. S. Chung C. H. Ho P. S. Tsai C. K. Chien W. C. (2018). Superior anti-suicidal effects of electroconvulsive therapy in unipolar disorder and bipolar depression. Bipolar. Disord. 20, 539–546. 10.1111/bdi.12589 29227012

[B104] Lilleeng B. Dietrichs E. (2008). Unmasking psychiatric symptoms after STN deep brain stimulation in Parkinson's disease. Acta Neurol. Scand. Suppl. 188, 41–45. 10.1111/j.1600-0404.2008.01030.x 18439220

[B105] Lillethorup T. P. Iversen P. Fontain J. Wegener G. Doudet D. J. Landau A. M. (2015). Electroconvulsive shocks decrease alpha2-adrenoceptor binding in the Flinders rat model of depression. Eur. Neuropsychopharmacol. 25, 404–412. 10.1016/j.euroneuro.2014.12.003 25604421

[B106] Lozano A. M. Giacobbe P. Hamani C. Rizvi S. J. Kennedy S. H. Kolivakis T. T.. (2012). A multicenter pilot study of subcallosal cingulate area deep brain stimulation for treatment-resistant depression. J. Neurosurg. 116, 315–322. 10.3171/2011.10.JNS102122 22098195

[B107] Michael N. Erfurth A. Ohrmann P. Arolt V. Heindel W. Pfleiderer B. (2003). Metabolic changes within the left dorsolateral prefrontal cortex occurring with electroconvulsive therapy in patients with treatment resistant unipolar depression. Psychol. Med. 33, 1277–1284. 10.1017/S0033291703007931 14580081

[B108] Middleton F. A. Strick P. L. (1994). Anatomical evidence for cerebellar and basal ganglia involvement in higher cognitive function. Science 266, 458–461. 10.1126/science.7939688 7939688

[B109] Milstein V. Small J. G. Small I. F. Green G. E. (1986). Does electroconvulsive therapy prevent suicide? Convuls. Ther. 2, 3–6. 11940839

[B110] Mitchell S. Hassan E. Ghaziuddin N. (2018). A follow-up study of electroconvulsive therapy in children and adolescents. J. ECT 34, 40–44. 10.1097/YCT.0000000000000452 28937548

[B111] Montgomery S. A. Asberg M. (1979). A new depression scale designed to be sensitive to change. Br. J. Psychiatry 134, 382–389. 10.1192/bjp.134.4.382 444788

[B112] Moro E. Scerrati M. Romito L. M. Roselli R. Tonali P. Albanese A. (1999). Chronic subthalamic nucleus stimulation reduces medication requirements in Parkinson's disease. Neurology 53, 85–90. 10.1212/WNL.53.1.85 10408541

[B113] Munk-Olsen T. Munk Laursen T. Videbech P. Mortensen P. B. Rosenberg R. (2007). All-cause mortality among recipients of electroconvulsive therapy: register-based cohort study. Br. J. Psychiatry 190, 435–439. 10.1192/bjp.bp.106.026740 17470959

[B114] Myslobodsky M. Lalonde F. M. Hicks L. (2001). Are patients with Parkinson's disease suicidal? J. Geriatr. Psychiatry Neurol. 14, 120–124. 10.1177/089198870101400304 11563434

[B115] Nahas Z. Marangell L. B. Husain M. M. Rush A. J. Sackeim H. A. Lisanby S. H.. (2005). Two-year outcome of vagus nerve stimulation (VNS) for treatment of major depressive episodes. J. Clin. Psychiatry 66, 1097–1104. 10.4088/JCP.v66n0902 16187765

[B116] Nordenskjold A. von Knorring L. Ljung T. Carlborg A. Brus O. Engstrom I. (2013). Continuation electroconvulsive therapy with pharmacotherapy versus pharmacotherapy alone for prevention of relapse of depression: a randomized controlled trial. J. ECT 29, 86–92. 10.1097/YCT.0b013e318276591f 23303421

[B117] Ogbonnaya S. Kaliaperumal C. (2013). Vagal nerve stimulator: evolving trends. J. Nat. Sci. Biol. Med. 4, 8–13. 10.4103/0976-9668.107254 23633829 PMC3633308

[B118] Olin B. Jayewardene A. K. Bunker M. Moreno F. (2012). Mortality and suicide risk in treatment-resistant depression: an observational study of the long-term impact of intervention. PLoS. ONE 7:e48002. 10.1371/journal.pone.0048002 23133537 PMC3485051

[B119] O'Reardon J. P. Solvason H. B. Janicak P. G. Sampson S. Isenberg K. E. Nahas Z.. (2007). Efficacy and safety of transcranial magnetic stimulation in the acute treatment of major depression: a multisite randomized controlled trial. Biol. Psychiatry 62, 1208–1216. 10.1016/j.biopsych.2007.01.018 17573044

[B120] Ozsoy S. Olguner Eker O. Abdulrezzak U. (2016). The effects of antidepressants on neuropeptide y in patients with depression and anxiety. Pharmacopsychiatry 49, 26–31. 10.1055/s-0035-1565241 26789271

[B121] Page M. J. McKenzie J. E. Bossuyt P. M. Boutron I. Hoffmann T. C. Mulrow C. D. The PRISMA 2020 statement: an updated guideline for reporting systematic reviews. BMJ. (2021). 372:n71. 10.1136/bmj.n71 33782057 PMC8005924

[B122] Patel M. Patel S. Hardy D. W. Benzies B. J. Tare V. (2006). Should electroconvulsive therapy be an early consideration for suicidal patients? J. ECT 22, 113–115. 10.1097/00124509-200606000-00007 16801826

[B123] Petrosino N. J. Wout-Frank M. V. Aiken E. Swearingen H. R. Barredo J. Zandvakili A.. (2020). One-year clinical outcomes following theta burst stimulation for post-traumatic stress disorder. Neuropsychopharmacology 45, 940–946. 10.1038/s41386-019-0584-4 31794974 PMC7162862

[B124] Pinsker M. Amtage F. Berger M. Nikkhah G. van Elst L. T. (2013). Psychiatric side-effects of bilateral deep brain stimulation for movement disorders. Acta. Neurochir. Suppl. 117, 47–51. 10.1007/978-3-7091-1482-7_8 23652656

[B125] Porat O. Cohen O. S. Schwartz R. Hassin-Baer S. (2009). Association of preoperative symptom profile with psychiatric symptoms following subthalamic nucleus stimulation in patients with Parkinson's disease. J. Neuropsychiatry Clin. Neurosci. 21, 398–405. 10.1176/jnp.2009.21.4.398 19996248

[B126] Posner K. Brown G. K. Stanley B. Brent D. A. Yershova K. V. Oquendo M. A.. (2011). The Columbia-suicide severity rating scale: initial validity and internal consistency findings from three multisite studies with adolescents and adults. Am. J. Psychiatry 168, 1266–1277. 10.1176/appi.ajp.2011.10111704 22193671 PMC3893686

[B127] Poznanski E. O. Grossman J. A. Buchsbaum Y. Banegas M. Freeman L. Gibbons R. (1984). Preliminary studies of the reliability and validity of the children's depression rating scale. J. Am. Acad. Child Psychiatry 23, 191–197. 10.1097/00004583-198403000-00011 6715741

[B128] Rao V. Bechtold K. McCann U. Roy D. Peters M. Vaishnavi S.. (2019). Low-frequency right repetitive transcranial magnetic stimulation for the treatment of depression after traumatic brain injury: a randomized sham-controlled pilot study. J. Neuropsychiatry Clin. Neurosci. 31, 306–318. 10.1176/appi.neuropsych.17110338 31018810

[B129] Rapinesi C. Kotzalidis G. D. Curto M. Serata D. Ferri V. R. Scatena P.. (2015). Electroconvulsive therapy improves clinical manifestations of treatment-resistant depression without changing serum BDNF levels. Psychiatry Res. 227, 171–178. 10.1016/j.psychres.2015.04.009 25910420

[B130] Ray-Griffith S. L. Coker J. L. Rabie N. Eads L. A. Golden K. J. Stowe Z. N. (2016). Pregnancy and electroconvulsive therapy: a multidisciplinary approach. J. ECT 32, 104–112. 10.1097/YCT.0000000000000297 26796501 PMC4877273

[B131] Raymaekers S. Luyten L. Bervoets C. Gabriels L. Nuttin B. (2017). Deep brain stimulation for treatment-resistant major depressive disorder: a comparison of two targets and long-term follow-up. Transl. Psychiatry 7:e1251. 10.1038/tp.2017.66 29087373 PMC5682606

[B132] Rektorova I. (2019). Current treatment of behavioral and cognitive symptoms of Parkinson's disease. Parkinsonism Relat. Disord. 59, 65–73. 10.1016/j.parkreldis.2019.02.042 30852149

[B133] Revesz D. Tjernstrom M. Ben-Menachem E. Thorlin T. (2008). Effects of vagus nerve stimulation on rat hippocampal progenitor proliferation. Exp. Neurol. 214, 259–265. 10.1016/j.expneurol.2008.08.012 18804463

[B134] Review Manager (RevMan) (2014). RevMan 5.3 User Guide [Computer Program]. Version 5.3. Copenhagen: The Nordic Cochrane Centre, The Cochrane Collaboration. Available online at: https://training.cochrane.org/sites/training.cochrane.org/files/public/uploads/resources/downloadable_resources/English/RevMan_5.3_User_Guide.pdf

[B135] Rich C. L. Spiker D. G. Jewell S. W. Neil J. F. (1986). Response of energy and suicidal ideation to ECT. J. Clin. Psychiatry 47, 31–32. 3941054

[B136] Richard-Devantoy S. Berlim M. T. Jollant F. (2015). Suicidal behaviour and memory: a systematic review and meta-analysis. World J. Biol. Psychiatry 16, 544–566. 10.3109/15622975.2014.925584 25112792

[B137] Rihmer Z. (2007). Suicide risk in mood disorders. Curr. Opin. Psychiatry 20, 17–22. 10.1097/YCO.0b013e3280106868 17143077

[B138] Rodriguez M. C. Guridi O. J. Alvarez L. Mewes K. Macias R. Vitek J.. (1998). The subthalamic nucleus and tremor in Parkinson's disease. Mov. Disord. 13(Suppl. 3), 111–118. 10.1002/mds.870131320 9827606

[B139] Ronnqvist I. Brus O. Hammar A. Landen M. Lundberg J. Nordanskog P.. (2019). Rehospitalization of postpartum depression and psychosis after electroconvulsive therapy: a population-based study with a matched control group. J. ECT 35, 264–271. 10.1097/YCT.0000000000000578 31764450 PMC6903363

[B140] Roosevelt R. W. Smith D. C. Clough R. W. Jensen R. A. Browning R. A. (2006). Increased extracellular concentrations of norepinephrine in cortex and hippocampus following vagus nerve stimulation in the rat. Brain Res. 1119, 124–132. 10.1016/j.brainres.2006.08.048 16962076 PMC1751174

[B141] Rush A. J. Marangell L. B. Sackeim H. A. George M. S. Brannan S. K. Davis S. M.. (2005a). Vagus nerve stimulation for treatment-resistant depression: a randomized, controlled acute phase trial. Biol. Psychiatry 58, 347–354. 10.1016/j.biopsych.2005.05.025 16139580

[B142] Rush A. J. Sackeim H. A. Marangell L. B. George M. S. Brannan S. K. Davis S. M.. (2005b). Effects of 12 months of vagus nerve stimulation in treatment-resistant depression: a naturalistic study. Biol. Psychiatry 58, 355–363. 10.1016/j.biopsych.2005.05.024 16139581

[B143] Rush A. J. Trivedi M. H. Ibrahim H. M. Carmody T. J. Arnow B. Klein D. N.. (2003). The 16-Item Quick Inventory of Depressive Symptomatology (QIDS), clinician rating (QIDS-C), and self-report (QIDS-SR): a psychometric evaluation in patients with chronic major depression. Biol. Psychiatry 54, 573–583. 10.1016/S0006-3223(02)01866-8 12946886

[B144] Sartorius A. Mahlstedt M. M. Vollmayr B. Henn F. A. Ende G. (2007). Elevated spectroscopic glutamate/gamma-amino butyric acid in rats bred for learned helplessness. Neuroreport 18, 1469–1473. 10.1097/WNR.0b013e3282742153 17712276

[B145] Schlaepfer T. E. Frick C. Zobel A. Maier W. Heuser I. Bajbouj M.. (2008). Vagus nerve stimulation for depression: efficacy and safety in a European study. Psychol. Med. 38, 651–661. 10.1017/S0033291707001924 18177525

[B146] Schmaal L. van Harmelen A. L. Chatzi V. Lippard E. T. C. Toenders Y. J. Averill L. A.. (2020). Imaging suicidal thoughts and behaviors: a comprehensive review of 2 decades of neuroimaging studies. Mol. Psychiatry 25, 408–427. 10.1038/s41380-019-0587-x 31787757 PMC6974434

[B147] Sharma V. (1999). Retrospective controlled study of inpatient ECT: does it prevent suicide? J. Affect. Disord. 56, 183–187. 10.1016/S0165-0327(98)00207-9 10701475

[B148] Sheehan D. V. Lecrubier Y. Sheehan K. H. Amorim P. Janavs J. Weiller E.. (1998). The Mini-International Neuropsychiatric Interview (MINI): the development and validation of a structured diagnostic psychiatric interview for DSM-IV and ICD-10. J. Clin. Psychiatry 59(Suppl. 20), 22–33. 9881538

[B149] Shepard M. D. Perepezko K. Broen M. P. G. Hinkle J. T. Butala A. Mills K. A.. (2019). Suicide in Parkinson's disease. J. Neurol. Neurosurg. Psychiatry 90, 822–829. 10.1136/jnnp-2018-319815 30661029 PMC7187903

[B150] Shilton T. Enoch-Levy A. Giron Y. Yaroslavsky A. Amiaz R. Gothelf D.. (2020). A retrospective case series of electroconvulsive therapy in the management of comorbid depression and anorexia nervosa. Int. J. Eat. Disord. 53, 210–218. 10.1002/eat.23181 31639233

[B151] Singh A. Kar S. K. (2017). How electroconvulsive therapy works? Understanding the neurobiological mechanisms. Clin. Psychopharmacol. Neurosci. 15, 210–221. 10.9758/cpn.2017.15.3.210 28783929 PMC5565084

[B152] Smeding H. M. Speelman J. D. Koning-Haanstra M. Schuurman P. R. Nijssen P. van Laar T.. (2006). Neuropsychological effects of bilateral STN stimulation in Parkinson disease: a controlled study. Neurology 66, 1830–1836. 10.1212/01.wnl.0000234881.77830.66 16801645

[B153] Smith K. A. Fairburn C. G. Cowen P. J. (1997). Relapse of depression after rapid depletion of tryptophan. Lancet 349, 915–919. 10.1016/S0140-6736(96)07044-4 9093253

[B154] Soulas T. Gurruchaga J. M. Palfi S. Cesaro P. Nguyen J. P. Fenelon G. (2008). Attempted and completed suicides after subthalamic nucleus stimulation for Parkinson's disease. J. Neurol. Neurosurg. Psychiatry 79, 952–954. 10.1136/jnnp.2007.130583 18403439

[B155] Staudt M. D. Herring E. Z. Gao K. Miller J. P. Sweet J. A. (2019). Evolution in the treatment of psychiatric disorders: from psychosurgery to psychopharmacology to neuromodulation. Front. Neurosci. 13:108. 10.3389/fnins.2019.00108 30828289 PMC6384258

[B156] Stefansson J. Nordstrom P. Jokinen J. (2012). Suicide intent scale in the prediction of suicide. J. Affect. Disord. 136, 167–171. 10.1016/j.jad.2010.11.016 21144592

[B157] Strutt A. M. Simpson R. Jankovic J. York M. K. (2012). Changes in cognitive-emotional and physiological symptoms of depression following STN-DBS for the treatment of Parkinson's disease. Eur. J. Neurol. 19, 121–127. 10.1111/j.1468-1331.2011.03447.x 21668586

[B158] Sun Y. Blumberger D. M. Mulsant B. H. Rajji T. K. Fitzgerald P. B. Barr M. S.. (2018). Magnetic seizure therapy reduces suicidal ideation and produces neuroplasticity in treatment-resistant depression. Transl. Psychiatry 8:253. 10.1038/s41398-018-0302-8 30470735 PMC6251931

[B159] Sun Y. Farzan F. Mulsant B. H. Rajji T. K. Fitzgerald P. B. Barr M. S.. (2016). Indicators for remission of suicidal ideation following magnetic seizure therapy in patients with treatment-resistant depression. JAMA Psychiatry 73, 337–345. 10.1001/jamapsychiatry.2015.3097 26981889

[B160] Temel Y. Boothman L. J. Blokland A. Magill P. J. Steinbusch H. W. Visser-Vandewalle V.. (2007). Inhibition of 5-HT neuron activity and induction of depressive-like behavior by high-frequency stimulation of the subthalamic nucleus. Proc. Natl. Acad. Sci. U.S.A. 104, 17087–17092. 10.1073/pnas.0704144104 17942692 PMC2040465

[B161] Temel Y. Tan S. Visser-Vandewalle V. Sharp T. (2009). Parkinson's disease, DBS and suicide: a role for serotonin? Brain 132(Pt. 10):e126. 10.1093/brain/awp150 19553275

[B162] Thobois S. Ardouin C. Lhommee E. Klinger H. Lagrange C. Xie J.. (2010). Non-motor dopamine withdrawal syndrome after surgery for Parkinson's disease: predictors and underlying mesolimbic denervation. Brain 133(Pt. 4), 1111–1127. 10.1093/brain/awq032 20237128

[B163] Tirmizi O. Raza A. Trevino K. Husain M. M. (2012). Electroconvulsive therapy. How modern techniques improve patient outcomes: refinements have decreased memory loss, other adverse effects while retaining efficacy. Refinements have decreased memory loss, other adverse effects while retaining efficacy. Curr. Psychiatr. 11, 24–46. 25311628 PMC4193538

[B164] Tsuang M. T. Dempsey G. M. Fleming J. A. (1979). Can ECT prevent premature death and suicide in 'schizoaffective' patients? J. Affect. Disord. 1, 167–171. 10.1016/0165-0327(79)90001-6 162499

[B165] Umemura A. Oka Y. Yamamoto K. Okita K. Matsukawa N. Yamada K. (2011). Complications of subthalamic nucleus stimulation in Parkinson's disease. Neurol. Med. Chir. 51, 749–755. 10.2176/nmc.51.749 22123476

[B166] Valiengo L. Bensenor I. M. Goulart A. C. de Oliveira J. F. Zanao T. A. Boggio P. S.. (2013). The sertraline versus electrical current therapy for treating depression clinical study (select-TDCS): results of the crossover and follow-up phases. Depress. Anxiety 30, 646–653. 10.1002/da.22079 23625554

[B167] van der Wal J. M. Bergfeld I. O. Lok A. Mantione M. Figee M. Notten P.. (2020). Long-term deep brain stimulation of the ventral anterior limb of the internal capsule for treatment-resistant depression. J. Neurol. Neurosurg. Psychiatry 91, 189–195. 10.1136/jnnp-2019-321758 31801845 PMC6996094

[B168] Velasco F. Velasco M. Jiménez F. Velasco A. L. Salin-Pascual R. (2005). Neurobiological background for performing surgical intervention in the inferior thalamic peduncle for treatment of major depression disorders. Neurosurgery 57, 439–448; discussion 439–448. 10.1227/01.NEU.0000172172.51818.51 16145522

[B169] Volkmann J. Wolters A. Kupsch A. Muller J. Kuhn A. A. Schneider G. H.. (2012). Pallidal deep brain stimulation in patients with primary generalised or segmental dystonia: 5-year follow-up of a randomised trial. Lancet Neurol. 11, 1029–1038. 10.1016/S1474-4422(12)70257-0 23123071

[B170] Voon V. Krack P. Lang A. E. Lozano A. M. Dujardin K. Schupbach M.. (2008). A multicentre study on suicide outcomes following subthalamic stimulation for Parkinson's disease. Brain 131(Pt. 10), 2720–2728. 10.1093/brain/awn214 18941146 PMC2724899

[B171] Wall C. A. Croarkin P. E. Maroney-Smith M. J. Haugen L. M. Baruth J. M. Frye M. A.. (2016). Magnetic resonance imaging-guided, open-label, high-frequency repetitive transcranial magnetic stimulation for adolescents with major depressive disorder. J. Child. Adolesc. Psychopharmacol. 26, 582–589. 10.1089/cap.2015.0217 26849202 PMC5035831

[B172] Wall C. A. Croarkin P. E. Sim L. A. Husain M. M. Janicak P. G. Kozel F. A.. (2011). Adjunctive use of repetitive transcranial magnetic stimulation in depressed adolescents: a prospective, open pilot study. J. Clin. Psychiatry 72, 1263–1269. 10.4088/JCP.11m07003 21951987

[B173] Weintraub D. Duda J. E. Carlson K. Luo P. Sagher O. Stern M.. (2013). Suicide ideation and behaviours after STN and GPi DBS surgery for Parkinson's disease: results from a randomised, controlled trial. J. Neurol. Neurosurg. Psychiatry 84, 1113–1118. 10.1136/jnnp-2012-304396 23667214 PMC4594869

[B174] Weissman C. R. Blumberger D. M. Brown P. E. Isserles M. Rajji T. K. Downar J.. (2018). Bilateral repetitive transcranial magnetic stimulation decreases suicidal ideation in depression. J. Clin. Psychiatry 79:17m11692. 10.4088/JCP.17m11692 29701939

[B175] Williams A. Gill S. Varma T. Jenkinson C. Quinn N. Mitchell R.. (2010). Deep brain stimulation plus best medical therapy versus best medical therapy alone for advanced Parkinson's disease (PD SURG trial): a randomised, open-label trial. Lancet Neurol. 9, 581–591. 10.1016/S1474-4422(10)70093-4 20434403 PMC2874872

[B176] Yesavage J. A. Fairchild J. K. Mi Z. Biswas K. Davis-Karim A. Phibbs C. S.. (2018). Effect of repetitive transcranial magnetic stimulation on treatment-resistant major depression in US veterans: a randomized clinical trial. JAMA Psychiatry 75, 884–893. 10.1001/jamapsychiatry.2018.1483 29955803 PMC6142912

[B177] Yin H. Pantazatos S. P. Galfalvy H. Huang Y. Y. Rosoklija G. B. Dwork A. J.. (2016). A pilot integrative genomics study of GABA and glutamate neurotransmitter systems in suicide, suicidal behavior, and major depressive disorder. Am. J. Med. Genet. B. europsychiatr. Genet. 171b, 414–426. 10.1002/ajmg.b.32423 26892569 PMC4851346

[B178] Youseff H. A. (1990). Electroconvulsive therapy and benzodiazepine use in patients who committed suicide. Adv. Ther. 7, 153–158. 10149183

[B179] Yu X. X. Fernandez H. H. (2017). Dopamine agonist withdrawal syndrome: a comprehensive review. J. Neurol. Sci. 374, 53–55. 10.1016/j.jns.2016.12.070 28104232

[B180] Zhao J. Verwer R. W. H. Gao S. F. Qi X. R. Lucassen P. J. Kessels H. W.. (2018). Prefrontal alterations in GABAergic and glutamatergic gene expression in relation to depression and suicide. J. Psychiatr. Res. 102, 261–274. 10.1016/j.jpsychires.2018.04.020 29753198

