# Supplementary material for: A Systematic Review of Neuromodulation Treatment Effects on Suicidality
Source: Front Hum Neurosci. 2021 Jun 25;15:660926. doi: 10.3389/fnhum.2021.660926 (PMC8267816; doi:10.3389/fnhum.2021.660926)
Supplement: Supplementary file 1 [file Table_1.docx]

**Supplementary Table 1.** **Noninvasive Brain Stimulation Studies**

| **Authors** | **Design** | **Population** | **No. patients** | **No. controls** | **NIBS type** | **Treatment parameters** | **Treatment duration** | **Suicidality outcome/measure** | | **Results** |  |
| --- | --- | --- | --- | --- | --- | --- | --- | --- | --- | --- | --- |
| Abdelnaim et al, 2019 | Retrospective cohort study | 332 inpatients/ outpatients with depressive affective disorder | 332 | None | rTMS | 78.6% of patients: 20 Hz, left prefrontal, 2,000 pulses;  5.7% of patients: iTBS, left prefrontal followed by cTBS, right prefrontal, 2,400 pulses;  4.5% of patients: 10 Hz, left prefrontal, 2,000 pulses;  3.0% of patients: 10 Hz, left prefrontal, 1,000 pulses | 17.0±6.5 sessions | Suicidal ideation | | Of patients, 47% had improved suicidal ideation, as measured by item 3 of HDRS; 41.3% of patients did not improve in suicidal ideation.  There was a significant positive correlation between improvement in suicidal ideation and increase in drive, as measured by item 7 of HDRS (*P<*.001). |  |
| Baeken et al, 2017 | Randomized, double-blind, sham-controlled | 50 patients with TRD^a^ | 50 | None | aiTBS | 20 sessions:  Left DLPFC: 1,620 pulses per session in 54 triplet bursts with a train duration of 2 s and an intertrain interval of 8 s  Stimulation intensity was 110% of the patient’s resting MT | 4 d, 5 sessions/d | Suicidal ideation | | Clinical responders had significantly lower suicidal ideation than nonresponders (2.67±6.28 vs 8.38±10.98, *P<*.05, as measured by SSI).  Clinical responders had significantly lower levels of hopelessness than nonresponders.  Correlation analysis showed a significant correlation between depressive symptom improvement and improvement in suicidal ideation. |  |
| Baeken et al, 2019 | Randomized, double-blind, sham-controlled, crossover trial | 45 patients with TRD^a^ | Active iTBS: 21 patients had active treatment in the first 2 wk and sham stimulation in the second 2-wk period | Sham iTBS: 24 patients had sham treatment in the first 2 wk and active stimulation in the second 2-wk period | iTBS | Left DLPFC: 20 iTBS sessions over 4 d, 5 sessions/d; total, 32,400 sessions; 1,620 pulses per session in 54 triplet bursts with a duration of 2 s and 8 s intertrain interval | 4 d, 5 sessions/d | Suicidal ideation | | There were no significant differences between groups in baseline BSI scores.  There was a significant decrease in BSI scores in the sham group compared with baseline at the end of the first treatment wk (*P=.*03), and this difference did not exist in the active-treatment group (*P*=.06).  There was no significant difference between active and sham groups in the first or second wk of treatment (*P*=.53 and *P*=.17, respectively). |  |
| Berlim et al, 2014 | Prospective, open-label, nonrandomized observational study | 17 outpatients with TRD^b^ | 17 | None | dTMS | Left DLPFC: 20 Hz in 2 s trains with 75 trains/session, totaling 3,000 pulses/session, 120% of RMT total, 60,000 pulses | 4 wk of daily dTMS totaling 20 sessions | Suicidal ideation | | Suicidality ratings, as measured by SSI, were significantly improved by wk 5 (before: 10.88±9.25; after: 8.12±9.41; *P*=.02, as measured by SSI).  Significant improvement in depression scores, as measured by HDRS-21 *(P<*.001). |  |
| Bloch et al, 2008 | Prospective, open-label pilot study | 9 adolescents with TRD^c^ | 9 | None | rTMS | Left DLPFC: stimulus intensity, 80% of the patient’s MT intensity  Treatments at 10 Hz for 2 s/train, with an intertrain interval of 58 s | 14 sessions over 14 working d | Suicide attempt and suicidal ideation | | One patient had a suicide attempt 3 wk after surgery.  Suicidal ideation level was not significantly affected by rMTS therapy, as measured by the Suicidal Ideation Questionnaire (before treatment: 100±46.9 vs after: 88±54.6).  Patients had significantly lower levels of depression and anxiety and showed significant clinical improvement at the end of the study and follow-up. |  |
| Blumberger et al, 2016 | Randomized controlled trial | Patients with TRD^a^ | 121 patients with TRD | Bilateral TMS, 40 patients | TMS | Unilateral, left DLPFC: 10 Hz, 120% adjusted RMT; 30 pulses/train, 70 trains; intertrain interval: 30 s; total pulses, 2,100  Bilateral, first 600 pulses right DLPFC: 1 Hz, 120% adjusted RMT, 100 pulses per train, 6 trains; intertrain interval, 30 s; later, 1,500 pulses; left DLPFC: 10 Hz, 120 adjusted RMT; 20 pulses/train, 70 trains; intertrain interval, 30 s | First phase: 5 d/wk over 3 wk;  Second phase: Nonremitters continued treatment 5 d/wk over 3 wk | Suicidal ideation | | No patients had suicidal thoughts in both unilateral and bilateral groups; 2.4% in the sham-controlled group.  Based on HDRS-17, the remission rate was significantly higher for bilateral stimulation than sham stimulation (*P*=.01). The remission rate did not differ significantly between the unilateral and sham-stimulation groups (*P*=.35).  Based on BDI-II, remission results were similar. Remission was significantly higher in the bilateral stimulation group than the sham-stimulation group (*P*=.02), and no significant difference was shown between the unilateral and sham groups (*P*=.99). |  |
| Brunoni et al, 2014 | Double-blind placebo-controlled trial | 120 outpatients with an acute depressive episode | 30 patients receiving tDCS alone; 30 patients, combined tDCS plus sertraline | 30 patients receiving sham tDCS plus placebo sertraline; 30 patients, sertraline only | tDCS | Current density of 0.8 A/m^2^ (2 mA/25 cm^2^) per 30 min/d | 10 daily sessions plus 2 additional fortnight sessions | Suicidal ideation | Comparison of tDCS-only vs placebo showed that tDCS was significantly more effective in decreasing suicidal thoughts (*P<.*01).  Combined treatment vs placebo reached the same significance in improvement of suicidal thoughts (*P<.*01).  When the sertraline-only group was compared with the placebo group, suicidal thoughts tended to improve, but the difference was not significant.  There were 2 cases of suicidal ideation: 1 in the placebo group and the other in the tDCS-only group. | |  |
| Carpenter et al, 2017 | Randomized, double-blind, sham-controlled study | 92 patients with TRD or treatment intolerant depression | 47 patients treated with active rTMS | 45 patients treated with sham treatment | TMS | 2-coil rTMS device; left DLPFC: 10 Hz in trains of 4-s stimulation followed by 26-s rest. A total of 3,000 pulses/session delivered. The intensity was ≤120% of the participant’s left-sided resting MT. | 20 sessions over 4-6 wk | Suicide attempt | | One patient in the sham group attempted suicide. |  |
| Croarkin et al, 2018 | Prospective open-label trial | 19 TRD adolescents^d,e^ | 19 | None | High-frequency rTMS | 10 Hz, 120% MT treatment delivered to the left dorsolateral prefrontal cortex in 4-s stimulus trains separated by 26-s intertrain intervals, with 3,000 magnetic pulses/session | 30 sessions over 6-8 wk | Suicidal ideation | | Risk of suicidal ideation decreased after 6 wk of treatment (OR=0.407 [95% CI, 0.265-0.625]; *P<*.001), as measured by C-SSRS, Intensity of Ideation Subscale, and item 13 (suicidality) on CDRS-R.  When adjusted for depression severity, decreased risk of suicidal ideation was no longer significant. |  |
| Desmyter et al, 2014 | Randomized, sham-controlled crossover study | 12 treatment resistant,^a^ suicidal depressed patients | 12 | None | Accelerated rTMS | Left DLPFC: patients received 1,620 pulses per session in 54 bursts of 3 with a train duration of 2 s and an intertrain interval of 8 s;  Stimulation intensity was 100% of a patient’s MT | 20 sessions over 4 d | Suicidal ideation | | There was a significant decrease in suicidal ideation, as measured by SSI scores compared with baseline (*P*=.01). This decrease was independent of the order of treatment (*P*=.24).  The decrease in suicidal ideation was independent of the change in depression severity, as measured by HDRS (*P*=.93). |  |
| Desmyter et al, 2016 | Randomized, sham-controlled crossover study | 50 TRD patients^a^ | 50 | None | aiTBS | Left DLPFC: 54 trains of 10 burst theta stimuli;  50 Hz stimuli delivered in 2 s with interstimulus interval of 8 s;  1,620 stimuli/session | 20 sessions over 4 d | Suicidal ideation | | There was a significant decrease in suicidal ideation, as measured by BSI score, starting from wk 1 and lasting up to 1 mo after baseline.  This decrease was independent of the order of treatment and change of depression severity.  No worsening in suicidal ideation was observed.  None of the participants had committed suicide at 6-mo follow-up. |  |
| Duprat et al, 2016 | Randomized, double-blind, sham-controlled, crossover trial | Patients with TRD^a^ | 47 patients with TRD | Patients are own controls | iTBS | 54 triple bursts delivered in 2-s trains with an intertrain interval of 8 s; total of 1,620 pulses/session;  Unilateral: left DLPFC, stimulation intensity 110% of MT | 20 iTBS sessions over 4 d, 5 sessions/d; total, 32,400 stimuli | Suicide attempt | | One female patient in the sham group had a severe suicide attempt (medication overdose) after 1 wk of sham stimulation. |  |
| Fitzgerald et al, 2018 | 2-group, single-blind, randomized controlled trial | 119 patients with TRD^a^ | 60 patients in the accelerated treatment group | 60 patients in the standard treatment group | Accelerated rTMS | Accelerated treatment:  3 treatment sessions/d, each with 83, 83, and 84 trains. Trains consisted of 10 Hz rTMS to the left DLPFC, with 4.2-s trains applied at 120% of the resting MT with a 15-s intertrain interval (10,500 pulses/d across 3 sessions, 63,000 pulses total)  Standard treatment:  75 trains of 10 Hz rTMS to the DLPFC, with 4.2-s long trains applied at 120% of RMT with a 15-s intertrain interval (10,500 pulses/d across the 3 sessions, 63,000 pulses in total) | Accelerated treatment:  wk 1, 3 treatments/d over 3 days;  wk 2, 3 treatments/d over 2 days;  wk 3, 3 treatments in 1 d  Standard treatment:  Total of 20 treatments, 5 d/wk over 4 wk | Suicidal ideation | | At any time point, no significant difference was shown in improvement of suicidal ideation, as measured by SSI, between groups (*P*=.68). Both groups had significant improvements in SSI scores over time (*P*=.02).  At any time point, improvement in depression, as measured by HDRS, was similar to improvement in suicidal ideation. Both groups had significant improvements compared with baseline, but there were no significant differences between the groups regarding improvement, response, and remission (*P*=.12, *P*=.24, and *P*=.39, respectively).  At any time point, there was no significant difference between the groups regarding response and remission rates of depression as measured by MADRS (*P*=.25 and *P*=.64 for the 4-wk assessment and *P*=.60 and *P*=.93 for the 8-wk assessment). |  |
| Fitzgerald et al, 2020 | 2-group, single-blind, randomized controlled trial^d^ | 74 outpatients with TRD | 36 patients had aiTBS | 38 patients had standard rTMS treatment | Accelerated TBS and rTMS treatment | Accelerated iTBS, left DLPFC: triple TMS pulses at 50 Hz, delivered in 2-s trains and repeated every 10 s for a total of 190 s, totaling 600 pulses/session and 12,600 pulses overall. Stimulation intensity was 120% of RMT.  Standard rTMS, left DLPFC: 10 Hz stimulation delivered in 4.2-s trains with 25-s intertrain intervals. Total of 3,150 rTMS pulses/session, which totals 63,000 pulses over 20 treatment days.  Stimulation intensity was 120% of RMT | Accelerated iTBS:  wk 1: 3 sessions/d for 3 d  wk 2: 3 sessions/d for 2 d  wk 3: 3 sessions/d for 1 d  wk 4: 3 sessions/d for 1 d  Standard rTMS: 4 sessions/wk over 5 wk, total of 20 sessions | Suicidal ideation | | There was no significant difference between groups in terms of suicidal ideation intensity as measured by C-SSRS scores (*P*=.99) and also by MADRS suicidal ideation score. However, both groups had significant decreases in suicidal ideation intensity over time (*P<*.001).  There was no significant difference between groups regarding response and remission rate at 4 or 8 wk, as measured by MADRS.  No significant difference was shown between groups in any cognitive domain assessed, including information processing and complex attention and executive function. |  |
| George et al, 2010 | Randomized sham-controlled trial | Patients with MDD | 92 patients treated with left DLPFC stimulation daily | 88 patients treated with sham stimulation | rTMS | Left PFC; stimulation intensity: 120% of MT; stimulation frequency: 10 Hz, 4-s train duration and 26-s intertrain interval; treatment duration: 37.5 min; total pulses/session, 3,000 | TMS treatment was applied over 3 wk | Suicide completion | | There were no cases of completed suicide.  The treatment group had significantly higher remission and response rates (*P*=.009 for ITT and *P*=.02 for analysis of completers). |  |
| George, et al, 2014 | Randomized sham-controlled trial | 42 patients with acute suicidal ideation | 20 patients treated with active stimulation | 21 patients treated with sham stimulation | rTMS | Left prefrontal cortex, figure-8 solid core coil at 120% MT, 10 Hz, 5-s train duration, 10-s intertrain interval for 30 min (6,000 pulses, 54,000 total stimuli) | 3 times daily for 3 d  (total 9 sessions) | Suicidal ideation | | In completer’s analysis, the TMS group had a significantly higher decrease in suicidal ideation, as measured by SSI, than the sham group (*P*=.03).  The ITT analysis was not significant (*P*=.05).  During 6-mo follow-up, 4 patients in the sham group and 7 in the TMS group were hospitalized, mainly due to suicidal ideation and attempt.  No significant difference was found (*P*=.34). |  |
| Hadley, et al, 2011 | Prospective observational study | 19 patients with current MDE (unipolar or bipolar depression) | 19 | None | rTMS | Left prefrontal: 120% of resting MT, 10 Hz, 5 s on and 10 s off for a mean of 6,800 stimuli/session | 5 sessions/wk, duration of treatment depended on clinical improvement | Suicidal ideation | | Suicidal ideation, as measured by SSI scores, decreased significantly per TMS session within the first month (*P=*.03), within 2 mo (*P*=.004), and throughout the study (*P*=.001).  Suicidal ideation diminished in 67% of patients after 1 wk. |  |
| Holtzheimer et al, 2010 | Prospective, open-label single-group study | 14 patients with current MDE resistant to at least 1 medication(1 patient had bipolar disorder) | 14 | None | Accelerated rTMS | Left DLPFC: 10 Hz rTMS in 5-s trains with a 25-s intertrain interval at 100% MT intensity. Total of 15,000 pulses delivered in 2 d | Total of 15 sessions over 2 d (5 sessions, day 1 and 10 sessions, day 2) | Suicidal ideation | | One patient had increased suicidal ideation.  Four patients had prior psychiatric hospitalization, and 5 patients had a previous suicide attempt. |  |
| Keshtkar et al, 2011 | Randomized controlled trial | 73 patients with MDD | 40 ECT | 33 rTMS | rTMS | Left DLPFC: intensity was 90% of RMT, 408 stimulations/session for a total of 4,080 pulses in 10 d | 10 d | BDI suicidal subscore | | Both decreased the suicidal subscale score of BDI. However, ECT decreased it more significantly.  The decrease in HDRS was significantly greater in the ECT group than the rTMS group. |  |
| Levkovitz, et al, 2015 | Randomized controlled trial | 212 treatment-resistant MDD patients | 111 patients treated with active dTMS | 102 patients treated with sham | dTMS | Left DLPFC: stimulation intensity at 120% of MT, 18 Hz, 2-s pulse trains separated by 20-s intertrain intervals.  55 trains in each treatment session, for a total of 1,980 pulses/session | Acute treatment phase: 5 days/wk for 4 wk;  Maintenance phase: twice/ wk for 12 wk | Suicide attempt | | Two participants attempted suicide in the sham group. |  |
| O’Reardon et al, 2007 | Randomized controlled trial | Patients with MDD | 155 patients treated with rTMS | 146 patients treated with sham stimulation | rTMS | Left DLPFC: stimulation intensity: 120% of MT; stimulus frequency: 10 Hz, stimulus train duration of 4 s and intertrain interval of 26 s; total number of pulses/ session, 3,000 | TMS treatment was done over 4-6 wk | HDRS item 3 | | Suicidality was more common in the sham group (1.9% in the sham group and 0.6% in the treatment group). Exacerbation of suicidality occurred in 10 patients in the sham group and 1 patient in the treatment group.  Exacerbation of depression was more common in the sham group.  The baseline to wk 4 change was significantly higher in the treatment group than the sham group in both HDRS-17 and HDRS-24 scores (*P*=.006 and *P*=.01). Response and remission rates, according to the same parameters in addition to MADRS, were not significantly different between groups. |  |
| Petrosino et al, 2020 | Retrospective cohort study | 46 patients with PTSD | Active iTBS, 24 patients | Sham iTBS, 22 patients | iTBS | iTBS was applied to the right DLPFC, 1,800 pulses/session, 80% of RMT | Sham group: patients in the sham group were treated for a total of 10 sessions, 5 sessions/d for 2 wk  Active group: patients in the active group were treated for a total of 20 sessions, 5 sessions for 4 wk | Suicide completion | | There were no cases of completed suicide in either group during 1-year follow-up.  Clinical outcomes were better in the active-treatment group during the 1-year follow-up (*P*=.02). Relapse rate was lower and days to clinical relapse were longer in the active-treatment group.  There was no significant difference between groups regarding medication changes (addition, reduction, class switches, or other changes). |  |
| Rao et al, 2019 | Randomized sham-controlled pilot study | 34 patients with depression after TBI | Active rTMS: 17 | Sham rTMS: 17 | LFR-rTMS | 4 trains of 1 Hz, 300 pulses with 60 s of intertrain interval; total, 1,200 pulses/session. Stimulus intensity, 110% of RMT | Patients in both groups treated for 20 sessions over 4 wk; 5 sessions/wk | Suicidal ideation | | No significant difference was shown between groups regarding change in suicidal ideation (*P*=.40), as measured by SSI.  At baseline, there was no significant difference regarding number of suicide attempts (*P*=.41).  No significant difference was shown between groups regarding improvement in depression, as measured by HDRS (*P*=.79). |  |
| Valiengo et al, 2013 | Open-label crossover study and follow-up study of the SELECT TDCS trial (phase 1) | Phase II open-label crossover study | All 25 patients in phase II received active tDCS treatment | None in phase II or phase III | tDCS | Current density of 0.8 A/m^2^ (2 mA/25 cm^2^) per 30 min/d | 12 sessions for phase 3; maximum, 9 sessions for phase 3 every other wk for 3 mo and once/mo for 3 mo | Suicidal ideation/attempt | No suicidal ideation or attempts occurred in phase 2 or phase 3. | | |
| Wall et al, 2011 | Prospectıve open-label trial | 8 adolescents with TRD^e^ | 8 | None | rTMS | Left DLPFC: stimulation intensity was 120% of threshold; 10 Hz, 4-s trains, 26-s train interval for a total of 3,000 pulses for sessions | 30 sessions over 6-8 wk | Suicidal ideation | | Suicidal ideation was present at baseline for 3 patients but improved during treatment. |  |
| Wall et al, 2016 | Prospective open-label trial | 10 adolescents with TRD^f^ | 10 | None | rTMS | 10 Hz, 120% MT, 3,000 pulses applied to the left DLPFC | 30 sessions over 6-8 wk | Suicidal behavior | | During the trial, 2 participants had suicidal behaviors: 1, during an argument and 1, self-injurious behavior. |  |
| Weissman et al, 2018 | Randomized controlled trial | 156 patients with TRD | 56 unilateral, 52 bilateral rTMS | 48 sham | rTMS | First study: intensity, 100% of MT for patients <60 y; 120% of MT for patients >60 y. Left unilateral: 10 Hz; bilateral: left, 10 Hz, right, 1 Hz;  Second study: 120% of MT. Left unilateral: 10 Hz; bilateral: left, 10 Hz, right, 1 Hz | 15 sessions over 3 wk | Suicidal ideation | | Differences in the resolution of suicidal ideation, as measured by the suicide item of the HDRS, between the bilateral stimulation group and the sham group were significant (OR, 3.03 [95% CI 1.19-7.71]; *P*=.02).  The sham-stimulation group had significantly higher HDRS scores than the bilateral stimulation group at baseline (*P<*.05). |  |
| Yesavage et al, 2018 | Randomized controlled trial | 164 patients with TRD | 81 rTMS | 83 sham | rTMS | Left prefrontal:10 Hz, 120% MT, 4,000 pulses/session | 20-30 sessions; initial responders received another 6 sessions | Suicidal ideation | | Three patients in the active-treatment group and 4 patients in the sham group had suicidal ideation. |  |

Abbreviations: aiTBS, accelerated intermittent theta-burst stimulation; BDI-II, Beck Depression Inventory II; BSI, Beck Suicidal Intent Scale; CDRS-R, Children’s Depression Rating Scale–Revised; C-SSRS, Columbia Suicide Severity Rating Scale; cTBS, continuous theta-burst stimulation; DLPFC, dorsolateral prefrontal cortex; dTMS, deep transcranial magnetic stimulation; ECT, electroconvulsive therapy; HDRS, Hamilton Depression Rating Scale; iTBS, intermittent theta-burst stimulation; ITT, intent-to-treat; LFR-rTMS, low-frequency repetitive TMS; MADRS, Montgomery-Asberg Depression Rating Scale; MDD, major depressive disorder; MDE, major depressive episode; MT, motor threshold; NIBS, noninvasive brain stimulation; OR, odds ratio; PFC, prefrontal cortex; PTSD, posttraumatic stress disorder; RMT, resting motor threshold; rTMS, repetitive TMS; SELECT TDCS, Sertaline vs Electrical Current Therapy for Treating Depression Clinical Study; SSI, Suicidal Intent Scale; tDCS, transcranial direct-current stimulation; TMS, transcranial magnetic stimulation; TRD, treatment-resistant depression.

**Supplementary Table 2.** **ECT Studies**

| **Authors** | **Study design** | **Population** | **No. ECT** | **No. controls** | **ECT parameters** | **Treatment duration** | **Suicidality outcome/measure** | **Results** |
| --- | --- | --- | --- | --- | --- | --- | --- | --- |
| Ahmadi et al, 2016 | Retrospective case-control study | Patients with comorbid MDD and PTSD | 92 | 3,393 | Mean, 6±1 sessions | All patients received bifrontal ECT, with a stimulus dose at least 200% above the seizure threshold, based on an age-based method | Suicide completion | The rate of completed suicide was significantly lower in a group treated with ECT (2.2% vs 5.9%; *P<.*01).  The difference between the groups remained significant when only MDD patients were considered (RR, 0.28 [95% CI, 0.14-0.54]; *P*=.001). |
| Ahmadi et al, 2018 | Case series | Patients with MDD and patients with MDD and comorbid PTSD | 36 | None | All patients received bifrontal mECT, with a stimulus dose at least  200% above the seizure threshold, based on the age-based method, with a mean 14±1 ECT sessions | NA | Suicide completion | During 1-year follow-up after ECT treatment, there was no suicide in either group.  There were significant improvements in PTSD and MDD symptoms in both groups (*P*=.001). |
| Ambade et al, 2009 | Open-label trial | Depressive disorder as diagnosed by ICD-5 | 50 | 33 healthy controls | 5 times | NA | Suicidal ideation | ECT resulted in a significant decrease in suicidal ideation, as measured by SSI (*P*<.01). |
| Avery and Winokur, 1976 | Retrospective cohort study | Bipolar depression (DSM 1-2), psychotic depression, involutional melancholia,^a^ depressive neurosis,^b^ schizoaffective disorder | Only ECT, 135 patients;  ECT plus antidepressant, 122 patients | Adequate antidepressant, 71 patients;  Inadequate antidepressant, 121 patients;  Neither ECT nor antidepressant, 70 patients | NA | >5 sessions | Suicide completion | There were 8 completed suicides during the study: ECT, 1; adequate antidepressant, 1; ECT plus antidepressant, 3; inadequate antidepressant, 1; neither ECT nor antidepressant, 2.  No significant difference was shown between the total adequate treatment group (including patients treated with ECT, adequate antidepressant, or both) and the inadequate treatment group (including patients with adequate treatment and neither treatment) regarding number of completed suicides in 1-year or 3-year follow-up. |
| Avery and Winokur, 1978 | Retrospective cohort study | Bipolar depression (DSM 1-2), psychotic depression, involutional melancholia, depressive neurosis, schizoaffective disorder | Only ECT, 135 patients; ECT plus antidepressant, 122 patients | Adequate antidepressant, 71 patients; Inadequate antidepressant, 121 patients;  Neither ECT nor antidepressant, 71 patients | NA | NA | At the end of 1 year, there were 4 cases of completed suicides | No significant difference in the rate of attempted suicide between the groups treated with ECT (0.8%), antidepressants (4.2%), or adequate antidepressant treatment (7.0%).  During hospitalization and follow-up, 17 suicide attempts occurred. |
| Benzoni et al, 2015 | Case series | Bipolar disorder, type I and II major depressive episode (n=92, 34.8%);  Recurrent major depressive disorder (n=89, 33.7%);  Schizoaffective disorder (major depressive episode) (n=50, 18.9%);  Bipolar disorder, type I mixed episode (n=17, 6.4%);  Mood disorder with catatonic features (n=8, 3.3%);  Bipolar disorder, type I manic episode (n=5, 1.8%) | 264 patients | None | ECT was given 3times/wk. The number of sessions was decided based on the clinical course of the patient | Anesthesia was induced by thiopental (2 mg/kg) or etomidate (0.30 mg/kg). Succinyl choline was used for muscle relaxation (0.5-1.0 mg/kg), and patients were given prophylactic atropine to reduce bronchial secretions  ECT was given bitemporally using a brief-pulse administrator.  Wave width: 0.30 mA  Wave frequency: 20 Hz  Duration of stimulus: 4 sec | Suicidal ideation | Suicidal ideation decreased with ECT treatment among patients in all diagnostic categories. |
| Berg, 2010 | Open-label trial | Unipolar or bipolar depression | 20 | None | 6-13 sessions | NA | Suicide completion | Of 20 participants, 3 completed suicide. At the time of death, 2 of 3 participants had normal BDI.  There was no significant difference between suicide completers and survivors regarding number of previous suicide attempts. |
| Black et al, 1987 | Retrospective cohort study | MDD and schizoaffective disorder | 448 | Adequate antidepressant: 46  Inadequate antidepressant: 478  Neither treatment: 323 | NA | NA | Suicidal ideation and suicide attempts | Improvements in both suicidal ideation (*P*=.001, *P*=.01, and *P*=.01, respectively) and suicidal attempts (*P*=.001 for all 3 groups) were greater in the ECT than the other 3 groups.  Length of stay was longer for the ECT group. |
| Black et al, 1989 | Retrospective cohort study | Major affective disorder  (primary unipolar, 705 patients; Secondary unipolar, 219; bipolar depressive, 152) | 372 patients | Adequate antidepressant,^c^ 180 patients;  Inadequate antidepressant,^c^  317 patients;  Neither treatment, 207 patients | NA | 9 sessions | Suicide completion | No significant difference between groups was shown for completed suicide rates (*K*^2^: 0.944; *df*: 3).  33 suicides, 25 (69.4%) in the first 2 years of follow-up. |
| Bradvik and Berglund, 2000 | Retrospective case-control study | MDD with melancholic/  psychotic/catatonic features; melancholia, psychosis, catatonia, bipolar 1 disorder, bipolar 2 disorder | 89 patients with severe depression who completed suicide | 89 matched controls who did not complete suicide | Adequate ECT treatment was considered as a series of at least 6 treatments | The electric stimulus was manually regulated during each treatment to achieve an adequate grand mal seizure (>25 s), usually 0.6 to 0.9 amp; voltage, 180; unidirectional current; anesthetic, the ultra–short-acting barbiturate methohexital; no EEG registration performed | Suicide completion | The group who completed suicide and the matched controls were not significantly different regarding number of patients treated with ECT.  The percentage of patients who received maintenance treatment with antidepressants after ECT was significantly higher in the group who did not complete suicide (46% vs 13%; *P<*.025). |
| Bradvik and Berglund, 2006 | Retrospective case-control study | Primary severe depression | 96 patients with severe depression who completed suicide | 96 matched controls who did not complete suicide | A series of at least 6 treatments (given 3 times/ wk) was considered adequate | NA | Suicide attempts | Significantly fewer suicide attempts occurred in the ECT-treated group (χ^2^, 7.49; *P<*.01).  Patients in the ECT-treated group used less lethal means of suicide (3/12 vs 8/10, *P<.*02).  No significant differences were shown for number of episodes between the groups. |
| Cakir and Caglar, 2017 | Prospective cohort study | Unipolar or bipolar depression | 62 | None | ECT 3 times/wk  Number of treatments:  For unipolar depression:  remitted patients,  7.72±2.83;  nonremitted patients, 7.63±2.83;  Bipolar disorder:  remitted patients, 6.33±2.42;  nonremitted patients, 9.57±2.95 | The energy delivered was between 20% and 120%, and the charge delivered was between 108 and 648 mC | Suicide attempts | Patients were followed up for 1 year. Of 50 patients, 17 attempted suicide. One of these patients denied this attempt by saying it was part of an anger attack. |
| Ciapparelli et al, 2001 | Prospective cohort study | Mixed mania and bipolar depression | 64 | None | Number of mean ECT treatments was nearly equal in both groups (mixed mania, 7.2±1.7 vs bipolar depression, 7.3±1.6) (no significant differences between groups) | Pulse width: 1-2 ms (mean, 1.3 ms)  Frequency: 40-90 Hz (mean, 70 Hz)  Duration: 0.5-2.0 s (mean, 1.5 s)  Current: 0.55-0.80 A (mean, 0.70 A) | NA | After ECT, patients with mixed mania had a greater reduction in suicidality than patients with bipolar depression (*P<.*001), although significant improvement was shown for both groups (*P<.*001). |
| Dennis et al, 2017 | Retrospective cohort study | Unipolar depression, bipolar disorder (depressive, manic, mixed), schizophrenia, otherwise nonspecified mood disorders, psychotic disorders | Number of ECT treatments used for the calculation.  Number of total treatments, 166,711 | None | NA | NA | Suicide completion | Only 2 patients completed suicide within 14 days of the last ECT treatment. |
| Fazzino et al, 2013 | Prospective cohort study | MDD, bipolar disorder, bipolar disorder with psychotic features | 26 | None | 16 participants started the study while receiving acute ECT treatment (2 or 3 ECT treatments/wk), and 10 participants began the study during maintenance ECT (3 patients, 1 treatment/wk; 5 patients, 2/wk; and 2 patients, 3/wk) | 17 patients, Columbia protocol;  9 patients, Duke protocol | Suicide completion | Forty-one weekly reports (of 123 reports) from 14 patients (of 26 patients) showed suicidal ideation during patient follow-up via interactive voice response. |
| Hunt et al, 2011 | Retrospective case-control study | Affective disorders, 63; schizophrenia, 4; personality disorder, 2; anxiety disorder, 1 | 71 | 9,346 | NA | NA | Suicide completion | In the ECT-treated group, the mean number of completed suicides was 9, corresponding to a rate of 10.8 suicides/10,000 patients treated.  Affective disorder diagnosis was significantly higher in the ECT group (*P<.*01).  Inpatient treatment was significantly higher in the ECT group (*P<.*01). |
| Huston and Locher, 1948 | Retrospective cohort study | Manic depressive psychosis^d^ | 74 patients | 80 patients | 60 cycles, from 0.1 to 0.3 s; 80 to 150 V | Mean shocks, 7.2 | Suicide completion | The ECT group had a lower rate of completed suicide (6 patients or 7% of the control group and only 1 patient or 1% of the ECT group). |
| Huston and Locher, 1948 | Retrospective cohort study | Involutional psychosis^e^ | 61 patients | 93 patients | 60 cycles, from 0.1 to 0.3 s; 80 to 150 V | Mean shocks for patients who had only 1 series of 1 shock, 8;  Mean shocks for patients who had more than 1 series, 17 | Suicide completion | Only 1 patient in the ECT group completed suicide, whereas 12 patients (13%) completed suicide in the control group.  Completed suicides in the control group occurred within 2 years of patients’ disease onset but within 1 month of discharge in the ECT group. |
| Isometsä et al, 1996 | Case series | 1 patient with recurrent psychotic major depression and 1 patient with recurrent severe major depression | 2 | No control | Case 1: 2 unilateral and 4 bilateral treatments, none of which consistently resulted in convulsion lasting more than 25 s  Case 2: 5 unilateral treatments with consistent convulsions >40-55 s | NA | Suicide completion | Only 2 of 1,397 completed suicides had ECT during the previous year. Both patients died within 3 months of their last ECT treatment. |
| Jorgensen et al, 2020 | Retrospective cohort study | 92,895 patients with single or recurrent depression | 5,004 patients treated with ECT | None | NA | Median number of ECT sessions, 10 | Suicide completion and suicide attempt | 598 patients completed suicide and 3,923 patients attempted suicide in the cohort.  ECT was associated with increased risk of subsequent attempted and completed suicide. Adjustment for possible confounders decreased this risk, but it remained significant. Risk decreased with increased severity of depression.  In the ECT-treated group, 430 patients had a prior suicide attempt. The percentage of patients with a previous suicide attempt was not significantly different in the group treated with ECT than the non-ECT-treated group (95% CI, 0.96-1.11). |
| Kawoos et al, 2018 | Prospective cohort study | Affective disorders, 75%; schizophrenia, 10%; obsessive-compulsive disorder, 10%; and mental retardation, 5% | 40 | None | NA | NA | Suicidal ideation | ECT treatment resulted in significant improvement of suicidality for 5 patients with unipolar depression and suicidal ideation (*P<.*05). Suicidality in patients with obsessive-compulsive disorder also decreased significantly (*P<.*05). |
| Kellner et al, 2005 | Randomized controlled trial | Unipolar depression | 444 | None | ECT 3 times/wk; patients who had fewer than 10 ECT treatments were considered dropouts | A stimulus dose titration procedure was performed at the first treatment to determine seizure threshold, and subsequent stimulus dosing was 1.5 times the seizure threshold. Patients were treated with a standard brief-pulse ECT device with bitemporal electrode placement | Suicidal ideation | ECT treatment resulted in rapid relief of suicidal intent; 106 of 131 patients decreased to 0 intent. Older patients (>50 y) were 2.5 times more likely to have this decrease. A strong correlation was shown for decrease in depressive symptoms and suicide rating from baseline (r=.45, *P<.*001). There were 2 cases of completed suicide during the study. |
| Kellner et al, 2016 | Randomized controlled trial, phase 2 of PRIDE study | Unipolar MDD, no dementia, with or without psychosis  with a pretreatment HDRS score >21 | 61 | Medication-only group, antidepressant plus lithium: 59 | Seizure was accepted as adequate if it lasted more than 15 s;  Anesthesia included glycopyrrolate (0.2 mg IV), induction with methohexital (1 mg/kg), and muscle relaxation with succinylcholine (0.75 mg/kg) | 4 ECT treatments in 1 mo (1 treatment at the following times after randomization: 2-5 d, 7-12 d, 14-19 d, and 23-28 d) | Suicidal ideation | Three patients had suicidal ideation after ECT plus medication. None of these events were thought to be related to ECT. |
| Keshtkar et al, 2011 | Randomized controlled trial | MDD | 40 | 33 patients treated with rTMS | 10 sessions, 3 times/wk | Constant current (pulse width, 1.4 ms; duration, 1.25 s; frequency, 80 Hz; current, 0.8 amp) | BDI and HDRS suicide items | Both treatment modalities significantly decreased the suicidal subscale score of BDI (*P<.*001). ECT significantly decreased the suicide score compared with rTMS in the HDRS scale (*P<.*001). |
| Krepela et al, 2019 | Retrospective cohort study | 19 patients with pharmaco-resistant schizophrenia | 19 patients | None | NA | NA | Suicidal ideation | ECT was successful in treating suicidal ideation in 3 patients.  ECT was successful in treating agitation in 14 patients and in treating self-harm in 5 patients. |
| Liang et al, 2018 | Retrospective cohort study | Unipolar and bipolar disorder | 487 patients | 1,948 patients treated with psycho-pharmacotherapy | NA | NA | Suicide completion | Patients in the ECT group had lower completed suicide rates by 19.7%. This difference was more significant in the unipolar depression group (HR, 0.79; *P*=.041) than the bipolar disorder group (HR, 0.923; *P*=.25).  Of the non-ECT group, 88.2% completed suicide in the first year, whereas 48.6% in the ECT group completed suicide. |
| Milstein et al, 1986 | Case-control study | 76 patients who committed suicide during hospitalization | 76 | 76 | NA | NA | Suicide completion | A history of ECT did not differ significantly in patients who completed suicide vs patients who died of other causes. Sixteen patients who had ECT at their first hospitalization or had a history of ECT died of suicide. |
| Mitchell et al, 2018 | Retrospective cohort study | MDD, bipolar disorder, and catatonia | 25 patients | None | NA | NA | NA | At 6-month follow-up from final ECT, 78.3% of the patients reported mild or no suicidality as measured by About My Life Assessment. |
| Munk-Olsen et al, 2007 | Retrospective cohort study | Deceased inpatient population, including schizophrenia, schizoaffective disorder, bipolar disorder, unipolar depressive disorder, other nonaffective psychoses, and other disorders | 783 patients | 5,781 patients | NA | NA | Suicide completion | The completed suicide rate was higher in a group who were treated with ECT, although the result was not significant (RR, 1.20 [95% CI, 0.99-1.47]).  When only the first 7 from the previous ECT were considered, the difference became significant (RR, 4.82 [95% CI, 2.12-10.95]). At 4 weeks after the final ECT treatment, the difference was no longer significant. |
| Nordenskjold et al, 2011 | Retrospective cohort study | MDD | 486 patients | None | The mean dose at the last unilateral treatment was 0.46±0.10 ms, 75±25 Hz, 7.5±0.9 s, 833±50 mA, and 429±177 mC | The mean number of ECT sessions was 7.9±3.0  For patients who had maintenance treatment, the mean number of maintenance sessions was 12±15 | Suicide completion | During 1-year follow-up, there were 9 completed suicides (2%). |
| Nordenskjold et al, 2013 | Randomized controlled trial | Depression (bipolar depression, depression with melancholic features, depression with psychotic symptoms) | ECT and pharmacotherapy, 28 patients | Pharmacotherapy only, 28 patients | Patients in the ECT group had 29 total ECT treatments over 1 y | The mean electrical dosage during continuation ECT was 0.36±0.11 ms, 74±20 Hz, 6.40±1.21 s, 813±35 mA, and 292±166 mC, preferred epileptic activity over 20 s | Suicidal ideation and suicide attempt | In the group with pharmacotherapy alone, 3 suicide attempts and 1 case of suicidal ideation occurred; in the combined ECT and pharmacotherapy group, there was only 1 case of ambiguous suicide attempt. |
| Nuttall et al, 2004 | Retrospective cohort study | NA | 2,279 patients | None | Sodium pentothal was most commonly used for anesthesia  Succinyl choline was used for muscle relaxation | The technique used was consistent with that described in the guidelines of the American Psychiatric Association Committee on ECT | Suicide completion | Two patients completed suicide 4 and 17 days after their last treatment. |
| Patel et al, 2006 | Matched case-control study | Bipolar disorder, MDD, schizoaffective disorder, and disorders with possible substance abuse | 30 | 30 | 3 times/wk for 5 to 10 treatments (mode, 8 treatments) | NA | Suicidal ideation | Significant improvement in suicidal intent was shown compared with the group using psychotropic medications (t_28_=9.79, *P<.*001). |
| Popiolek et al, 2018 | Retrospective cohort study | Bipolar depression | 1,255 patients | None | Mean of 7.8±4.1 index ECT sessions (847 patients) | Mean charge, 422±195 mC (566 patients); mean current, 840.9±61.8 mA (n=554); mean duration, 7.1±1.3 s (n=553); mean frequency, 68.9±21.2 Hz (n=560); mean pulse width, 0.52±0.16 ms (n=566); mean electroencephalogram seizure duration, 36.5±17.8 s (n=534) | Suicide completion and suicide attempt | During mean follow-up (1 y), 17 patients completed suicide, corresponding to a 0.9% suicide rate in the study population.  In addition, 47 patients had attempted suicide. Ten of these patients completed suicide later during follow-up. |
| Ray-Griffith et al, 2016 | Case series | Depression with suicidal ideation, 2 patients; depression without suicidal ideation, 3 patients; mood disorder with suicidal ideation, 1 patient; mixed phase of bipolar illness with suicidal ideation, 1 patient; and depressed phase of bipolar illness with suicidal ideation, 1 patient | 8 | None | Maximum of 3 ECT treatments were administered each wk (30 total ECT for the 8 patients) | Brief-pulse current of 800 mA. All treatments were performed with right unilateral electrode placement. Patients were monitored with EEG during and after stimulation. Seizure activity was confirmed centrally and peripherally using EEG | Suicidal ideation | Five patients improved with respect to suicidal ideation. |
| Reid et al, 1998 | Retrospective cohort study | Mood disorder, schizophrenia, schizoaffective, and other disorders | 2,853 patients | None | Mean number of ECT treatments, 5.9 | Electrodes were placed bilaterally in 73.2% of cases and unilaterally in 18.6% of cases | Suicide completion | Only 2 suicides occurred within 14 days of the last ECT treatment. |
| Rich et al, 1986 | Open-label trial | Depression | 37 | None | NA | NA | Suicidal ideation | Suicidal ideation decreased after ECT treatment.  Improvement in suicidal ideation occurred earlier than improvement in low energy (*P<.*05). |
| Ronnqvist et al, 2019 | Case-control study | Patients with postpartum depression | 180 patients with postpartum depression | 180 matched controls (treated with ECT but no postpartum depression) | NA | Propofol or thiopental used as an anesthetic;  92.4% of patients had unilateral treatment | Suicide completion | Among 102 patients who relapsed, 101 were hospitalized, and 1 completed suicide.  In the postpartum depression group, 73 patients relapsed, and none completed suicide. |
| Rosa et al, 2006 | Randomized single-blind study | Unipolar nonpsychotic refractory depression | 15 patients in the ECT group | 15 patients in the TMS group | ECT was performed according to institutional standards and guidelines of the  American Psychiatric Association;  Anesthesia:  100% oxygen during ECT sessions; etomidate (1.0-1.5 mg/kg IV);  Muscle relaxation:  succinylcholine (0.5-1.25 mg/kg IV); atropine (0.4-1.0 mg IV) | ECT occurred over 4 wk unless a patient significantly improved clinically  TMS occurred over 4 wk | Suicide attempt | Two suicide attempts occurred in both groups.  No significant difference shown between groups regarding improvement in depression and change in neuropsychological function. |
| Sharma, 1999 | Retrospective case-control study | 45 inpatients: MDD, 19; bipolar disorder, 12; schizophrenia, 6; schizoaffective disorder, 9; other disorders, 4 | 45 psychiatric inpatients who completed suicide | 45 controls matched for age, sex, and diagnosis who did not complete suicide | Mean number of ECT treatments of the group who committed suicide, 9.7±7.7; nonsuicide group, 31±29.7 | NA | Suicide completion | In the suicide group, 7 patients had ECT treatment. In the nonsuicide group, 2 patients had ECT treatment.  No significant difference was shown for ECT between groups.  The control group had a longer, but not significant time, between ECT and suicide/discharge (*P*=.08) and more ECT treatment at the index admission (*P*=.09). |
| Shilton et al, 2020 | Case series | 30 hospitalized adolescent female patients with anorexia nervosa treated with ECT due to their TRD and/or severe suicidal risk | 30 patients with anorexia nervosa with TRD and/or severe suicidal risk | None | NA | ECT was given according to standard protocol | Suicidal ideation or suicide attempts | Of 30 patients, 27 were treated with ECT because of increased suicidal risk (ie, severe suicidal ideation or suicide attempt).  During ECT treatment, there was only 1 suicide attempt.  Following ECT treatment, there were no cases of attempted suicide.  There was a significant decrease in depression severity after vs before ECT, as measured by CGI-S.  There were 17 cases of previous suicide attempts. |
| Shiwach et al, 2001^7^ | Retrospective cohort study | NA | 8,148 patients who received 49,048 ECT treatments | None | NA | NA | Suicide completion | Only 6 patients completed suicide within 14 days of the last treatment. |
| Sun et al, 2016 | Open-label trial | 27 patients with TRD | 27 patients treated with MST | None | Methohexital sodium, methohexital  with remifentanil hydrochloride, and  ketamine hydrochloride used as anesthetic  agents;  Muscle relaxation: succinylcholine;  Stimulation: over the frontal cortex (midline) directly over the Fz electrode, performed per international 10-20 system;  Patients’ mean seizure duration: 45.1±21.4 s | Patients were treated for 24 sessions or until their depression remitted according to HDRS | Suicidal ideation | There was a significant difference between pre- and post-MST SSI scores (*P*=.001).  The N100 and LICI in the frontal cortex were indicators of remission of suicidal ideation (*P*=.003). |
| Sun et al, 2018 | Open-label trial | 23 patients with TRD | 23 patients treated with MST | None | NA | Patients were treated for 24 sessions or until their depression remitted according to HDRS | Suicidal ideation | A significant decrease in suicidal ideation occurred, as measured by SSI (*P<.*001). Of 18 patients with suicidal ideation at baseline, suicidal ideation resolved for 8; 10 continued to have suicidal ideation.  No signifiicant relationship existed between change in cortical-evoked activity in frontal and central electrodes and a decrease in SSI scores.  A significant correlation existed between decreased LICI scores over the frontal and central electrodes and decreased SSI scores after MST treatment (*P*=.04). Relevant to this, there was a significant decrease in LICI when the analysis was limited to the group with resolved suicidal ideation (*P*=.048). |
| Tsuang et al, 1979 | Retrospective cohort study | Schizoaffective disorder | 24 patients | 50 patients | NA | NA | Suicide completion | No significant differences were shown for completed suicide cases of patients who had ECT vs those who did not (*P*=.30). |
| Veltman et al, 2019 | Prospective cohort study | MDD | 110 patients over the age of 55 diagnosed with MDD and referred for ECT | None | 2 times/wk; treatment continued until patients had a MADRS score <10 for 2 consecutive ratings with a wk interval | Unilateral; if no improvement was seen after 6 wk or the clinical condition deteriorated, bilateral ECT was used | NA | The decrease in suicidality was significant only for a week.  The decrease in depressive and melancholic dimensions were more significant than a decrease in suicidality at the end of 2 weeks. |
| Youseff, 1990 | Retrospective cohort study | Major depression, 12; bipolar disorder, 6; schizophrenic disorder, 3; schizoaffective disorder, 2; adjustment disorder, 3; alcohol dependence, 3; personality disorder, 1 | 30 patients who completed suicide | None | Mean number of recent ECT treatments in 19 patients, 5.7 | NA | Suicide completion | Among 30 patients who completed suicide, 19 had recent ECT treatment before suicide. |
| Youssef et al, 2019 | Double-blind, randomized, controlled trial | 7 patients with major depressive episode (unipolar or bipolar disorder) | 3 patients were treated with LAP-ST | 4 patients were treated with standard RUL | Mean sessions for the LAP-ST group: 6.3; mean sessions for standard RUL ECT group: 6 | In the LAP-ST group, pulse width was 0.3 ms, and initial amplitude was 500 mA in the first session. In standard RUL, ECT arm pulse width was 0.3 ms, and initial amplitude was 900 mA in the first session. Stimulus amplitude was titrated in subsequent sessions  Anesthesia was methohexital induction and succinylcholine as a muscle relaxant | MADRS item 2 | In the LAP-ST group, suicidality remitted by session 3; by session 4, all patients in both groups had remission. Suicidality was evaluated with MADRS item 2.  All patients had active suicidality at baseline. Mean suicidality at baseline was higher in the LAP-ST group than the standard RUL ECT group. |

Abbreviations: BDI, Beck Depression Inventory; CGI-S, Clinical Global Impression Severity of Illness; *df*, degrees of freedom; DSM, *Diagnostic and Statistical Manual of Mental Disorders;* ECT, electroconvulsive therapy; EEG, electoencephalogram; HDRS, Hamilton Depression Rating Scale; HR, hazard ratio; ICD-5, *International Classification of Diseases 5*; IV, intravenous; LAP-ST, low-amplitude seizure therapy; LICI, long-interval intracortical inhibition; MADRS, Montgomery-Asberg Depression Rating Scale; MDD, major depressive disorder; mECT, modified electroconvulsive therapy; MST, magnetic seizure therapy; NA, not applicable; PRIDE, Population Research in Identities and Disparities for Equality; PTSD, posttraumatic stress disorder; RR, relative risk; rTMS, repetitive transcranial magnetic stimulation; RUL, right unilateral; RUL ECT, right unilateral ECT; SSI, Suicidal Intent Scale; TMS, transcranial magnetic stimulation; TRD, treatment-resistant depression.

^a^ Involutional melancholia is defined as “depression of gradual onset occurring during the involutional years (40-55 in women and 50-65 in men) with symptoms of marked anxiety, agitation, restlessness, somatic concerns, hypochondriasis, occasional somatic or nihilistic delusions, insomnia, anorexia, and weight loss.” It has not been recognized as a psychiatric disorder in DSM-5.^42^

^b^ Depressive neurosis or neurotic depression is defined as^42^ “depression in an emotionally unstable person.” This category includes depression secondary to major personality disorders, neuroses, and drug use disorders.

^c^ Adequate antidepressant group in this study refers to the minimum 4 wk of antidepressant therapy with at least 2 wk of imipramine (150 mg/d) or its equivalent. Inadequate group refers to less time or lower dose with the treatment.

^d^ Manic depressive psychosis in the referenced paper is used for psychosis in which affect is the primary psychopathology.

^e^ Involutional psychosis is defined as a^42^ “psychosis occurring in middle life and the following years, without evidence of either organic intellectual defects or a previous affective disorder.”

**Supplementary Table 3.** **VNS Studies**

| **Authors** | **Study design** | **Population** | **No. VNS** | **No. control** | **VNS parameters** | **Suicidality outcome/measure** |
| --- | --- | --- | --- | --- | --- | --- |
| Aaronson et al, 2017 | Open-label, nonrandomized, observational registry | 765 patients with TRD | 489 patients with adjunctive VNS treatment | 276 | NA | A statistically significant difference existed for QIDS-SR item 12 (OR, 2.11 [95% CI, 1.28-3.48]; *P*=.04) and for the investigator-completed suicidality assessment (OR, 2.04 [95% CI, 1.08-3.86]; *P*=.03) but not for MADRS item 10 (OR, 1.67 [95% CI, 0.98-2.83]; *P* =.058).  The mean lifetime number of attempted suicides was 1.8±4.0 in the VNS-treated group and 1.2±2.4 in the treatment-as-usual group. |
| Aaronson et al, 2013 | Randomized, controlled, double-blind study | 310 patients with TRD | High: 107  Medium:102 | Low: 101 | Low: 0.25 mA, pulse width, 130 µs;  Medium: 0.5-1.0 mA, 250 µs;  High: 1.25-1.5 mA, 250 µs;  All treatment groups had the same frequency (20 Hz) and same duty cycles (30 s on, 5 s off) | No differences were shown for psychiatric history and rate of suicide attempts.  Suicide attempts were higher in the low group than the combined medium and high group (*P*=.06).  There were 2 completed suicides: 1 in the low group (the patient had a history of 2 suicide attempts) and 1 in the high group (no history of suicide attempt).  There were no significant differences between groups in IDS-C scores in remission or response rates at the end of 22 weeks for any of the scales and between groups in long-term follow-up. |
| Bajbouj et al, 2010 | Open-label, nonrandomized, longitudinal study | 74 patients with TRD;  70 patients in long-term follow-up;  60 patients with follow-up at 12 mo;  49 patients with follow-up at 24 mo | 74 | NA | NA | There were 2 suicide attempts and 39 serious adverse events.  There were 2 completed suicides within the first year of the study; 1 responded, and 1 did not respond to VNS treatment.  Rate of completed suicide (3%) was higher than expected for patients with TRD. |
| Nahas et al, 2005 | Open-label pilot study | 59 adult outpatients with MDD and bipolar disorder with TRD | 59 adult outpatients with MDD and bipolar disorder with TRD | None | NA | There were 3 suicide attempts.  There was significant remission and response at 3 months but no difference between 3, 12, and 24 months. |
| Olin et al, 2012 | Open-label, nonrandomized, longitudinal study | In intent-to-treat analysis, 636 patients had TRD | 335 patients, VNS plus treatment-as-usual | 301 patients, treatment-as-usual | NA | No significant differences in suicidal ideation were shown between the VNS plus treatment-as-usual and treatment-as-usual groups, as assessed both by AOS (RR, 1.30 [95% CI, 0.79-2.14]) and MADRS (RR, 0.89 [95% CI, 0.54-1.48]) scores. When evaluated with a marginal structural model, the difference was significant in MADRS assessment (RR, 0.80 [95% CI, 0.68-0.95]).  The difference in completed suicide rates was not significantly different between the 2 groups, even though in VNS-adjunctive groups, the numbers were nearly half of those in the treatment-as-usual group (0.88 vs 1.61/1,000 person-years).  There were 2.1±4.4 lifetime suicide attempts in the adjunctive VNS group and 1.2±2.4 suicide attempts in the treatment-as-usual group. |
| Rush et al, 2005 | Prospective cohort study | 205 outpatients with MDD (185) and bipolar disorder (20) with TRD | 110 patients: active stimulation during the acute phase of the study (3 mo) | 95 patients: sham stimulation during the acute phase of the study (3 mo) | NA | One patient in the active VNS group completed suicide 5 weeks after stimulation. This patient had a prior suicide attempt and had been hospitalized 3 times.  In the first 3 months, 2 patients had 2 suicide attempts, and 5 patients had 9 suicide attempts over the subsequent 9 months of the study.  Of 7 patients, 4 had a history of at least 1 suicide attempt.  Worsening depression was responsible for 30 hospitalizations; 24 of these patients (80%) had prior suicide attempts.  Improvement in the active-treatment group was significantly higher than in the sham-treatment group (*P*=.002). |
| Rush et al, 2005 | Randomized controlled study | 235 patients with TRD: MDD, 210; bipolar disorder, 25 | 112 patients treated with active stimulation | 110 patients treated with sham stimulation | Study lasted 10 wk | No significant differences existed between remission rates (*P*=.25). The active-treatment group had significantly higher response rates than the sham group (*P*=.03).  One patient committed suicide within 5 weeks of active treatment. |
| Schlaepfer et al, 2008 | Prospective cohort study | 74 patients with TRD | 74 patients with TRD | None | 0.25 mA; pulse frequency,  20 Hz; pulse width, 500 ms (stimulation on for 30 s and off for 5 min). Output current  increased during stimulation-adjustment period in 0.25 mA increments until a maximal tolerable and comfortable level was achieved | During the 1-year study, 2 patients completed suicide, and there was 1 suicide attempt. |

Abbreviations: IDS-C, Inventory of Depressive Symptamotology; MADRS, Montgomery-Asberg Depression Rating Scale; MDD, major depressive disorder; NA, not applicable; OR, odds ratio; QIDS-SR, Quick Inventory of Depressive Symptomatology–Self-Report; RR, relative risk; TRD, treatment-resistant depression; VNS, vagal nerve stimulation.

**Supplementary Table 4.** **DBS Studies**

| **Authors** | **Study design** | **Patient population** | **No. DBS** | **No. control** | **DBS parameters** | **Suicidality outcome/measure** | **Results** |
| --- | --- | --- | --- | --- | --- | --- | --- |
| Antonini, 2007 | Prospective cohort study | 150 patients with Parkinson disease | 150 | None | STN, target | Suicide attempts | Four patients attempted suicide. |
| Bergfeld et al, 2016 | Randomized, double-blind, crossover trial | 25 patients with TRD | 25 | None | Applied to vALIC:  Amplitude: 2.5-6.0 V;  Pulse width and frequency: 90 µs and 130 or 180 Hz, respectively;  2 blocks/wk, 6 wk of treatment | Suicide attempts, suicide completion and suicidal ideation | During optimization phase: 4 patients, 5 suicide attempts (n=25).  Increase in suicidal ideation: 2 patients (1 nonresponder and 1 responder who was a nonresponder at the time of this adverse event); 1, sham DBS; 1, active DBS (n=16).  Two patients (both nonresponders) died of suicide after withdrawal from the study and after DBS was discontinued (1 from suicide, 1 by euthanasia) (n=16).  Before surgery, 7 patients had previous suicide attempts (mean, 3.3±1.7).  Mean duration of the optimization phase was 51.6±22.0 weeks. |
| Berney et al, 2002 | Case series | 24 patients with Parkinson disease | 24 | None | Bilateral STN was the target. Other treatment parameters were unavailable | 3 of 24 patients became suicidal.  The authors did not specify suicidal ideation, intent, or attempt | Of the patients, 6 had significant worsening of mood. Among these 6, 3 became suicidal.  No significant difference existed in motor improvement between depressed  and nondepressed subgroups.  Reduction of anti-Parkinson medication in levodopa equivalents was similar between the 2 subgroups. |
| Bewernick et al, 2012 | Prospective cohort study | 11 patients with TRD | 11 | None | DBS applied bilaterally to nucleus accumbens;  Most patients were stimulated at 5-8 V, 90 µs with 130 Hz frequency, monopolar | Suicide completion and suicide attempts | During 12-month follow-up, 1 patient completed suicide, and 1 patient attempted suicide. Both were nonresponders. |
| Børretzen et al, 2014 | Retrospective cohort study | 46 patients with essential tremor treated with DBS | 46 | None | 6 patients, bilateral stimulation of ventral intermediate thalamic nucleus;  Treatment parameters (postoperative/follow-up)  Voltage: 2.3/3.5 V  Pulse width: 90 µs (60-120)  Pulse frequency: 148 Hz (90-230)/181 (130-230) | Suicide completions | One patient completed suicide 7 months after surgery. |
| Burkhard et al, 2004 | Prospective cohort study | 140 patients with movement disorders | 140 | None | DBS was bilateral in all patients  Depending on the treated condition, targets were the motor nucleus of thalamus, GPi, or STN | Suicide completions | Six of 140 patients completed suicide (4.3%).  Death by suicide occurred at 3.1 years (range, 4 mo-7 y) after last DBS surgery.  History of severe depression and multiple successive surgeries were major risk factors.  No relationships were found for the underlying condition, DBS target, electrical parameters, or modifications of treatment. |
| Castelli et al, 2006 | Prospective cohort study | 72 patients with Parkinson disease | 72 | None | Target: bilateral STN;  Right STN: voltage: 3.3±0.4 V; pulse width: 67.5±13.1 µs; pulse frequency: 143.1±19.9 Hz;  Left STN: voltage: 3.3±0.3 V; pulse width: 68.5±19.3 µs; pulse frequency: 142.7±22.2 Hz | Suicidal ideation | No significant change in suicidal ideation (preoperative, 0.16±0.45 vs postoperative, 0.14±0.39; *P*=.83), as measured by BDI item 9.  During the study, STN stimulation resulted in a significant reduction in medication use (*P<.*001). |
| Chopra et al, 2014 | Prospective cohort study | 54 patients with Parkinson disease | 54 | None | Of the patients, 51 had STN stimulation; 3, bilateral GPi stimulation  Treatment parameters were unavailable | Suicide attempts or suicide completions | No attempted or completed suicide. |
| Crowell et al, 2019 | Open-label trial with long-term follow-up design | 28 patients (20 with MDD; 8, bipolar II disorder) with TRD | 28 | None | All patients were treated with subcallosal cingulate DBS  Stimulation parameters:  130 Hz, 90 µs pulse width, 4-8 mA | Suicidal ideation and suicide attempts | There were 8 cases of suicidal ideation and 5 cases of suicide attempt during 6-year follow-up. The 5 suicide attempts corresponded to 3/100 patient-years.  There were 17 previous suicide attempts in the MDD group and 15 suicide attempts in the bipolar II disorder group. |
| Diestro et al, 2018 | Retrospective case series | 17 patients with Parkinson disease | 17 | None | Targets were either STN or GPi  Amplitude of the stimulation: between 3.5-4 V; pulse width, 60 µs and pulse frequency, 180 Hz | Suicide attempts | One patient attempted suicide 5 months after surgery. This patient had never attempted suicide before. |
| Dougherty et al, 2015 | Randomized blinded study with an open-label continuation phase | 29 patients with TRD | 15 active-treatment patients | 14 sham-control patients | The bilateral ventral capsule and striatum were targeted  For both monopolar and bipolar survey, all 8 electrodes were tested at increasing amplitudes at 2 different pulse widths  If the undesirable stimulation effect was produced at less than 8 V, then electrodes were not tested for higher amplitudes | Suicidal ideation and suicide attempts | During the blinded phase, suicidal ideation was more common in the active-treatment group. There were 2 cases of suicidal ideation in the active group and no cases in the sham group.  During the entire study, there were 5 cases of suicidal ideation, 4 cases of suicide attempt, and 1 completed suicide. |
| Follett et al, 2010 | Randomized blinded study | 299 Parkinson disease patients | 152 patients with pallidal stimulation | 147 patients with subthalamic stimulation | Bilateral STN or GPi-DBS; Treatment parameters were unavailable | Suicidal ideation and suicide completions | No significant differences existed (*P*=.99) between the STN and GPi groups regarding suicidal ideation, although depression worsened in the STN group and improved in the GPi group.  In the group receiving subthalamic stimulation, 2 patients attempted suicide, and 1 had increased suicidal ideation.  In the GPi group, 1 patient completed suicide. |
| Foncke et al, 2006 | Prospective cohort study | 16 patients with dystonia | 16 | None | Bilateral GPi were targeted | Suicide completions | Two patients completed suicide during follow-up (3 wk and 14 mo).  1 of the patients had a history of 2 periods of antidepressant-treated depression (first episode treated with amitriptyline and second episode with paroxetine).  The second patient had a history of 2 mild depressive episodes treated with paroxetine and a history of childhood anxiety and social phobia. His dystonia significantly improved after surgery, and he was functional and reported to be happy. |
| Funkiewiez et al, 2004 | Retrospective cohort study | 77 patients with Parkinson disease | 77 | None | Bilateral STN was the target  Treatment parameters were unavailable | Suicide completion | 1 patient completed suicide (36 mo), and 4 patients had a suicide attempt (2, 3, 5, and 6 mo).  1 of the patients had worsening in mood after discontinuing the dopaminergic treatment.  Another patient had a sudden and worsening of mood with bursts of crying as DBS was deactivated. |
| Gan et al, 2007 | Prospective cohort study | 36 patients with Parkinson disease | 36 patients treated with STN-DBS | None | Bilateral STNs targeted  Mean stimulation parameters were 3.1±0.4 V, 66.7±12.5 µs, 147.3±20 Hz | Suicide completion | At the end of 3-year follow-up, only 1 patient completed suicide. This patient had pneumonia 1 day after surgery and did not recover from the Parkinson symptoms. |
| Gervais-Bernard et al, 2009 | Prospective cohort study | 42 patients with Parkinson disease | 42 patients treated with STN-DBS | None | Bilateral STN-DBS applied  Mean stimulation parameters for 5 y: voltage, 3.8±4.4 V; pulse width, 67.8±13.2 µs; pulse frequency, 139.1±23.3 Hz | Suicide completion | During 5-year follow-up, 1 patient completed suicide. This patient had pneumonia and atrial fibrillation and was never able to recover fully from Parkinson symptoms. |
| Giannini et al, 2019 | Case-control study | 534 patients with Parkinson disease | 26 patients completed or attempted suicide | 32 control patients who did not attempt or complete suicide | All patients had STN-DBS  Parameters were unavailable | Suicide completion and suicide attempts | The mean follow-up was 9.1±5.9 years.  Four patients completed suicide (0.7%); 22 attempted suicide (4.0%). All suicide attempts were in unique individuals.  The suicide rate in the first postoperative year was 187.2/100,000. The overall median latency to completed or attempted suicide was 2.14 years (range, 0.15-8.51 y).  History of suicidal ideation and of suicide attempts were significantly higher in the group who attempted or completed suicide (*P<.*001). Psychotic symptoms were also more common in this group than in the control group.  The group of patients who completed or attempted suicide had more frequent history of depression, anxiety, and manic episodes than the control group, although the difference was not significant. Nevertheless, suicide completers had higher rates of these psychiatric disorders than suicide attempters (*P*=.69, .16, and .20, respectively).  There was no significant difference between groups regarding improvement in Parkinson symptoms, as measured by UPDRS III score.  A family history of psychiatric disorders, especially addiction, depression, and suicide was higher in the completed suicide/suicide attempt group (*P*=.04, *P*=.03 and *P*=.01).  The suicide completer/attempter group had a higher rate of psychotropic treatment in both the pre- and postoperative periods (*P*=.03 and *P*=.01, respectively). |
| Holtzheimer et al, 2012 | Single-blind, sham-stimulation phase followed by 24-wk open-label stimulation phase, followed by single-blind discontinuation phase and follow-up | 17 patients with treatment-resistant bipolar (n=7) and unipolar depression (n=10) | 10 patients with unipolar TRD, 7 patients with bipolar TRD | None | Bilateral SCC-DBS  Initial stimulation parameters: pulse amplitude, 4 mA; pulse width, 91 µs; pulse frequency, 130 Hz  If there was no improvement (<10% decrease in HDRS) in 1 wk, stimulus amplitude was gradually increased by 1 mA until 8 mA reached | Suicidal ideation and suicide attempts | During the discontinuation phase, suicidal ideation increased in 3 patients. In all 3 cases, suicidal ideation was temporally associated with reinitiation, as discontinuation had resulted in full recurrence of depressive symptoms.  There were 2 suicide attempts during the study: 1 within the first week of active stimulation, which resolved without any stimulation change, and 1 in the observational phase, which also resolved without further adjustment of parameters. |
| Holtzheimer et al, 2017 | Prospective, randomized, double-blind plus open-label sham-controlled study | 90 patients with TRD | 60 active-treatment patients | 30 sham-treated patients | Bilateral subcallosal cingulate gyrus was stimulated: 130 mHz and 4 mA applied with 91 µs pulse width. If MADRS scores improved less than 10%, pulse amplitude was increased to 6 mA; if still less than 10%, amplitude was increased to 8 mA | Suicidal ideation and suicide attempts | During the randomized double-blind phase (6 mo), 1 patient had suicidal ideation and 1 patient attempted suicide (both in the stimulation group).  During the open-label phase (6-12 mo), 1 patient had suicidal ideation and 1 patient attempted suicide in the stimulation group; 2 patients completed suicide in the sham group.^a^  No difference existed in response or remission as measured by MADRS at 6 months.  At 12 months, the proportion of patients with response or remission did not change significantly compared with 6 months.  Suicide attempts, except for 1, were at least 6 months after surgery.  History of suicide attempts was 38% in the stimulation group and 30% in the control group. |
| Houeto et al, 2002 | Retrospective cohort study | 24 patients with Parkinson disease | 24 patients with Parkinson disease | None | Bilateral STN-DBS  Treatment parameters: voltage, 2.75± 0.4 V; pulse width, 60 µs; pulse frequency, 144±27 Hz | MINI items | Three patients already had suicidal risk before surgery, and 4 patients had suicidal risk after surgery, as measured by the MINI interview. |
| Huff et al, 2010 | Double-blind, sham-controlled, crossover trial | Patients with treatment-resistant obsessive-compulsive disorder | 10 | None | Unilateral nucleus accumbens stimulation  Treatment parameters: amplitude, 4.5 V; pulse width, 90 µs; pulse frequency, 135 Hz | Suicidal ideation | One patient with a history of suicidal ideation had suicidal ideation after 6 months. After modifying her medication, the suicidal ideation disappeared. |
| Kennedy et al, 2011 | Prospective cohort study | 20 patients with TRD | 20 | None | Bilateral subcallosal cingulate area was stimulated: voltage, 9 V; pulse width, 60 µs; pulse frequency, 130 Hz | Suicidal ideation and suicide completions | During follow-up, 3 patients were hospitalized for suicidal ideation.  There were 2 cases of completed suicide, 1 at 35 months after surgery and 1 at 75 months after surgery.  During follow-up, 3 patients were hospitalized for worsening depression.  Suicide completers were different than suicide attempters in prolonged hospitalization and lack of integration into the workforce.  Both suicide attempters and completers had family histories of psychiatric disorders; except for 1 patient, all had at least 1 previous suicide attempt. |
| Kenney et al, 2007 | Retrospective cohort study | Patients with movement disorders | 319 patients with movement disorders;  Parkinson disease: 182 patients;  Essential tremor: 112 patients;  Dystonia: 19 patients;  Other hyperkinetic movement: 6 patients | None | VIM-DBS, STN-DBS, and GPi-DBS applied  Treatment parameters were unavailable | Suicide completion | One patient treated with STN-DBS completed suicide. |
| Kleiner-Fisman et al, 2003 | Prospective cohort study | Patients with advanced Parkinson disease | 25 patients with advanced Parkinson disease | None | Bilateral STN-DBS applied  Treatment parameters were unavailable | Suicide attempts | One patient without symptoms of depression attempted suicide. |
| Krack et al, 2003 | Prospective cohort study | Patients with advanced Parkinson disease | 49 patients with advanced Parkinson disease | None | Bilateral STN-DBS applied  Treatment parameters were unavailable | Suicide completion and suicide attempts | One patient who was suicidal before surgery completed suicide after surgery.  There were 3 suicide attempts. |
| Lhommee et al, 2012 | Prospective cohort study | 63 patients with Parkinson disease | 63 | None | Target was STN  Treatment parameters unavailable | Suicide attempts | Two patients attempted suicide, which corresponded to 3% suicide rate.  One patient attempted suicide after device infection and the other at 2 months after surgery. He was irritable at that time but never met criteria for MDE. |
| Lozano et al, 2012 | Prospective open-label trial | Patients with TRD | 21 Patients with TRD | None | Bilateral SCC-DBS applied  Stimulation parameters:  At implant: amplitude, 4.2 V;  pulse width, 91 µs; pulse frequency, 130.5 Hz;  6 mo postimplant: amplitude: 4.9 V;  Pulse width: 100.5 µs; pulse frequency, 130 Hz;  12 mo postimplant: amplitude, 5.2 V; pulse width, 93.9 µs; pulse frequency, 128.1 Hz | Suicidal ideation and suicide attempts | One patient had increased suicidal thoughts and attempted suicide after deactivation of DBS. |
| Malone et al, 2009 | Prospective open-label trial | Patients with TRD | 15 patients with TRD | None | Bilateral ventral capsule or ventral striatum DBS applied  Amplitude, 8-17 mA; pulse width, 90-210 µs; pulse frequency, 100-130 Hz | Suicidal ideation | Two patients had increased suicidal ideation compared with their prior ratings. |
| Porat et al, 2009 | Retrospective cohort study | 22 patients with Parkinson disease | 22 | None | Target was STN | Suicidal ideation and suicide attempts | Three patients had suicidal ideation before surgery.  Seven patients started to have suicidal ideation after surgery.  Nine patients in total had suicidal ideation after surgery (*P*=.046).  One patient did not endorse suicidal ideation before surgery. He completed suicide 2 months after surgery. |
| Puigdemont et al, 2012 | Randomized controlled and crossover trial | Patients with TRD | 8 patients with TRD | None | DBS was implanted to the subcallosal cingulate gyrus bilaterally with parameters of 3.6 V, 135 Hz, and 90 µs bandwidth | Suicide attempts | One patient attempted suicide 4 months after surgery. |
| Ramasubbu et al, 2020 | Double-blind, randomized, crossover trial | 22 patients with TRD | 10 patients with short pulse width DBS | 12 patients with long pulse width DBS | All patients were treated with SCC-DBS and 130 Hz  Long pulse width: amplitude, 3 V; pulse width, 210 µs. If there was less than 20% improvement in HDRS in monthly assessments, pulse width was gradually increased to 450 µs  Short pulse width: amplitude, 4 V; if there was less than 20% improvement, amplitude was increased gradually to 8 V; pulse width, constant 90 µs | Suicide completion | One patient completed suicide in the SPW group during the third postoperative month. This patient had a response to DBS, as measured by a 50% decrease in HDRS.  There was no significant difference between groups in terms of a reduction in HDRS scores at 6-month or 12-month follow-up. |
| Raymaekers et al, 2017 | Prospective, double-blind, crossover study | 7 patients with TRD | 7 | None | Target was either IC/BST or ITP.  Treatment parameters were unavailable | Suicide completion | Two patients completed suicide after a period of remission at 39 months and 79 months after surgery.  Both of these patients had previous suicide attempts. |
| Seijo Zazo et al, 2018 | Prospective cohort study | 30 patients with Parkinson disease | 30 | None | Bilateral STN stimulation;  stimulation parameters were adjusted until clinical optimization was achieved, as measured by a 30% decrease in UPDRS  Right contact: voltage: 3.1 (1.2-3.7) V; pulse width: 68.1 (60-120) µs; pulse frequency: 150 (90-185) Hz;  Left contact: voltage: 3.2 (2.0-3.7) V; pulse width, 68 (60-120) µs;  pulse frequency, 155 (90-185) Hz | Suicidal ideation | No significant difference was shown between baseline (on medication) and at 6-month and 12-month follow-up regarding suicidal ideation. |
| Smeding et al, 2006 | Prospective cohort study | Patients with Parkinson disease | 99 patients had STN | 39 control patients | Bilateral STN-DBS applied  Treatment parameters were unavailable | Suicide attempts | There was 1 suicide attempt in the STN group. |
| Soulas et al, 2008 | Retrospective cohort study | 200 patients with Parkinson disease | 200 | None | Bilateral STN-DBS implanted | Suicide completions | Two patients completed suicide, and 4 patients attempted suicide.  No significant relationship shown between change in stimulator settings and completed suicide. |
| Strutt et al, 2012 | Retrospective cohort study | 39 patients with Parkinson disease | 17 | 22 | Bilateral STN-DBS implanted  Treatment parameters were unavailable | Suicide completions | In the DBS group, 1 patient completed suicide. The patient had a remarkably worse mood after the surgery without any apparent stressors. |
| Toft et al, 2011 | Retrospective cohort study | 144 patients with Parkinson disease | 144 | None | Target was bilateral STN  Right: voltage, 3.1±0.6 V; pulse width, 61±6 µs; pulse frequency, 158±22 Hz;  Left: voltage, 3.3±0.6 V; pulse width, 62±8 µs; pulse frequency, 158±22 Hz | Suicide completions | Two of the 144 patients completed suicide at 15 and 16 months after surgery. |
| Umemura et al, 2011 | Retrospective cohort study | 180 patients with Parkinson disease | 180 | None | Bilateral STN targeted  Pulse width, 90 µs; pulse rate, 130 Hz; amplitude, 2-3 V | Suicide attempts | Two patients attempted suicide. |
| van der Wal et al, 2020 | Retrospective cohort study | 25 patients with TRD treated with vALIC DBS in a double-blind, sham-controlled, crossover trial with maintenance therapy during the study | 25 | None | All patients treated with vALIC  Amplitude, 2.5-6.0 V; pulse width and frequency, 90 µs and 130 or 180 Hz, respectively | Suicide attempts | During the study, 1 patient in the nonresponder group attempted suicide. The changes in HDRS (*P*=.90) or MADRS scores (*P*=.35) during the maintenance phase were not significant for responders or nonresponders. |
| Volkmann et al, 2012 | Randomized controlled trial | Patients with generalized segmental dystonia | 20 patients assigned to active stimulation within the first 3 mo | 20 patients assigned to sham stimulation within the first 3 mo | GPi was stimulated bilaterally  Treatment parameters: amplitude, 3.5 V; pulse width, 125 µs; pulse frequency, 146.9 Hz  Treatment parameters did not change significantly from baseline values at 3 y and 5 y follow-up or between 3 y and 5 y follow-up | Suicide attempts | One patient attempted suicide 6 months after surgery during a depressive episode. After inpatient treatment, the patient did not have psychiatric symptoms. |
| Voon et al, 2008 | Retrospective cohort study | 5,311 patients with Parkinson disease | 5,311 | None | Target was STN  Treatment parameters were unavailable | Suicide attempts and suicide completions | The attempted suicide rate was 0.9% (48/5,311), and the completed suicide rate was 0.45% (24/5,311).  The suicide completion rate in the first postoperative year was 0.26%, which was significantly higher than the expected suicide completion rate when it was controlled for age, sex, and country-specific rates (*P<.*001), and it remained significantly higher than the age- and sex-matched group at 4 years of follow-up.  Postoperative depression and apathy, being single, history of impulse-control disorders (*P<.*01 for all), previous attempts, younger age at Parkinson diagnosis, and percent LEDD decrease (*P<.*05 for all) were shown to significantly predict suicide attempt risk.  Treatment parameters were not a significant predictor of suicide attempt.  Almost half of the suicide completers or suicide attempters reported suicidal ideation before the events. |
| Weintraub et al, 2013 | Randomized controlled trial | 255 patients with Parkinson disease | Phase 1: 121 patients treated with DBS;  Phase 2: 147 patients randomized to STN | Phase 1: 134 patients treated with BMT;  Phase 2: 152 patients randomized to GPi | Phase 1: DBS vs BMT;  Phase 2: DBS STN vs DBS GPi;  Treatment parameters were unavailable | Suicidal ideation and suicidal behavior | Phase 1: 3 months after surgery, no patient had suicidal ideation; 6 months after surgery, there was no significant difference between groups regarding new-onset suicidal ideation.  During the all-randomization phase, there were no reported suicidal behaviors.  Phase 2: 6 months after surgery, there were no significant differences between groups regarding new-onset suicidal ideation (1.5% vs 0.7%) or suicidal behaviors.  Phase 1 and phase 2:  During 2 years of follow-up, there was 1 suicide attempt and 1 completed suicide. The postoperative periods for both patients were complicated with complex medical and neurologic problems. |
| Williams et al, 2010 | Open-label trıal | 366 patients with Parkinson disease | 183 | 183 | Except for 2 patients, all patients had a therapy target of bilateral STN  Treatment parameters were unavailable | Suicide attempts | In the DBS group, 1 patient who had a suicide attempt before surgery completed suicide in the postoperative period. |
| Witt et al, 2008 | Randomized controlled trial | Patients with Parkinson disease | 60 patients treated with STN-DBS | 63 patients treated with BMT | Bilateral STN was stimulated  Treatment parameters: pulse width, 60 µs; pulse frequency, 130 Hz;  Amplitude adjusted for each patient | Suicide completion | In the DBS group, 1 patient completed suicide. In the BMT group, no patients completed suicide. |

Abbreviations: BDI, Beck Depression Inventory; BMT, best medical treatment; DBS, deep brain stimulation; GPi, globus pallidus interna; HDRS, Hamilton Depression Rating Scale; IC/BST, internal capsule/bed nucleus of the stria terminalis; ITP, inferior thalamic peduncle; LEDD, levodopa equivalent daily dose; MADRS, Montgomery-Asberg Depression Rating Scale; MDD, major depressive disorder; MDE, major depressive episode; MINI, Mini International Neuropsychiatric Interview; SCC, subcallosal cingulate; SPW, short pulse width; STN, subthalamic nucleus; TRD, treatment-resistant depression; UPDRS III, Unified Parkinson Disease Rating Scale; vALIC, ventral anterior limb of internal capsule; VIM, ventral intermediate.

^a^ Clarity lacking in the report regarding whether the patients who were suicidal and attempting suicide were the same patients.
